# Supplementary material for: Optimization and Antibacterial Evaluation of Novel 3-(5-Fluoropyridine-3-yl)-2-oxazolidinone Derivatives Containing a Pyrimidine Substituted Piperazine
Source: Molecules. 2023 May 23;28(11):4267. doi: 10.3390/molecules28114267 (PMC10254757; doi:10.3390/molecules28114267)
Supplement: Supplementary file 1 [file molecules-28-04267-s001.zip › molecules-2345611-supplementary.pdf]

Supplementary Materials

Optimization and Antibacterial Evaluation of Novel 3-(5-Fluoropyridine-3-yl)-2-oxazolidinone Derivatives Containing a Pyrimidine Substituted Piperazine

Xin Wang [1], Bo Jin [1], Yutong Han [1], Tong Wang [1], Zunlai Sheng [1,2], Ye Tao [1,2] and Hongliang Yang [1,2,*]

[1] Department of Veterinary Medicine, Northeast Agricultural University, Harbin 150030, China; 13331613023@163.com (X.W.); cnborgin@163.com (B.J.); hanyutong1@163.com (Y.H.); wt1282808784@163.com (T.W.); shengzunlai@neau.edu.cn (Z.S.); taoye062@126.com (Y.T.)

[2] Heilongjiang Key Laboratory for Animal Disease Control and Pharmaceutical Development, Harbin 150030, China

* Correspondence: hongl_yang@126.com.

1. Compounds spectral data

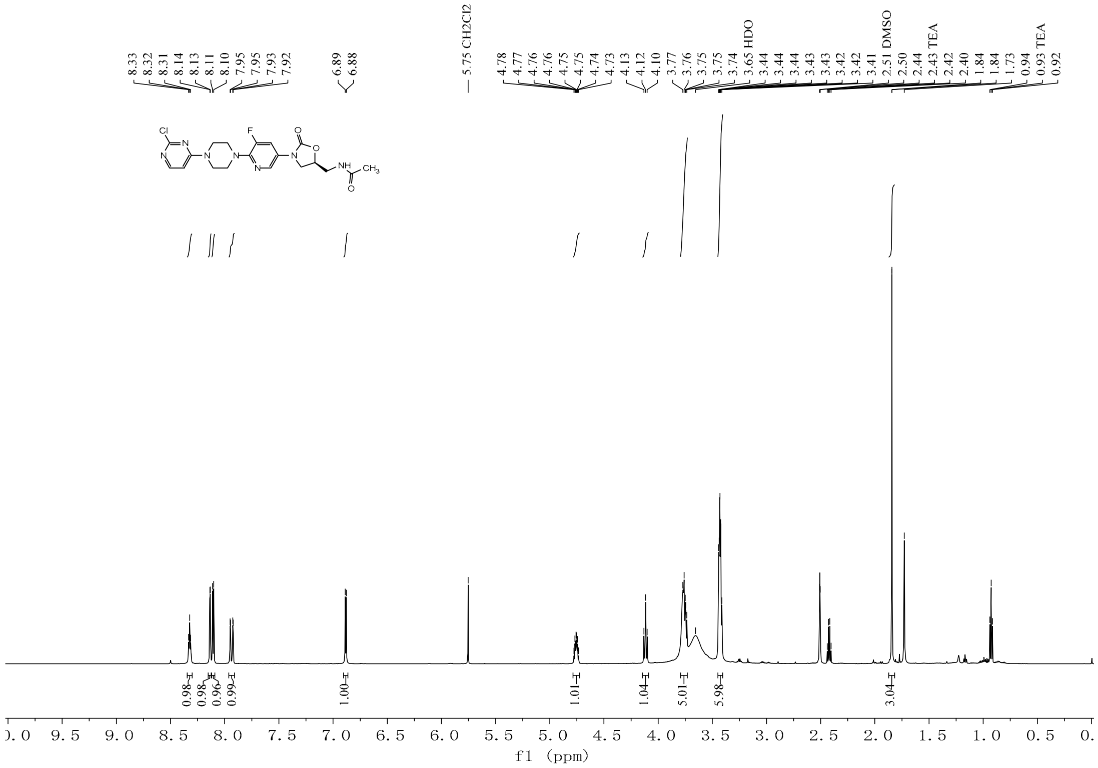

Figure S1. ^{1}H NMR Spectrum (DMSO-d_6, 600 MHz) of **5**.

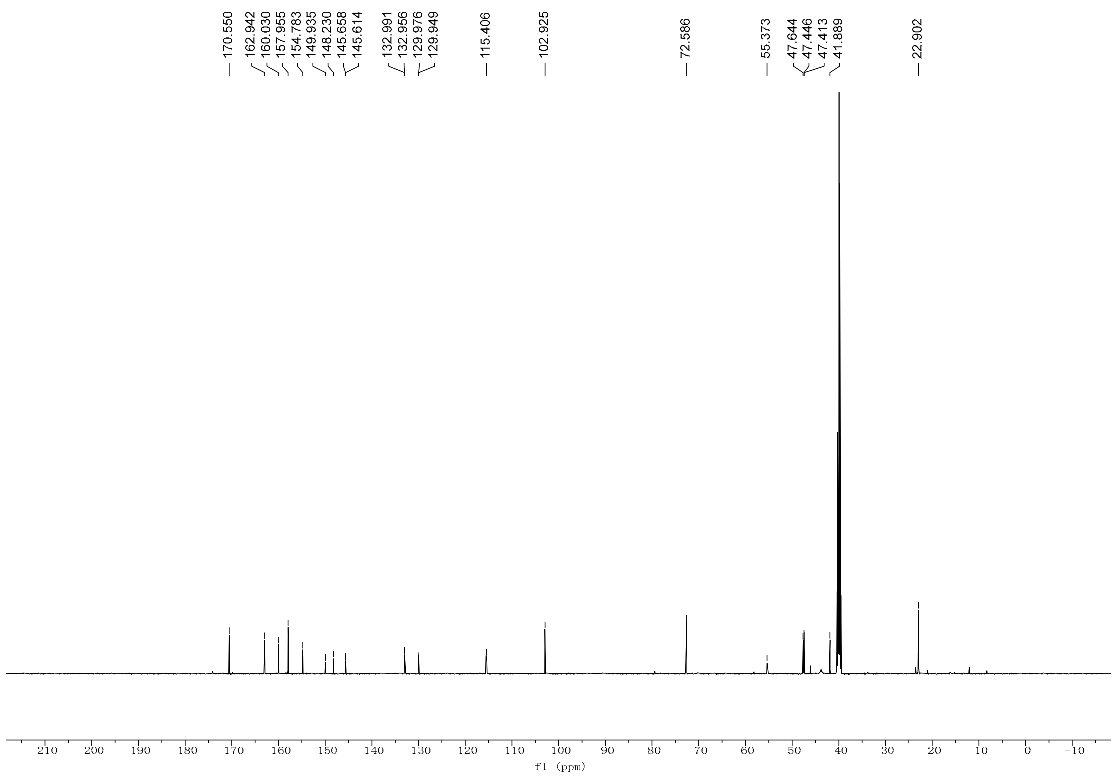

Figure S2. ^{13}C NMR Spectrum (DMSO-d_6, 150 MHz) of **5**.

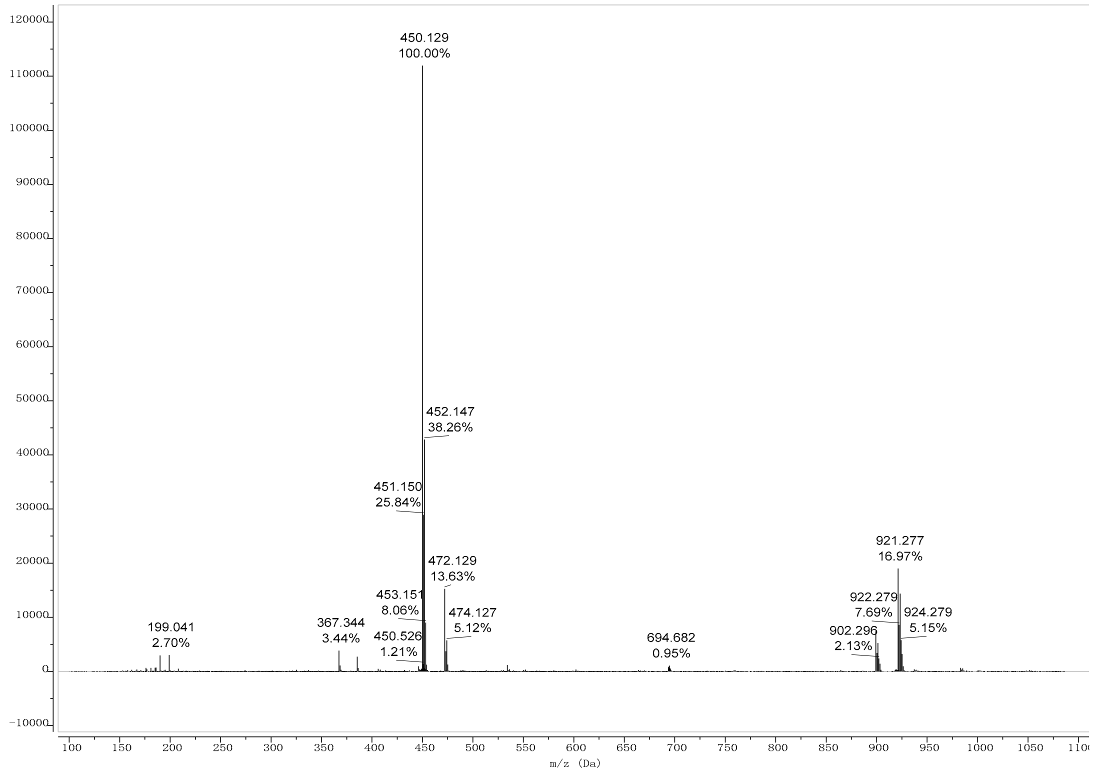

Figure S3. MS for $C_{19}H_{21}ClFN_7O_3$ (Mwt.: 449.87): m/z 450.129 ([M+H] $^+$, bp) of **5**.

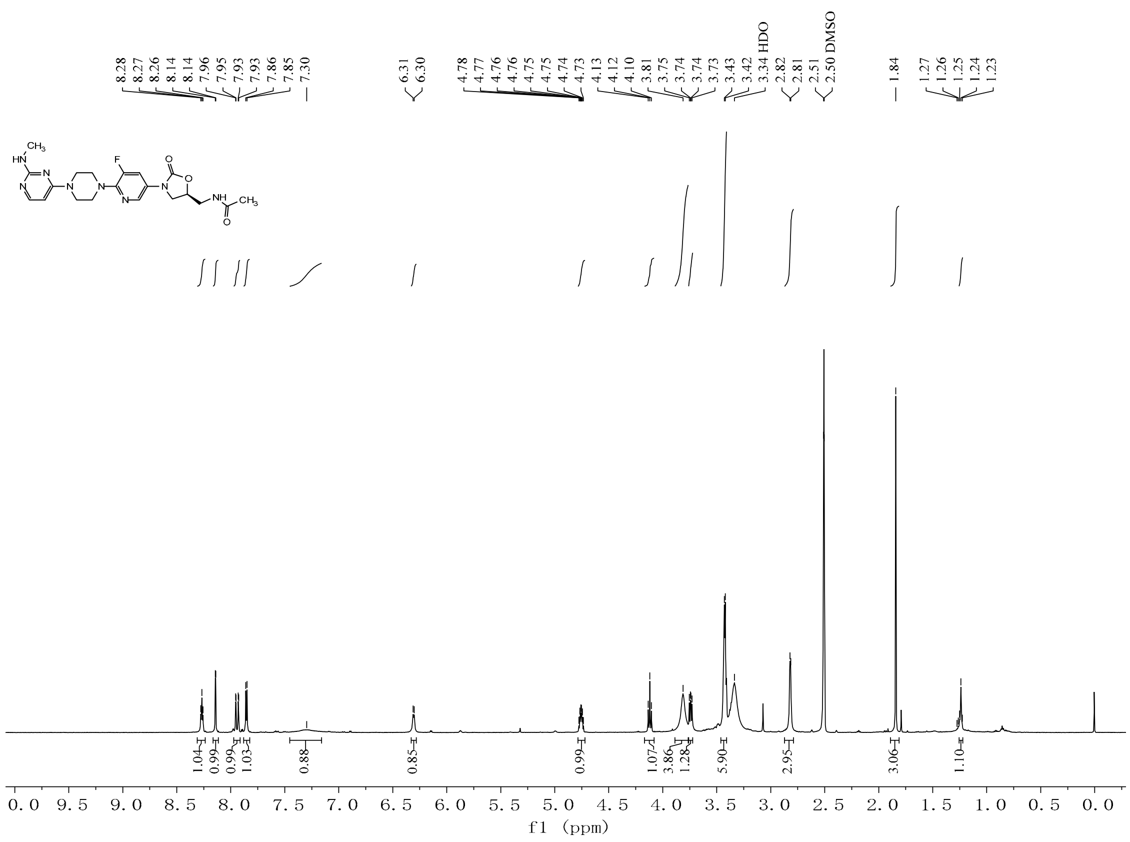

Figure S4. ^{1}H NMR Spectrum (DMSO-d_6, 600 MHz) of **6a**.

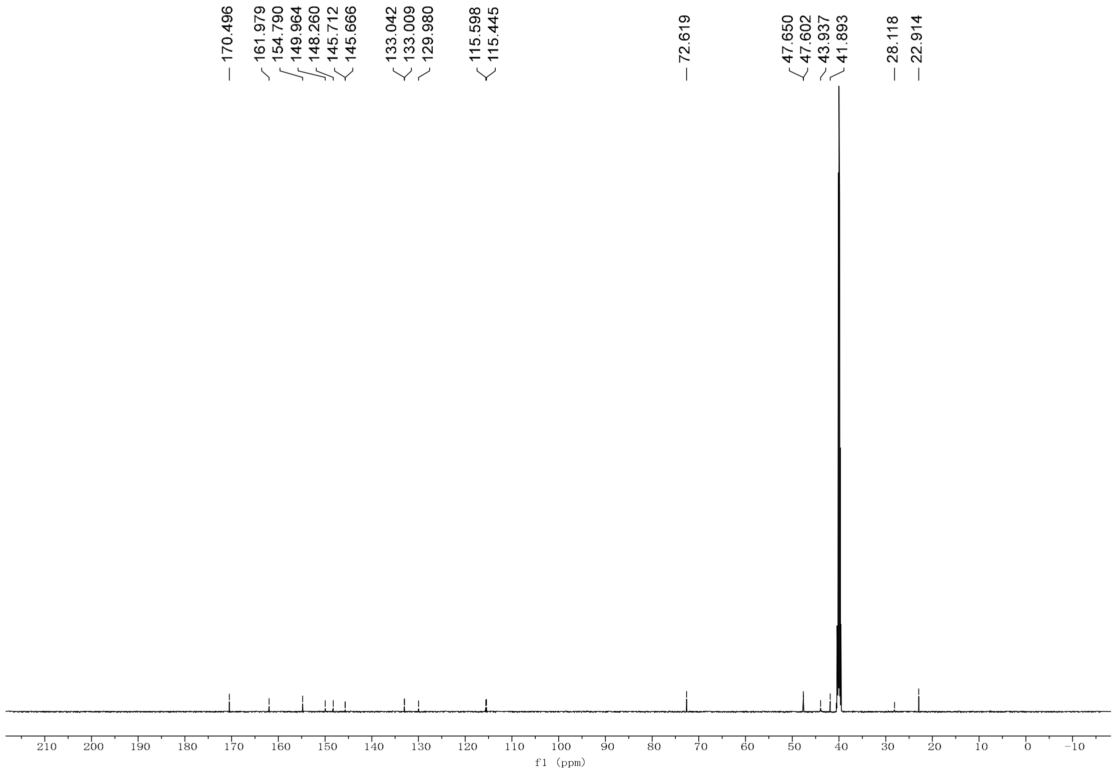

Figure S5. ^{13}C NMR Spectrum (DMSO-d_6, 150 MHz) of **6a**.

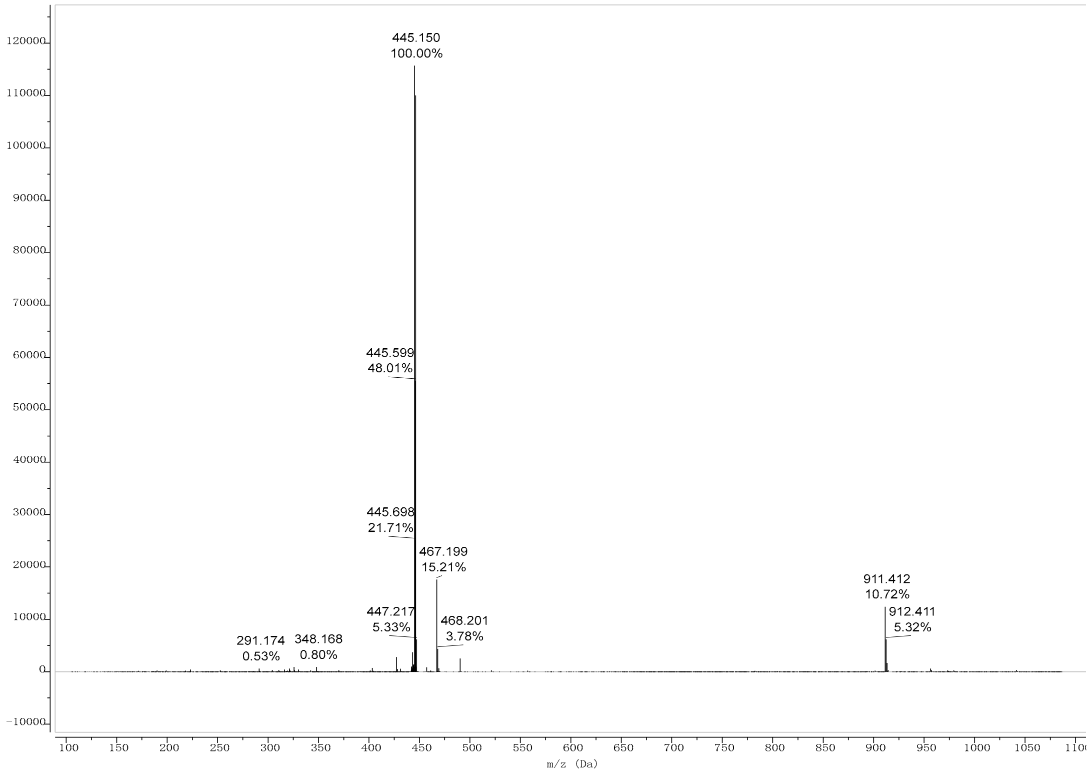

Figure S6. MS for $C_{20}H_{25}FN_8O_3$ (Mwt.: 444.47): m/z 445.150 ([M+H] $^+$, bp) of **6a**.

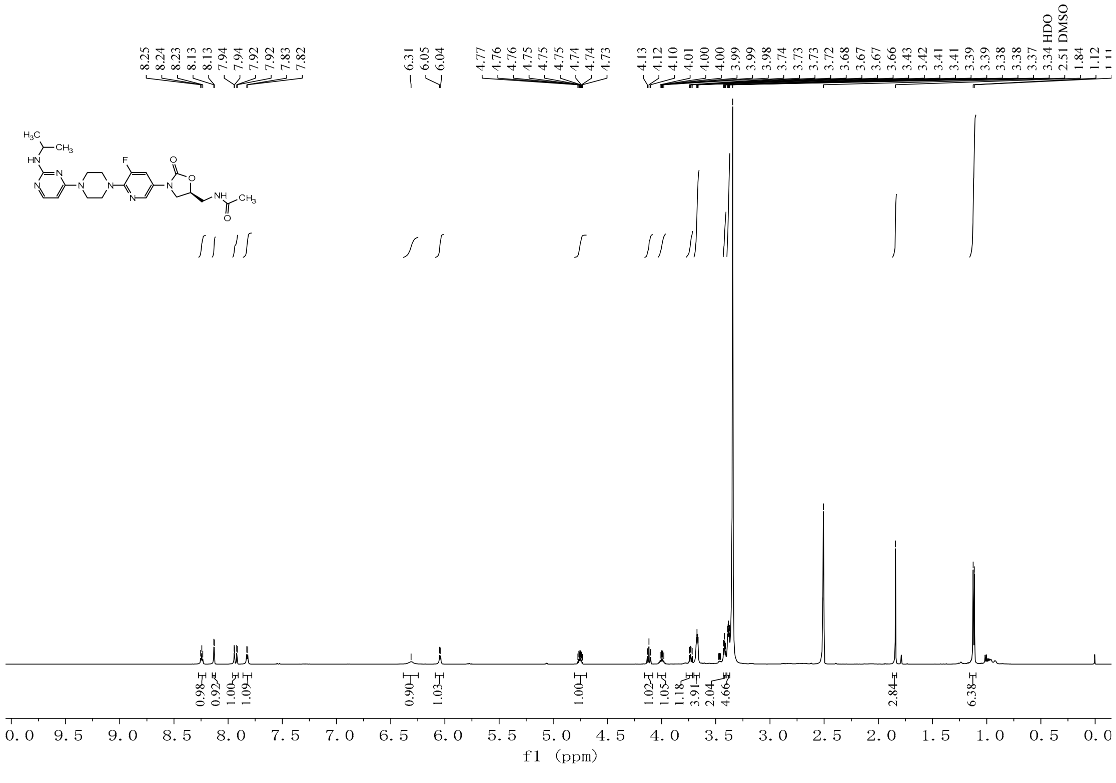

Figure S7. ^{1}H NMR Spectrum (DMSO-d_6, 600 MHz) of **6b**.

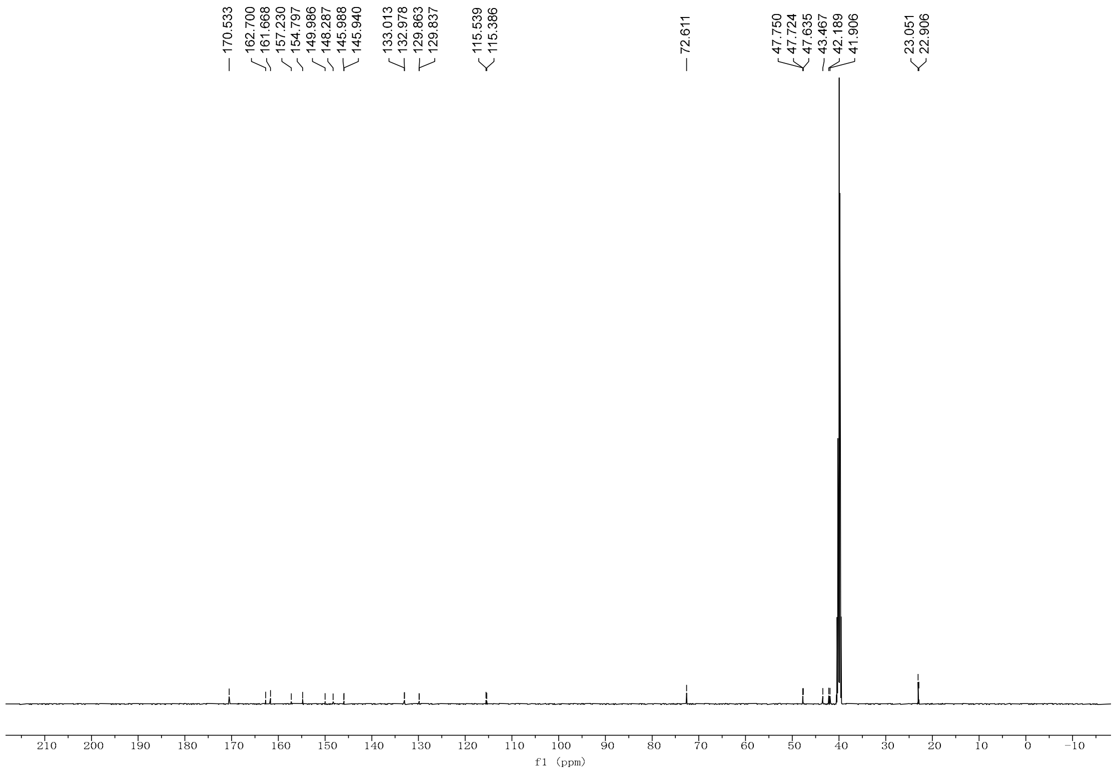

Figure S8. ^{13}C NMR Spectrum (DMSO-d_6, 150 MHz) of **6b**.

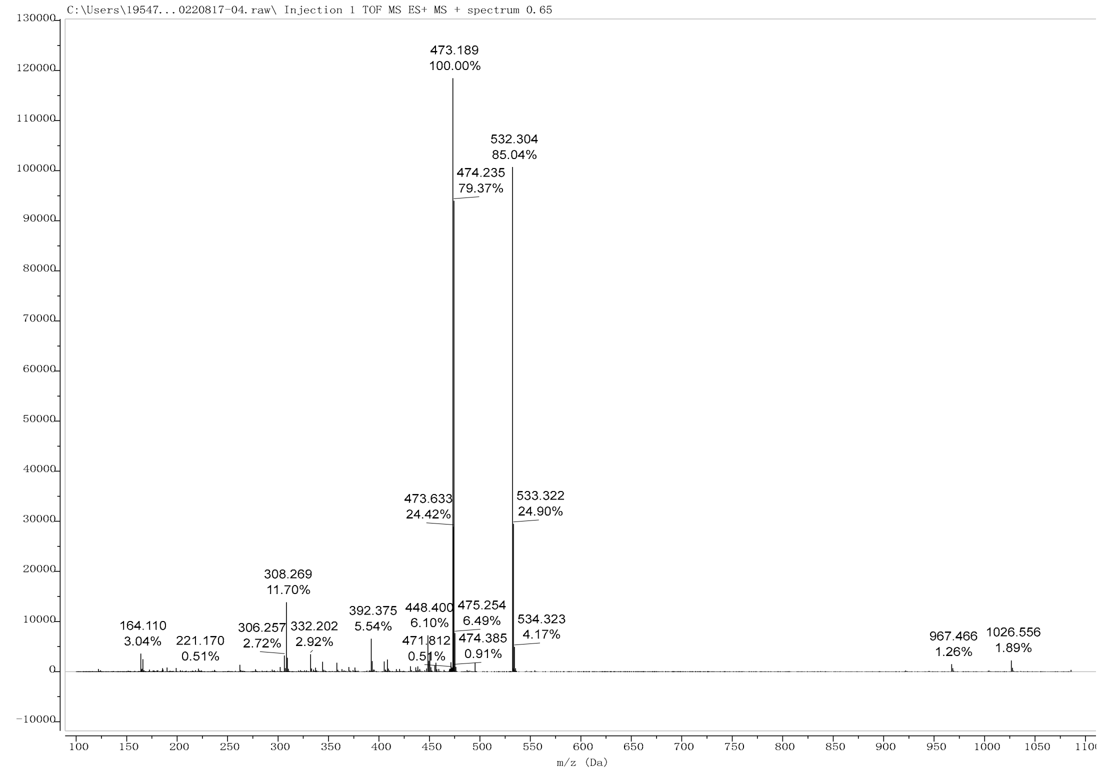

Figure S9. MS for $C_{22}H_{29}FN_8O_3$ (Mwt.: 472.53): m/z 473.189 ($[M+H]^+$, bp) of **6b**.

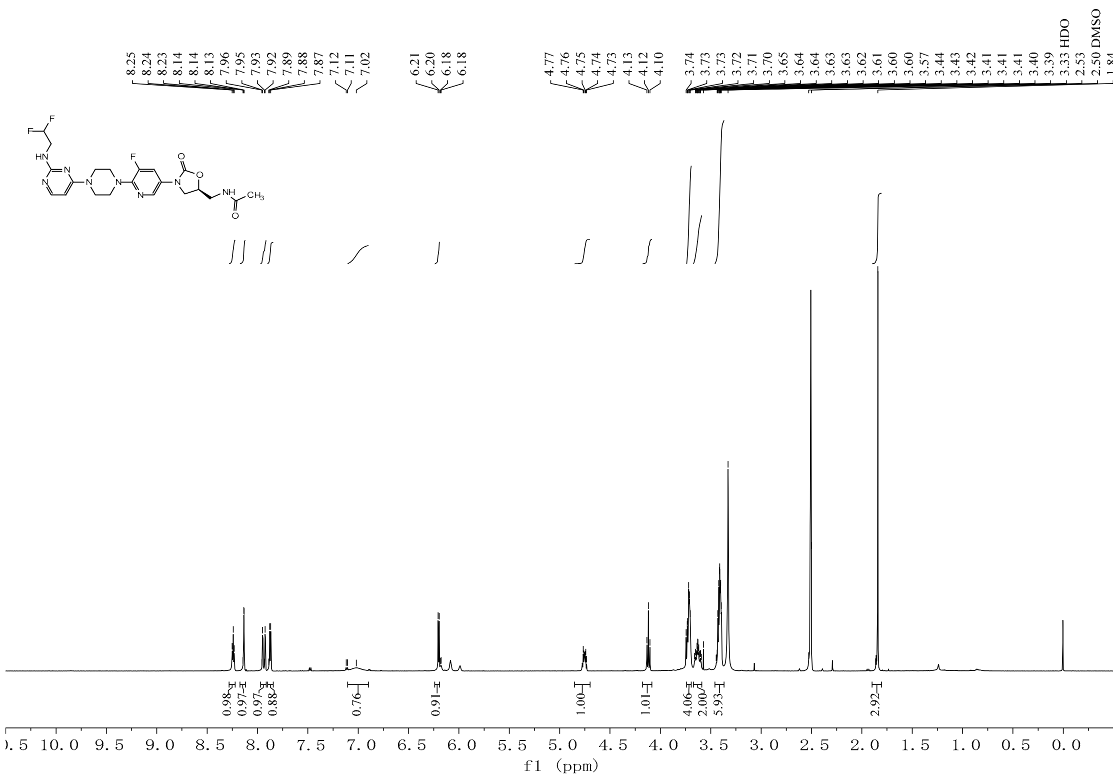

Figure S10. ^{1}H NMR Spectrum (DMSO-d_6, 600 MHz) of **6c**.

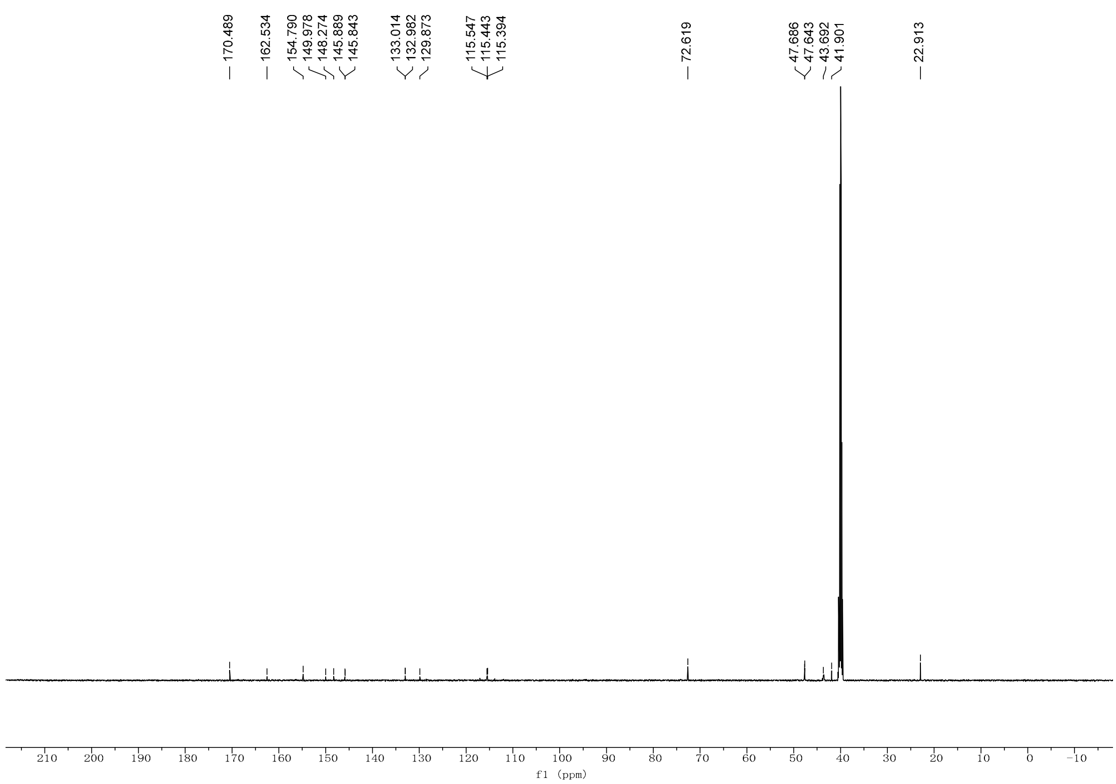

Figure S11. ^{13}C NMR Spectrum (DMSO-d_6, 150 MHz) of **6c**.

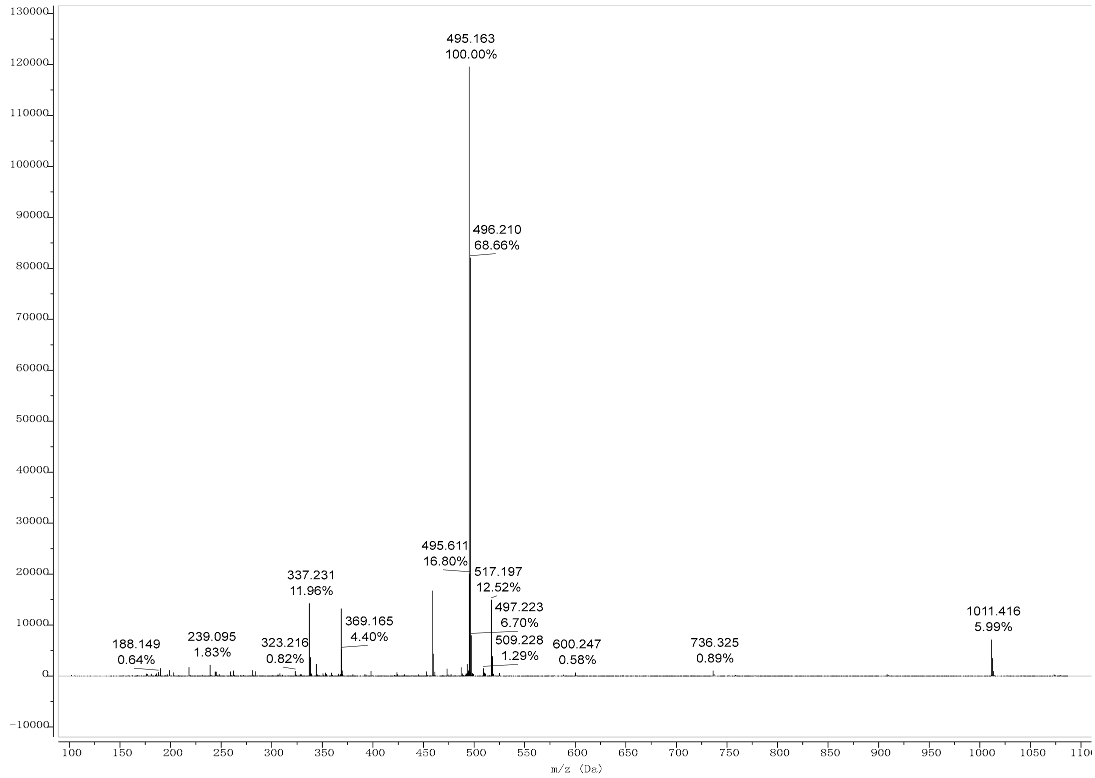

Figure S12. MS for $C_{21}H_{25}F_3N_8O_3$ (Mwt.: 494.48): m/z 495.163 ([M+H] $^{+}$, bp) of **6c**.

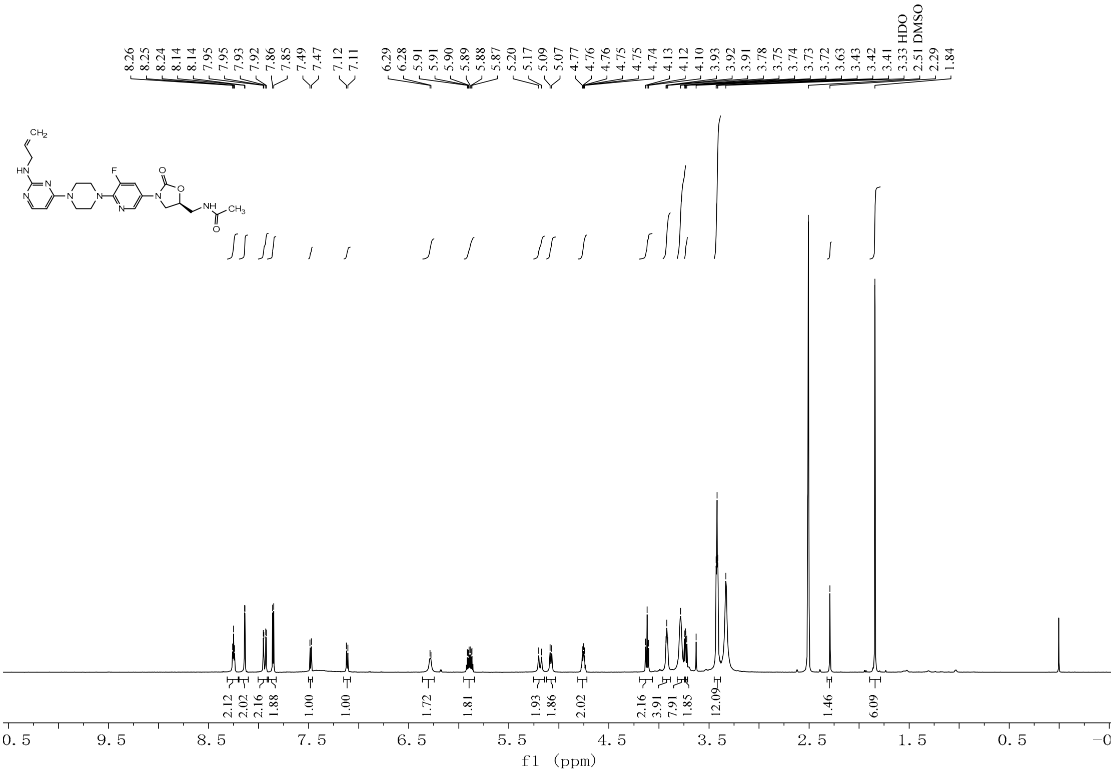

Figure S13. ^{1}H NMR Spectrum (DMSO-d_6, 600 MHz) of **6d**.

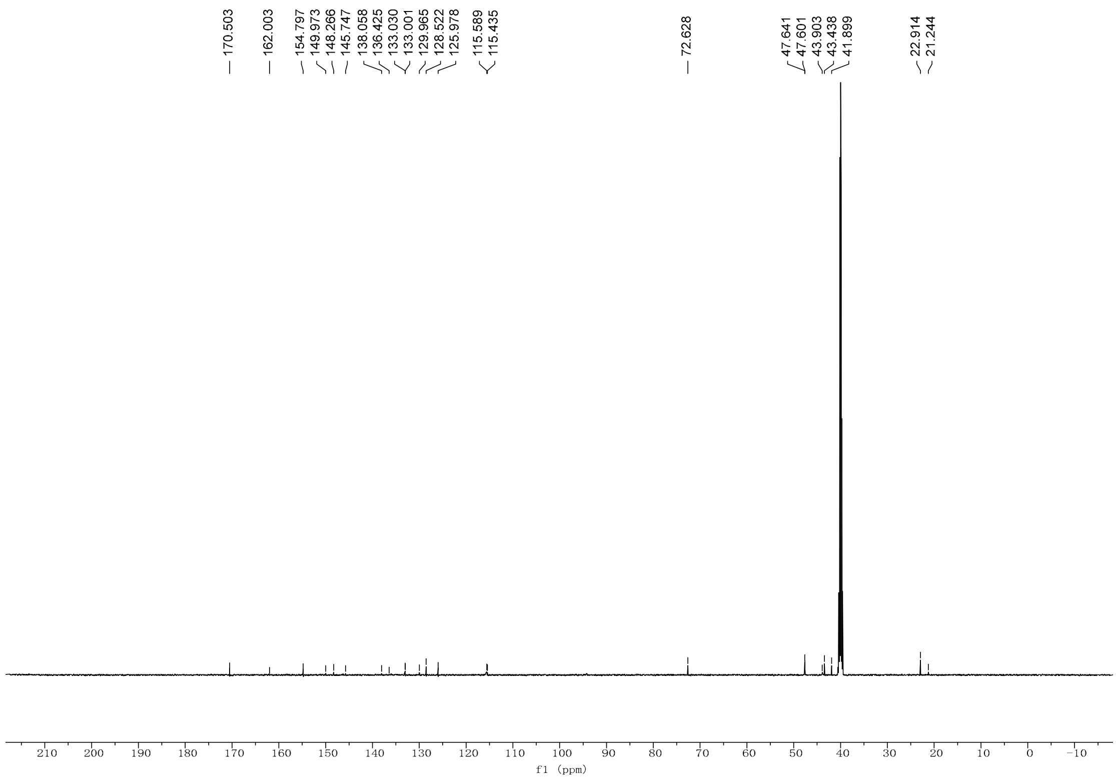

Figure S14. ^{13}C NMR Spectrum (DMSO-d_6, 150 MHz) of **6d**.

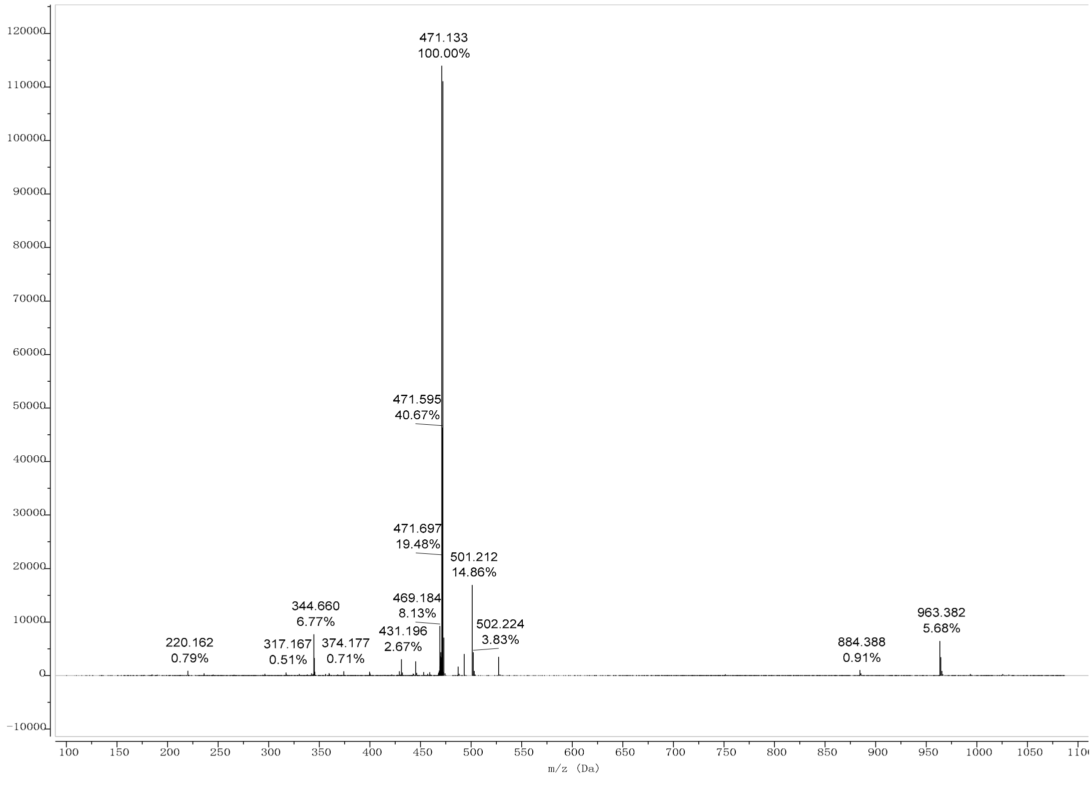

Figure S15. MS for $C_{22}H_{27}FN_8O_3$ (Mwt.: 470.51): m/z 471.133 ([M+H] $^+$, bp) of **6d**.

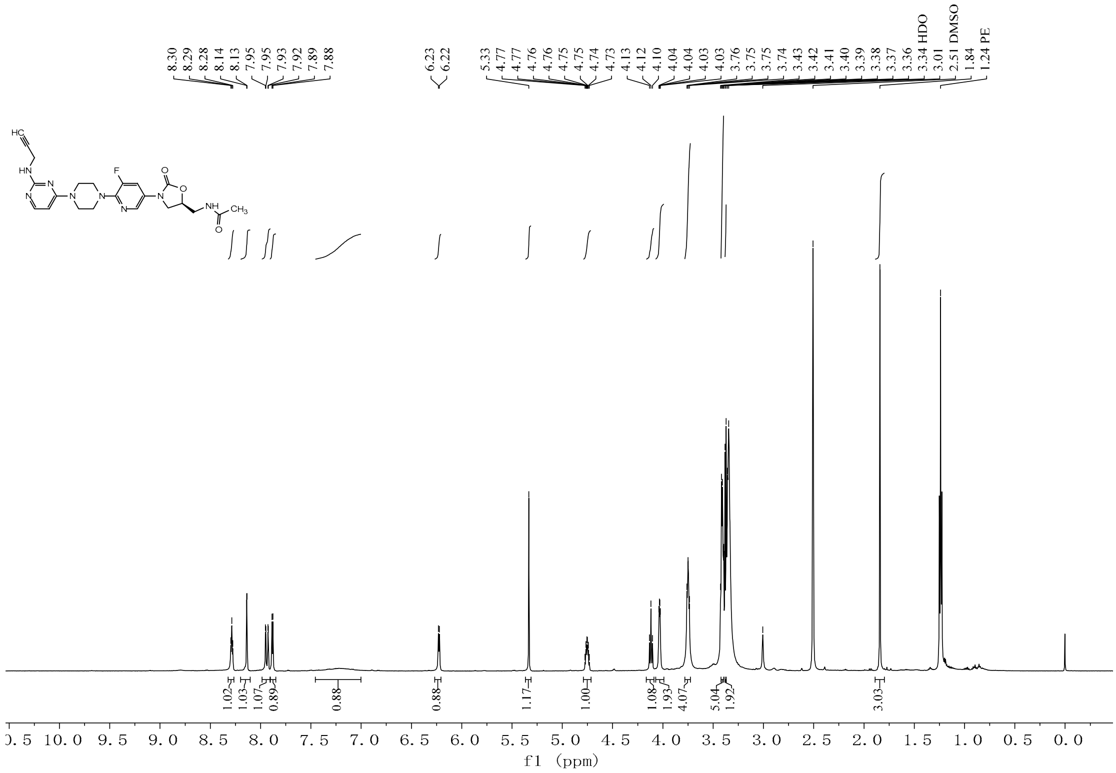

Figure S16. ^{1}H NMR Spectrum (DMSO-d_6, 600 MHz) of **6e**.

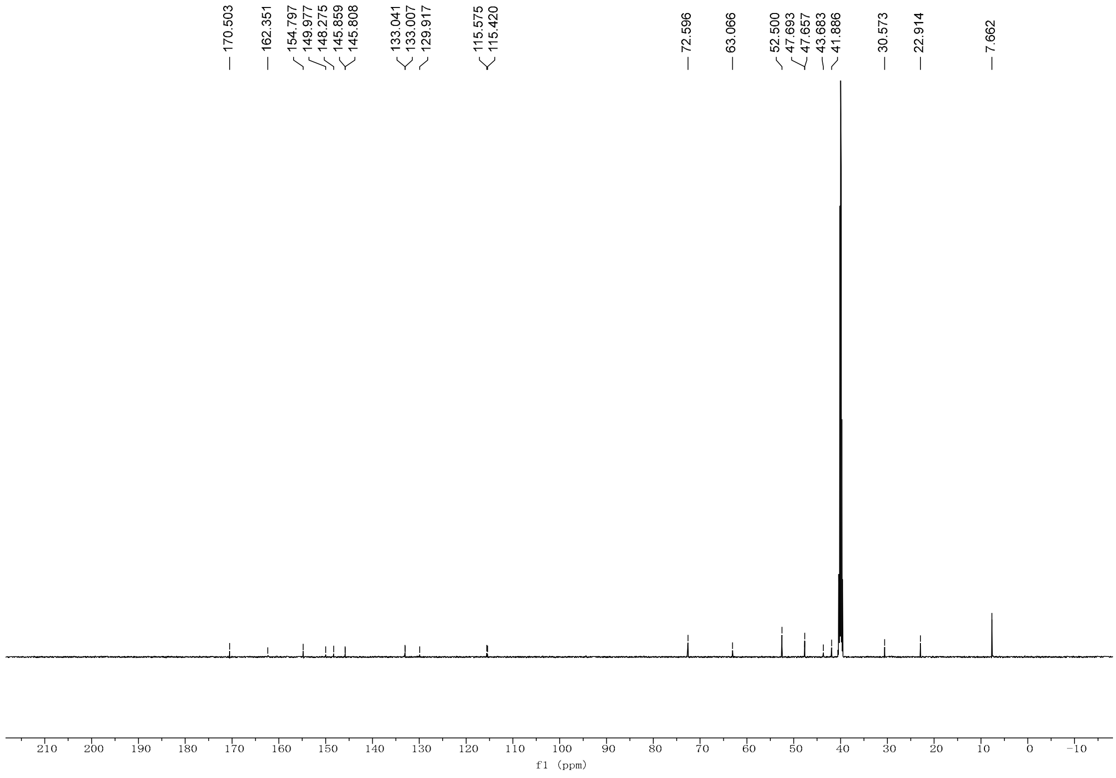

Figure S17. ^{13}C NMR Spectrum (DMSO-d_6, 150 MHz) of **6e**.

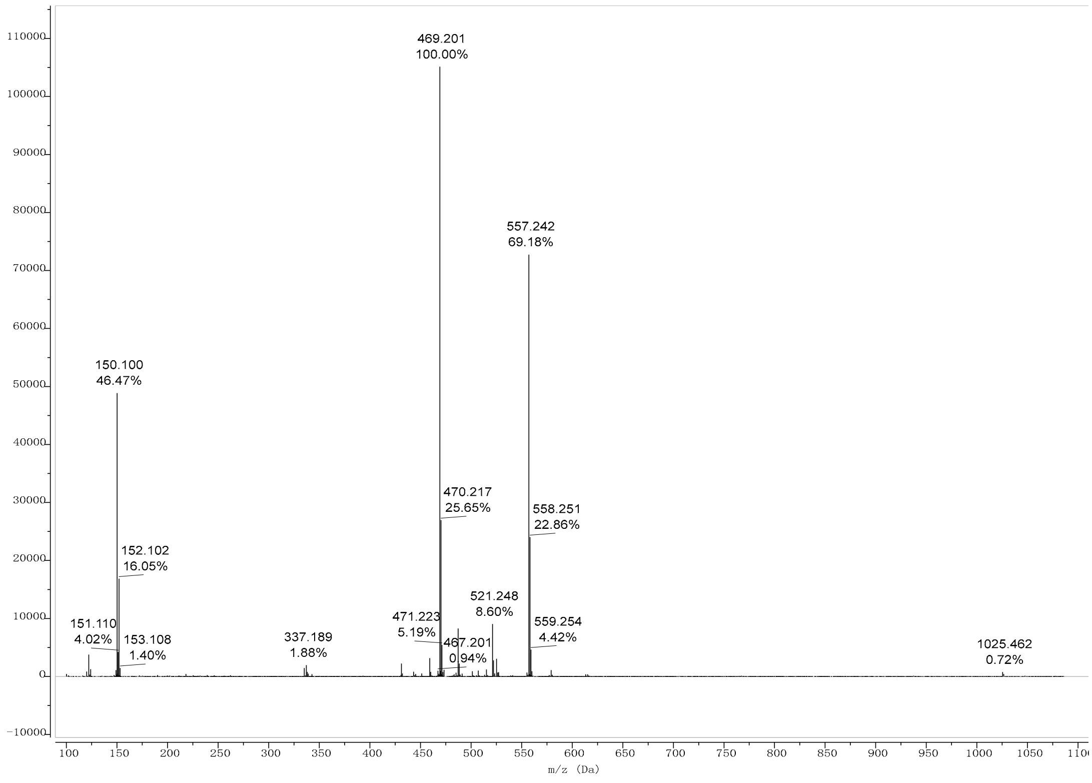

Figure S18. MS for $C_{22}H_{25}FN_8O_3$ (Mwt.: 468.49): m/z 469.201 ([M+H] $^+$, bp) of **6e**.

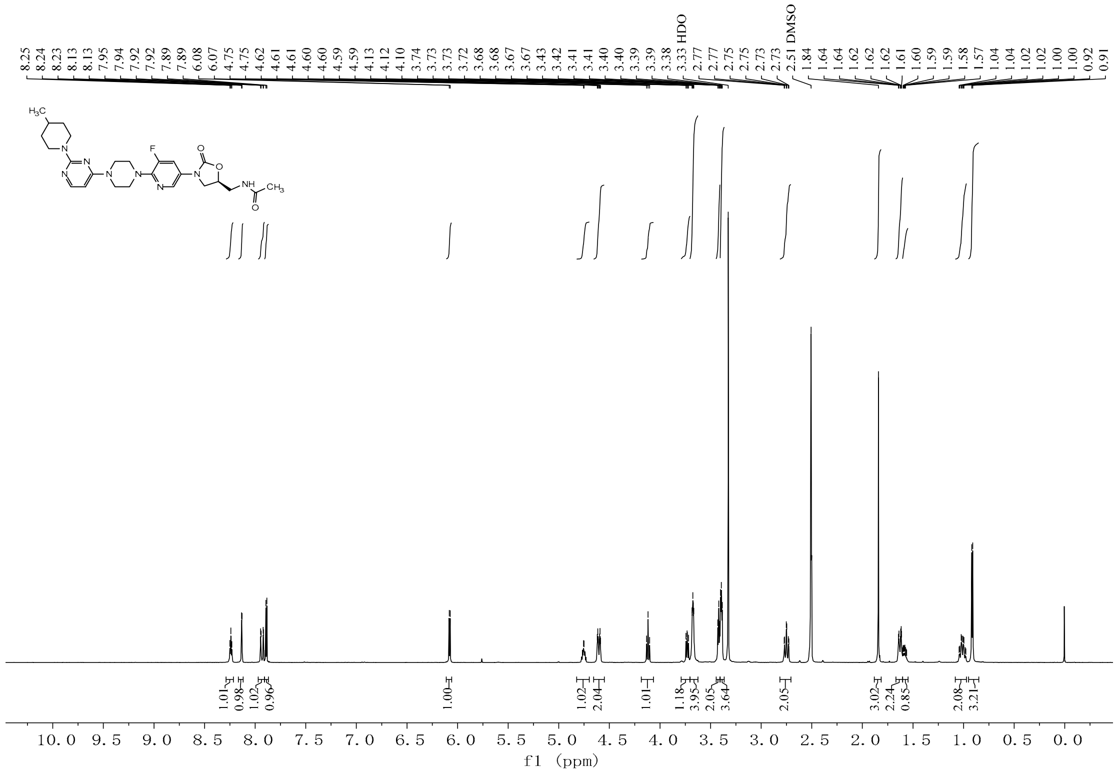

Figure S19. ^{1}H NMR Spectrum (DMSO-d_6, 600 MHz) of **6f**.

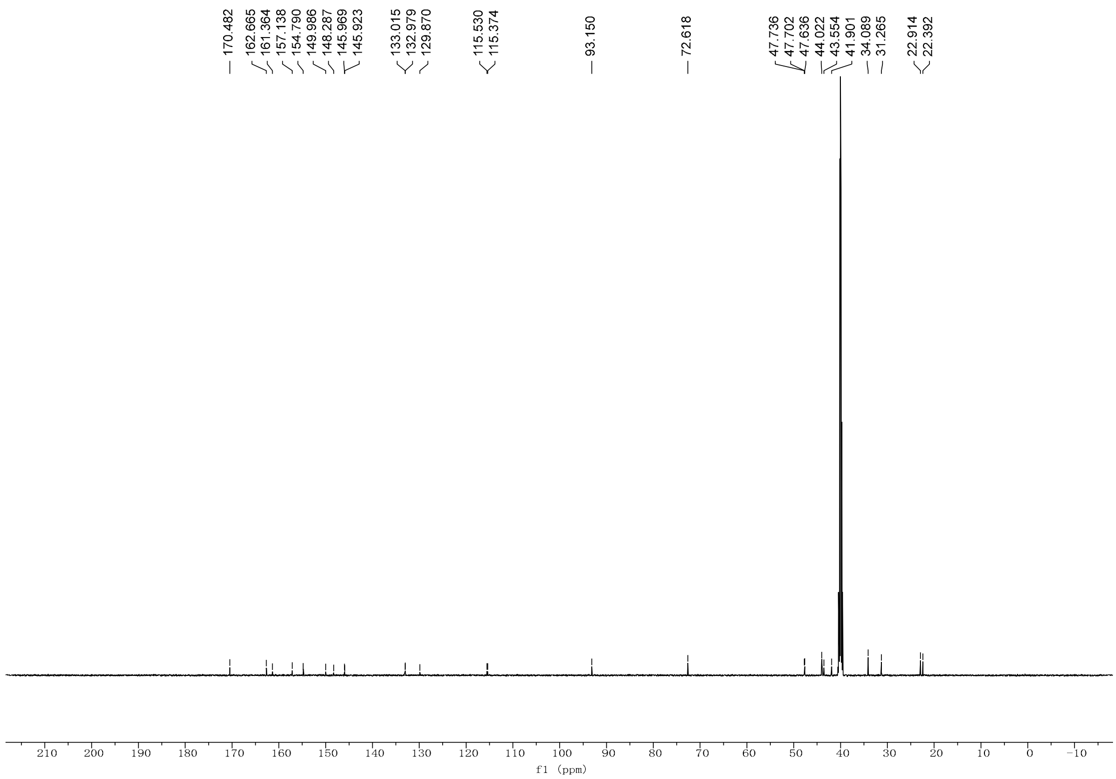

Figure S20. ^{13}C NMR Spectrum (DMSO-d_6, 150 MHz) of **6f**.

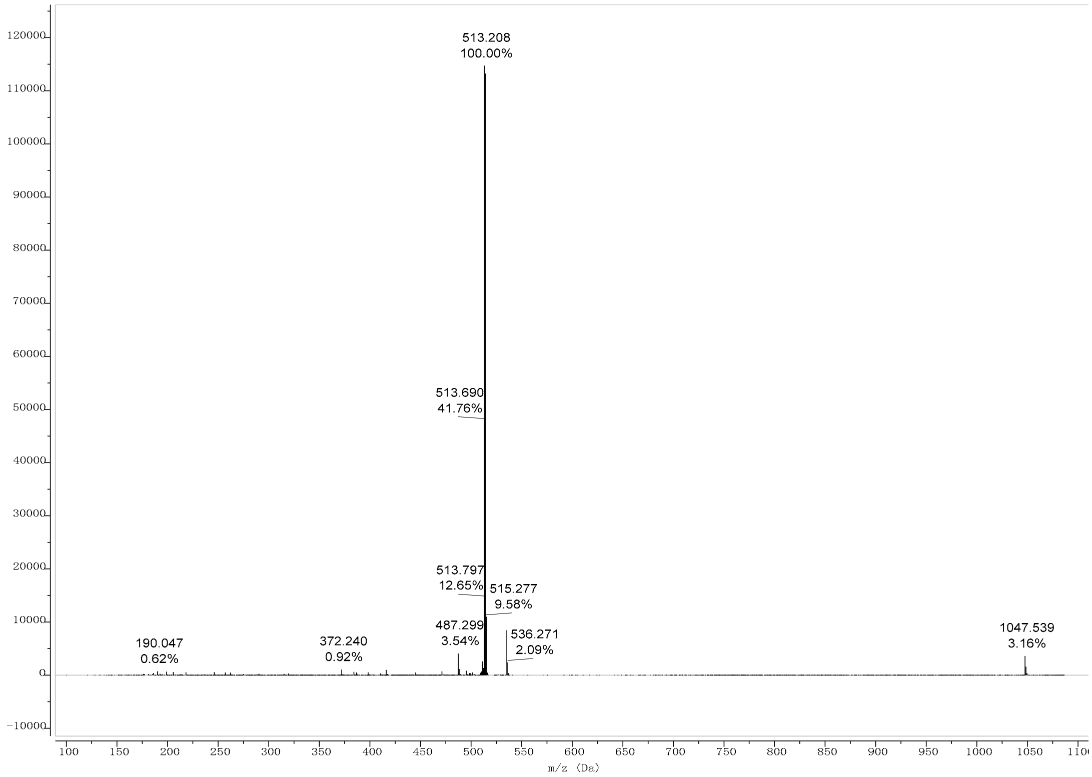

Figure S21. MS for $C_{25}H_{33}FN_8O_3$ (Mwt.: 512.59): m/z 513.208 ([M+H] $^+$, bp) of **6f**.

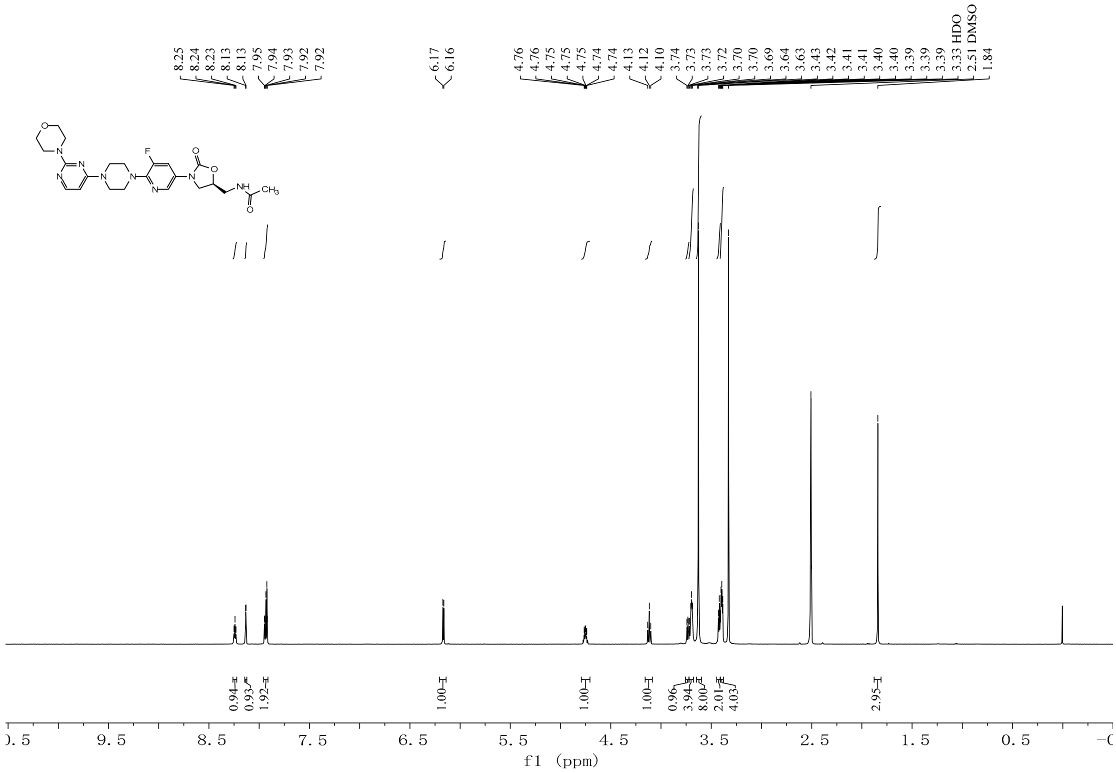

Figure S22. ^{1}H NMR Spectrum (DMSO-d_6, 600 MHz) of **6g**.

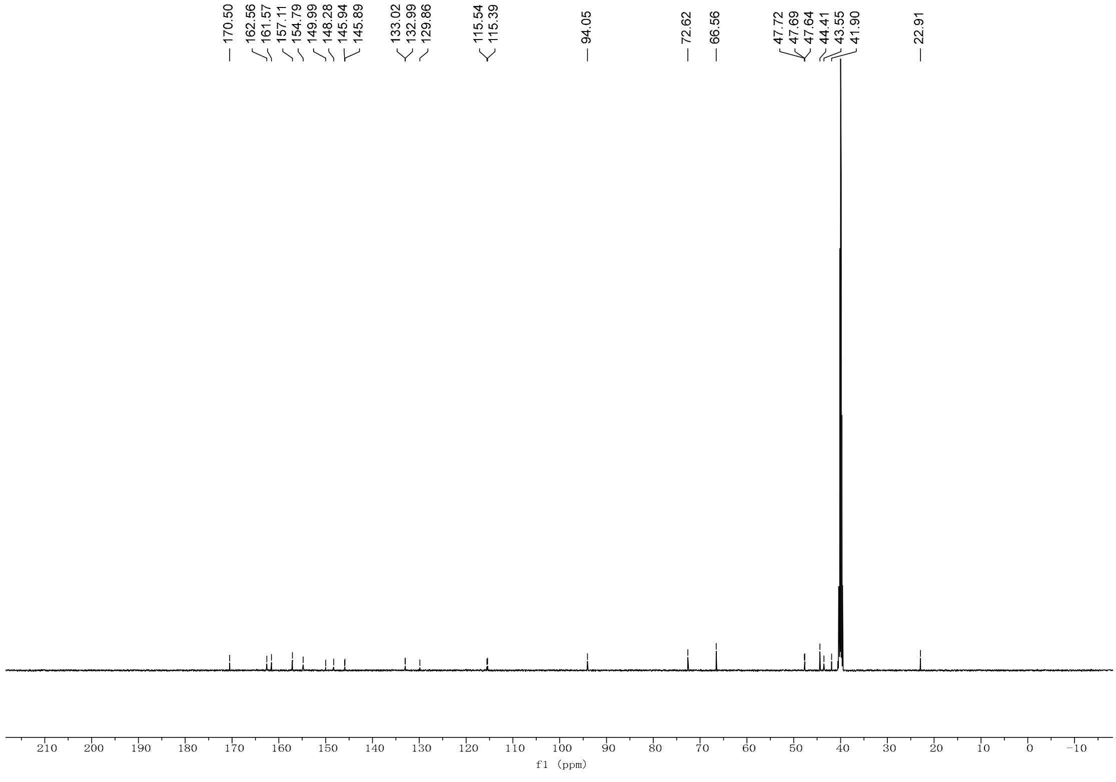

Figure S23. ^{13}C NMR Spectrum (DMSO-d_6, 150 MHz) of **6g**.

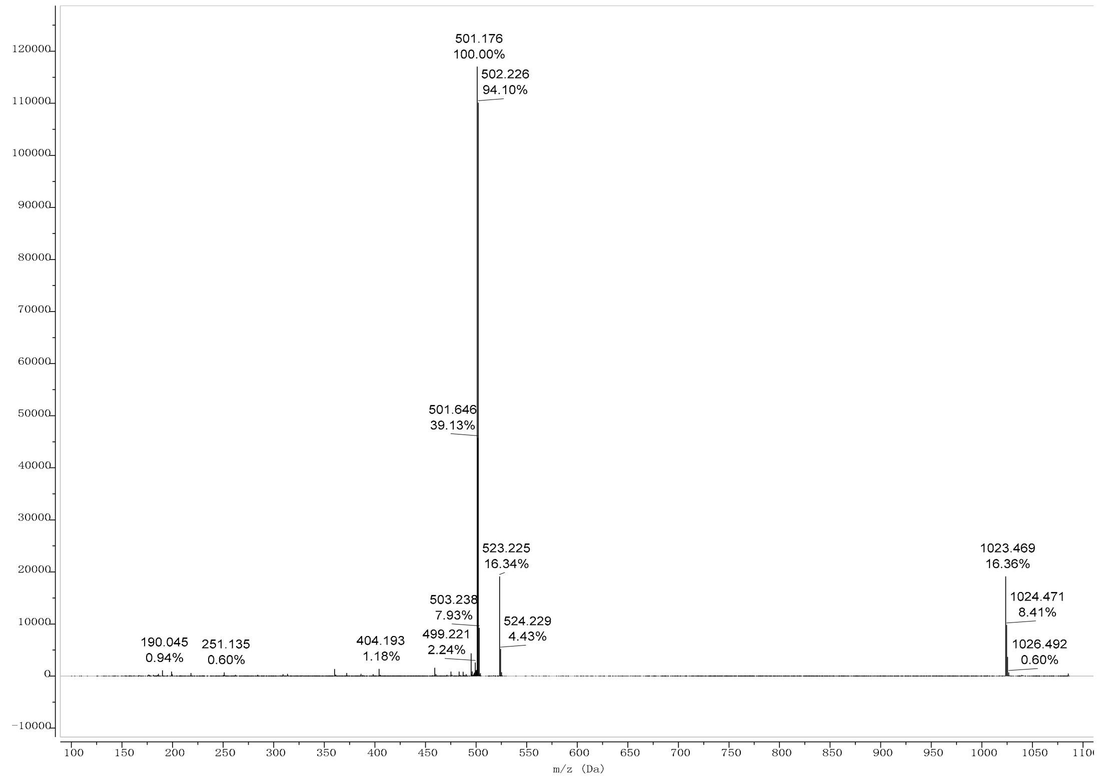

Figure S24. MS for $C_{23}H_{29}FN_8O_4$ (Mwt.: 500.54): m/z 501.176 ([M+H] $^+$, bp) of **6g**.

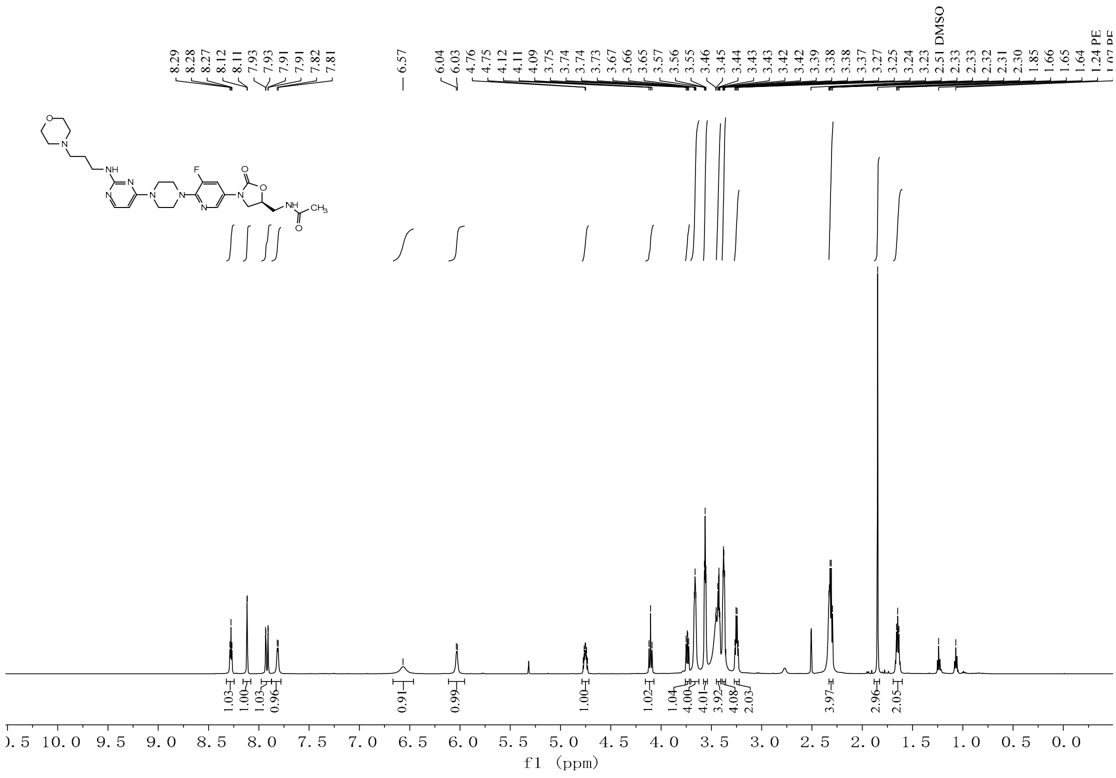

Figure S25. ^{1}H NMR Spectrum (DMSO-d_6, 600 MHz) of **6h**.

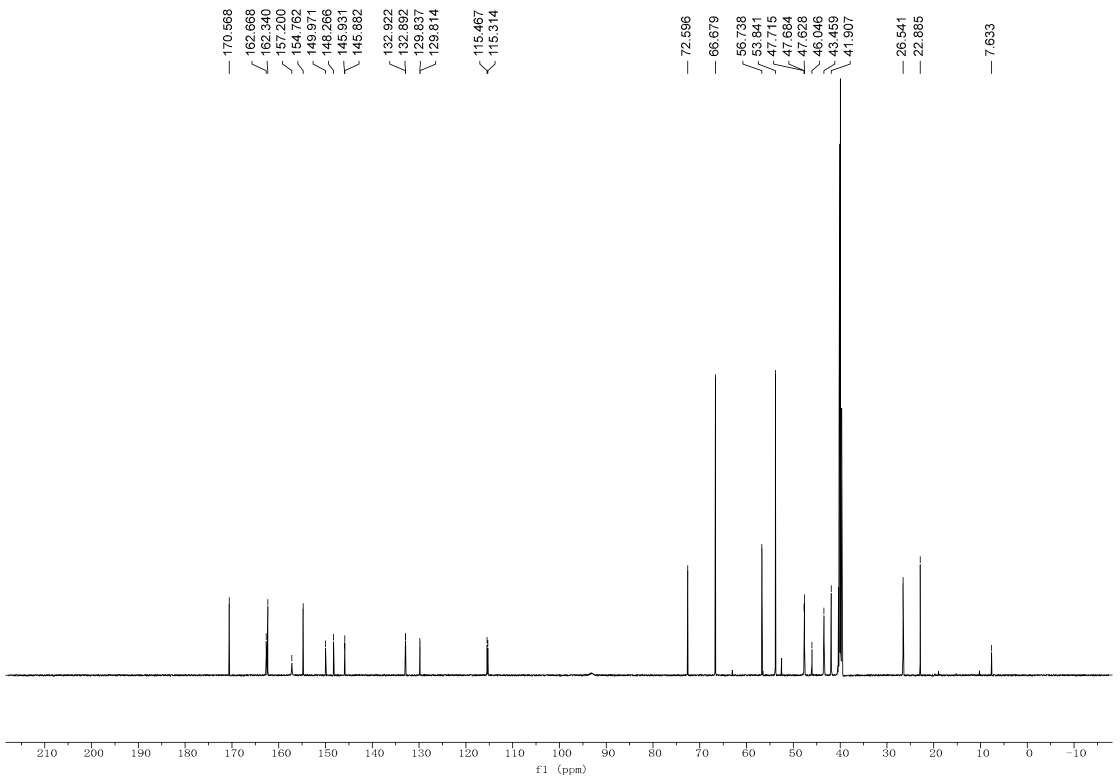

Figure S26. ^{13}C NMR Spectrum (DMSO-d_6, 150 MHz) of **6h**.

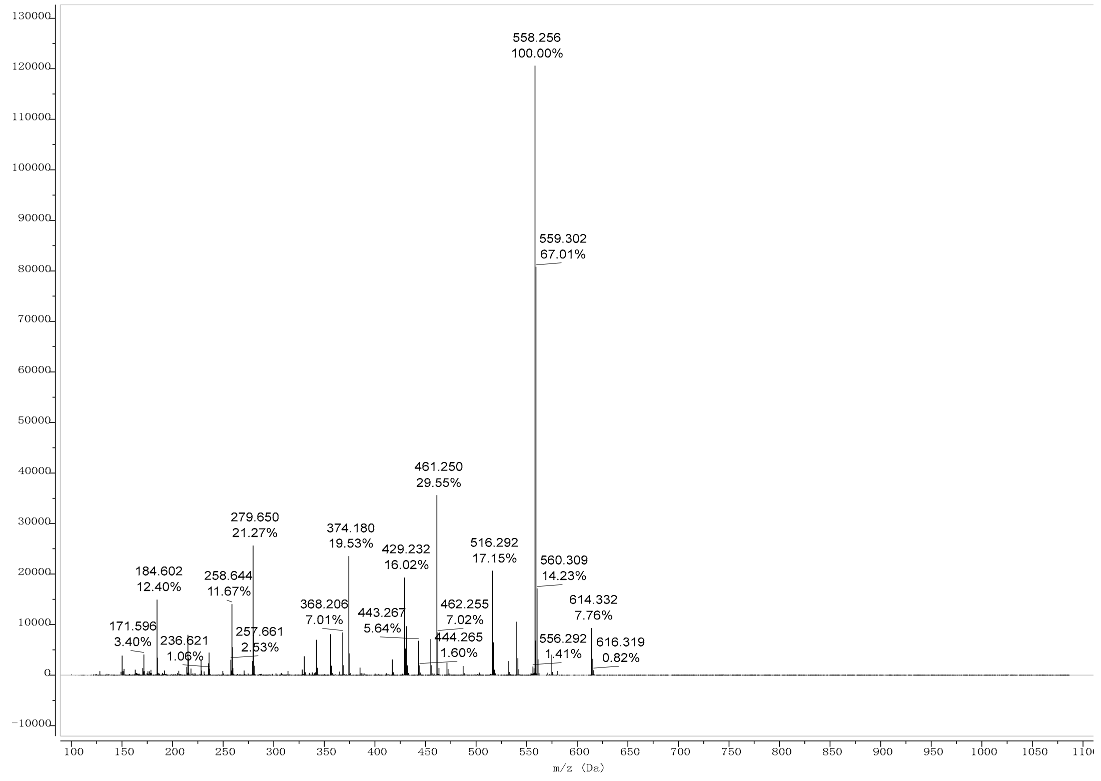

Figure S27. MS for $C_{26}H_{35}FN_8O_4$ (Mwt.: 557.63): m/z 558.256 ([M+H] $^+$, bp) of **6h**.

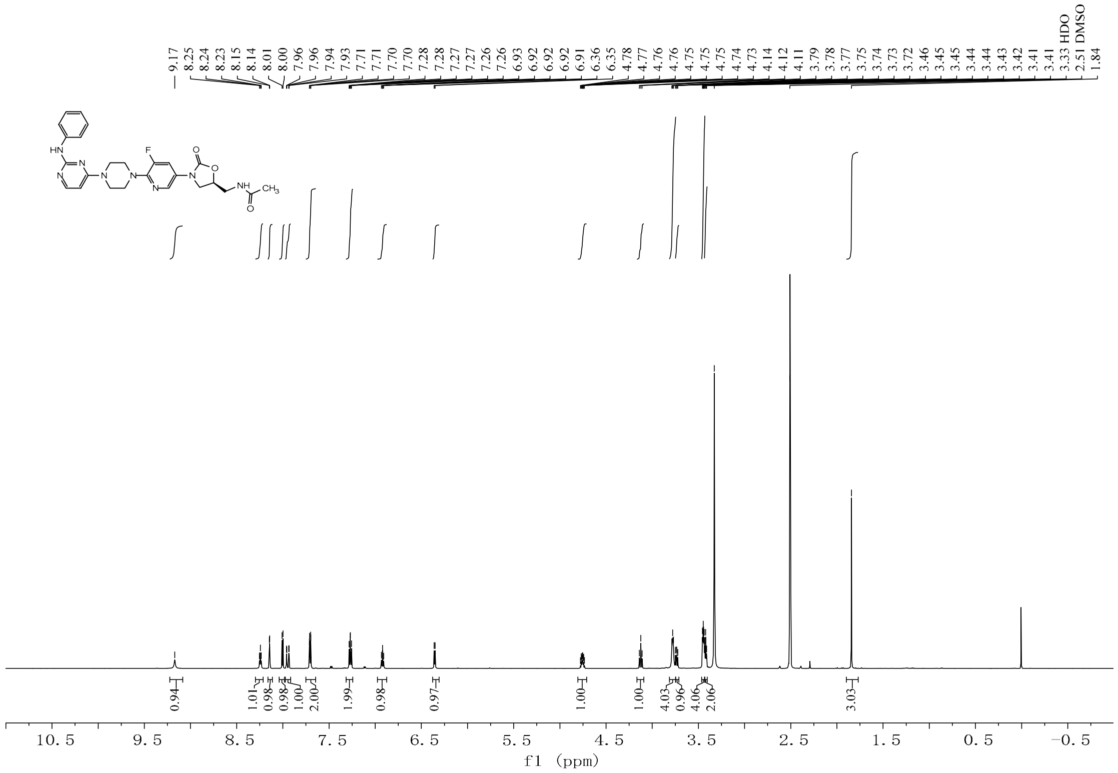

Figure S28. ^{1}H NMR Spectrum (DMSO-d_6, 600 MHz) of **6i**.

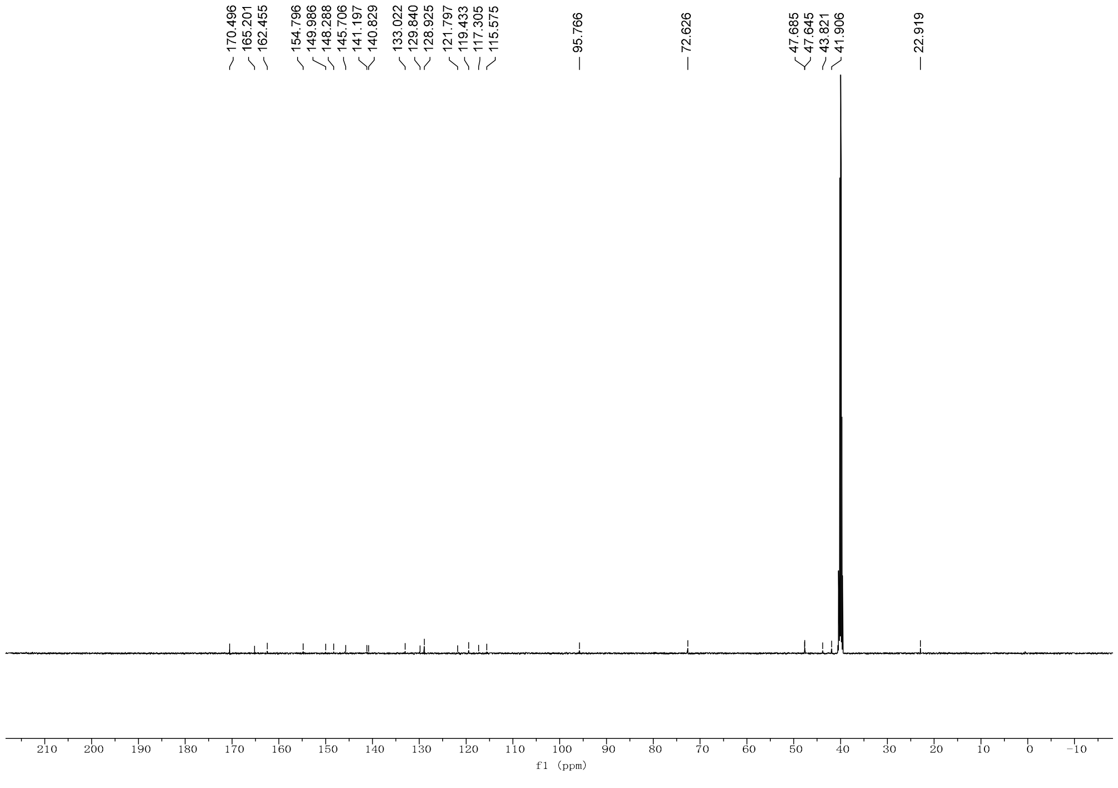

Figure S29. ^{13}C NMR Spectrum (DMSO-d_6, 150 MHz) of **6i**.

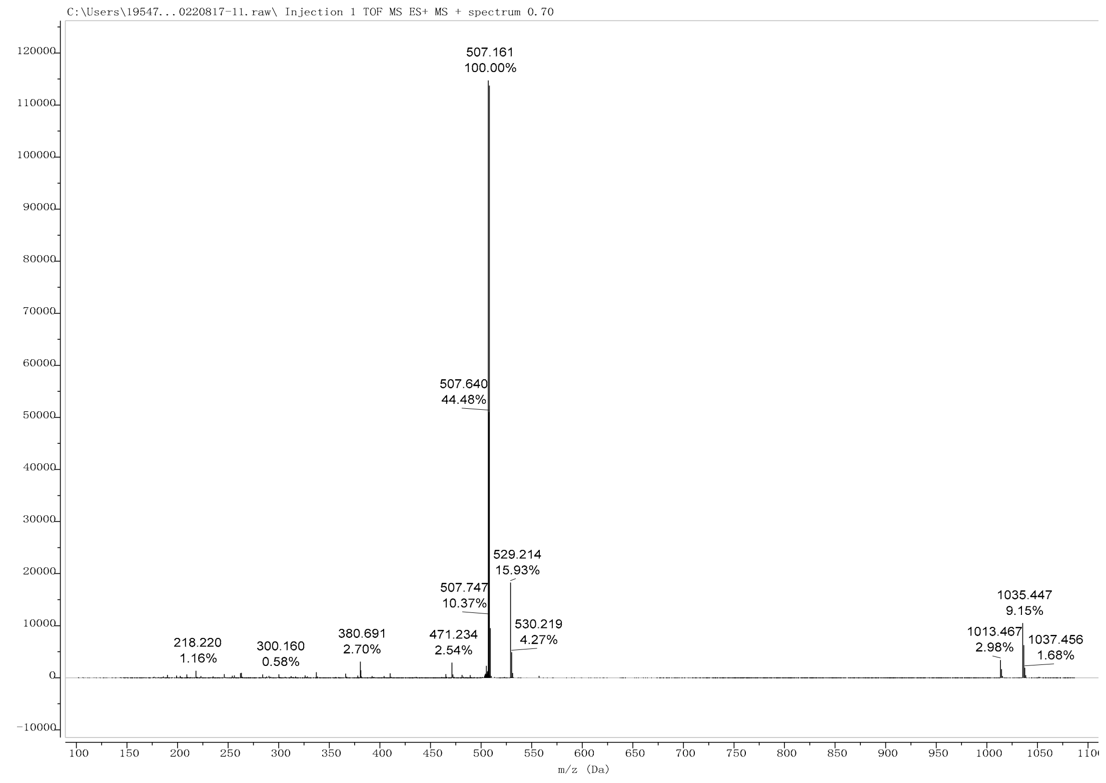

Figure S30. MS for $C_{25}H_{27}FN_8O_3$ (Mwt.: 506.54): m/z 507.161 ([M+H] $^+$, bp) of **6i**.

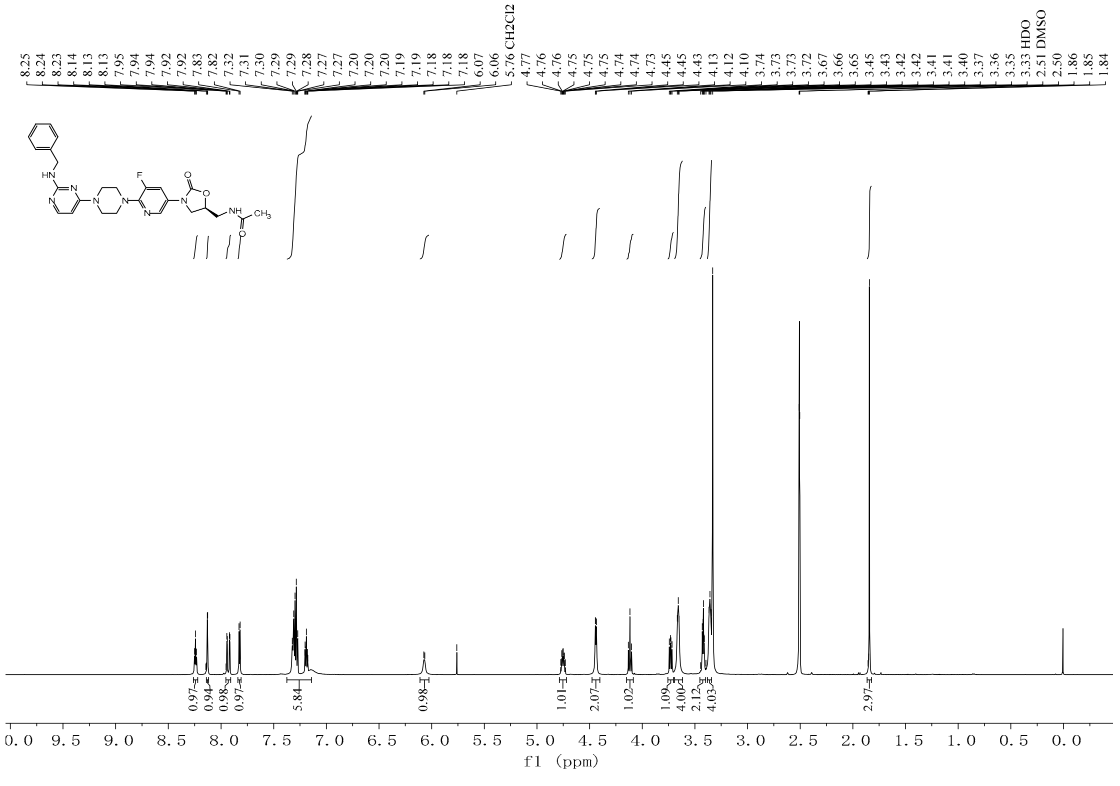

Figure S31. ^{1}H NMR Spectrum (DMSO-d_6, 600 MHz) of **6j**.

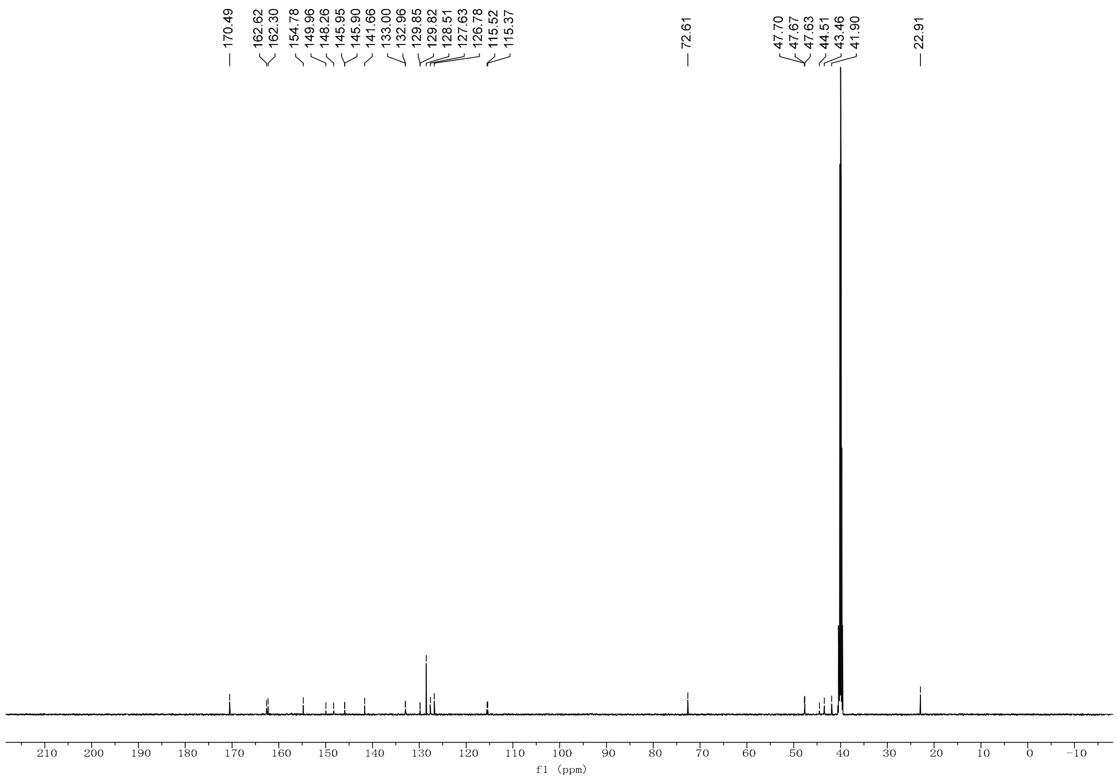

Figure S32. ^{13}C NMR Spectrum (DMSO-d_6, 150 MHz) of **6j**.

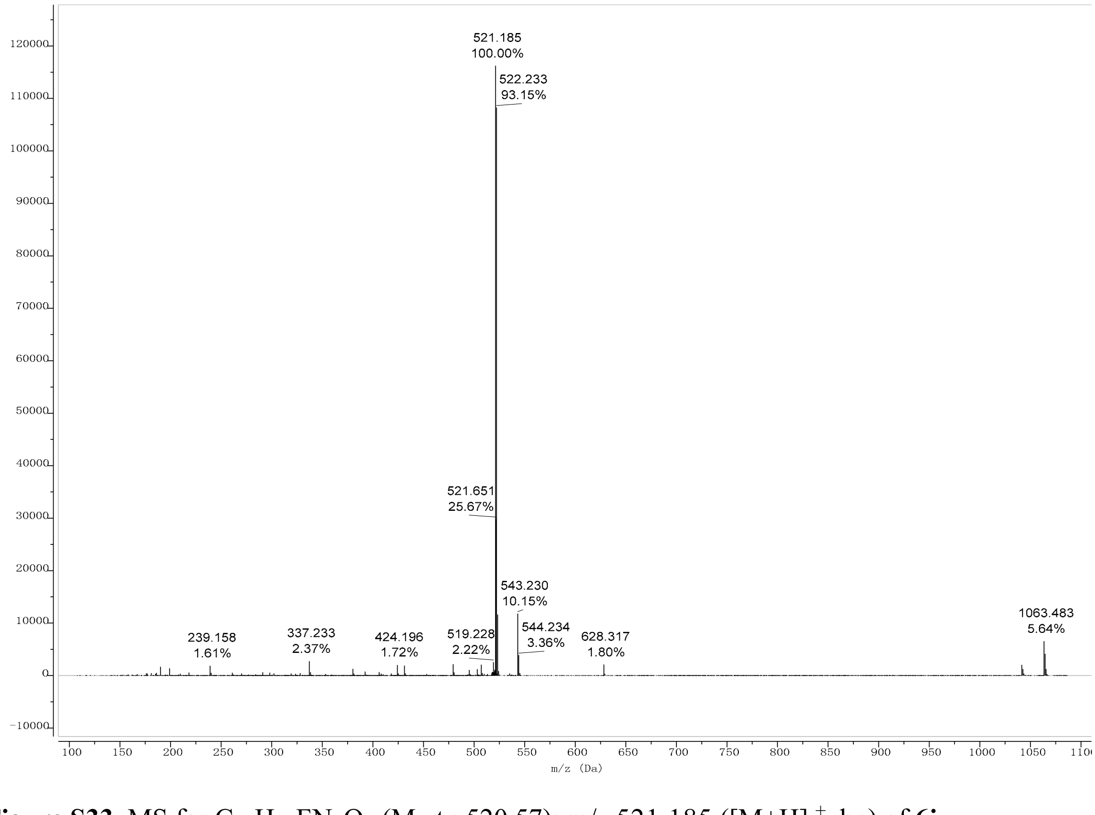

Figure S33. MS for $C_{26}H_{29}FN_8O_3$ (Mwt.: 520.57): m/z 521.185 ([M+H] $^+$, bp) of **6j**.

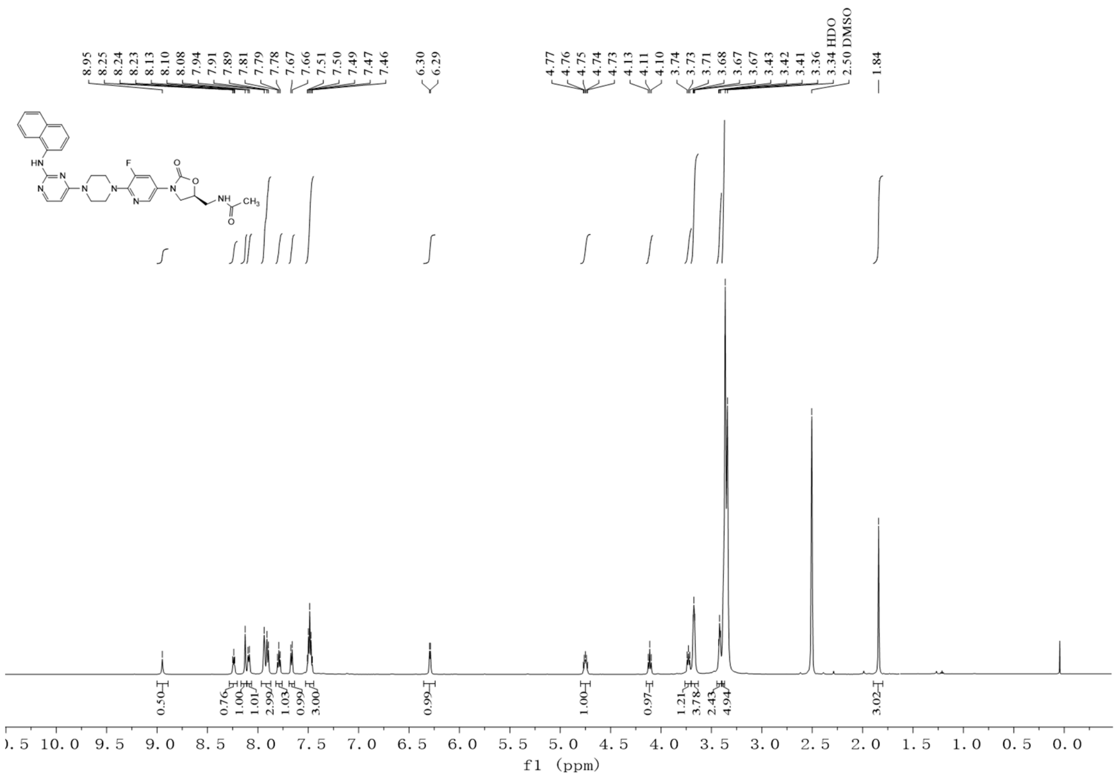

Figure S34. ^{1}H NMR Spectrum (DMSO-d_6, 600 MHz) of **6k**.

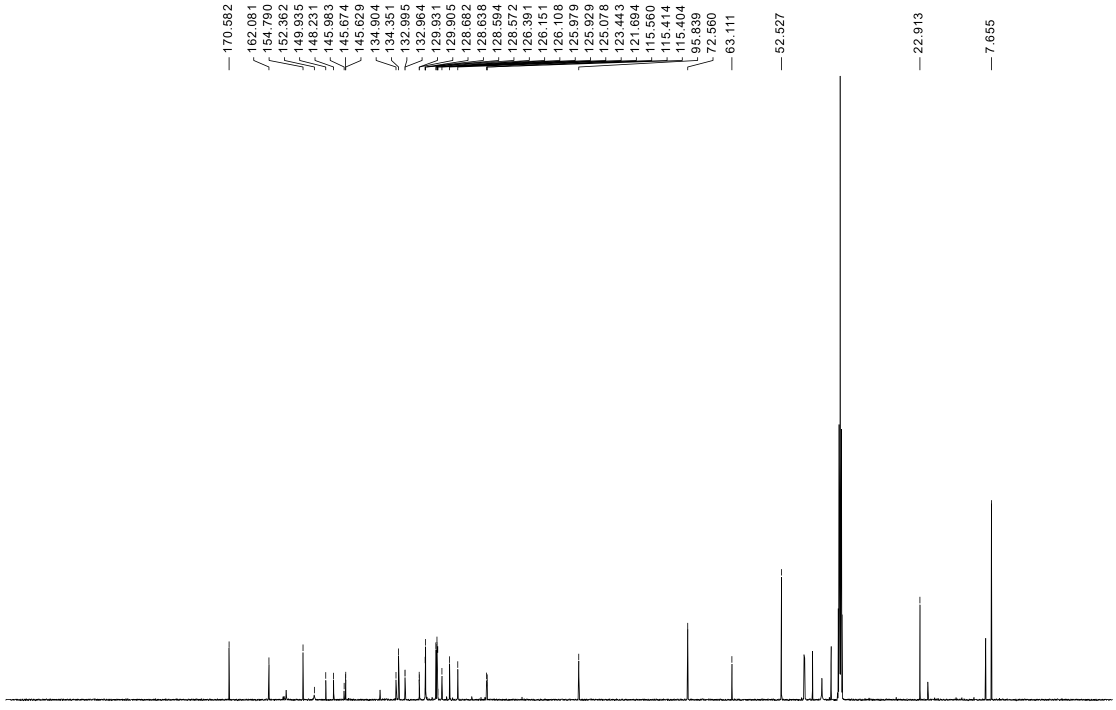

Figure S35. ^{13}C NMR Spectrum (DMSO-d_6, 150 MHz) of **6k**.

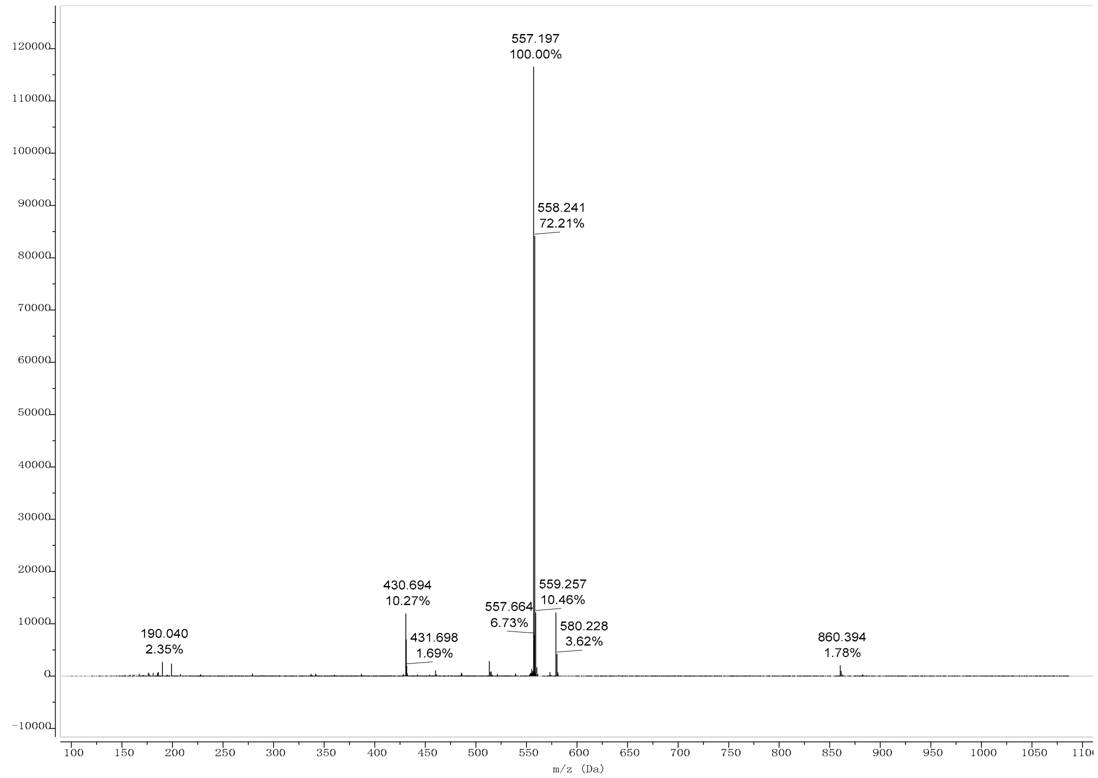

Figure S36. MS for $C_{29}H_{29}FN_8O_3$ (Mwt.: 556.60): m/z 557.197 ([M+H] $^{+}$, bp) of **6k**.

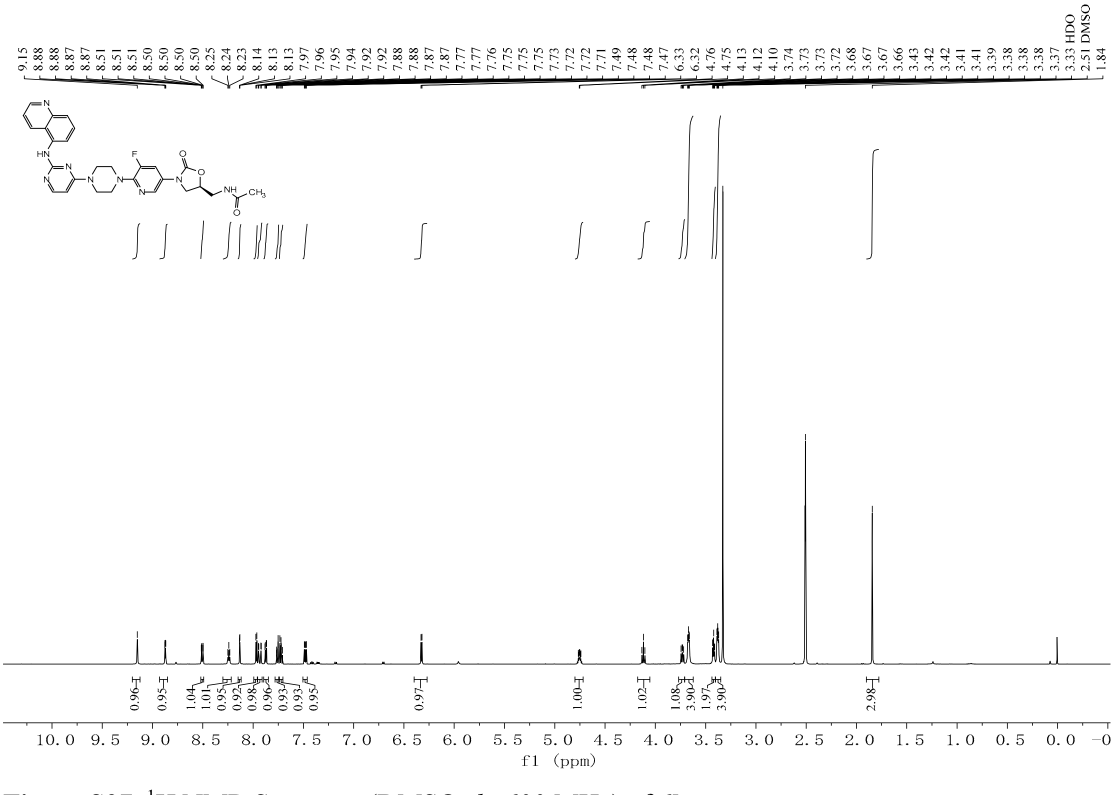

Figure S37. ^{1}H NMR Spectrum (DMSO-d_6, 600 MHz) of **6l**.

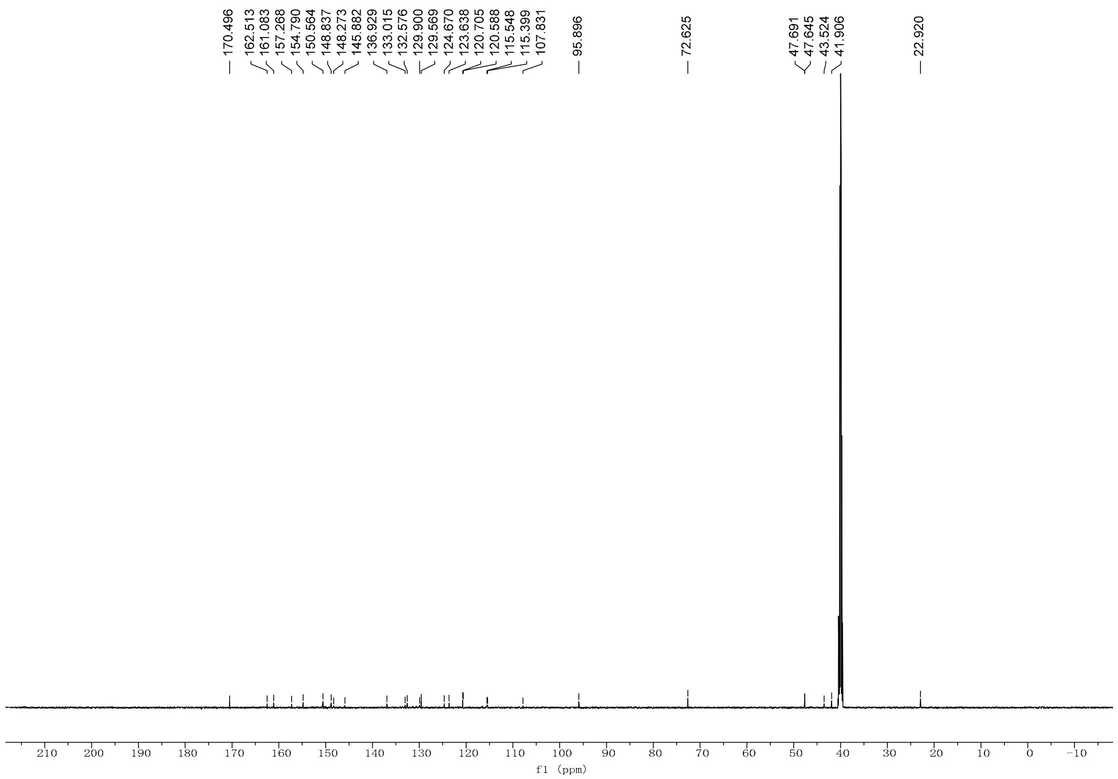

Figure S38. ^{13}C NMR Spectrum (DMSO-d_6, 150 MHz) of **6l**.

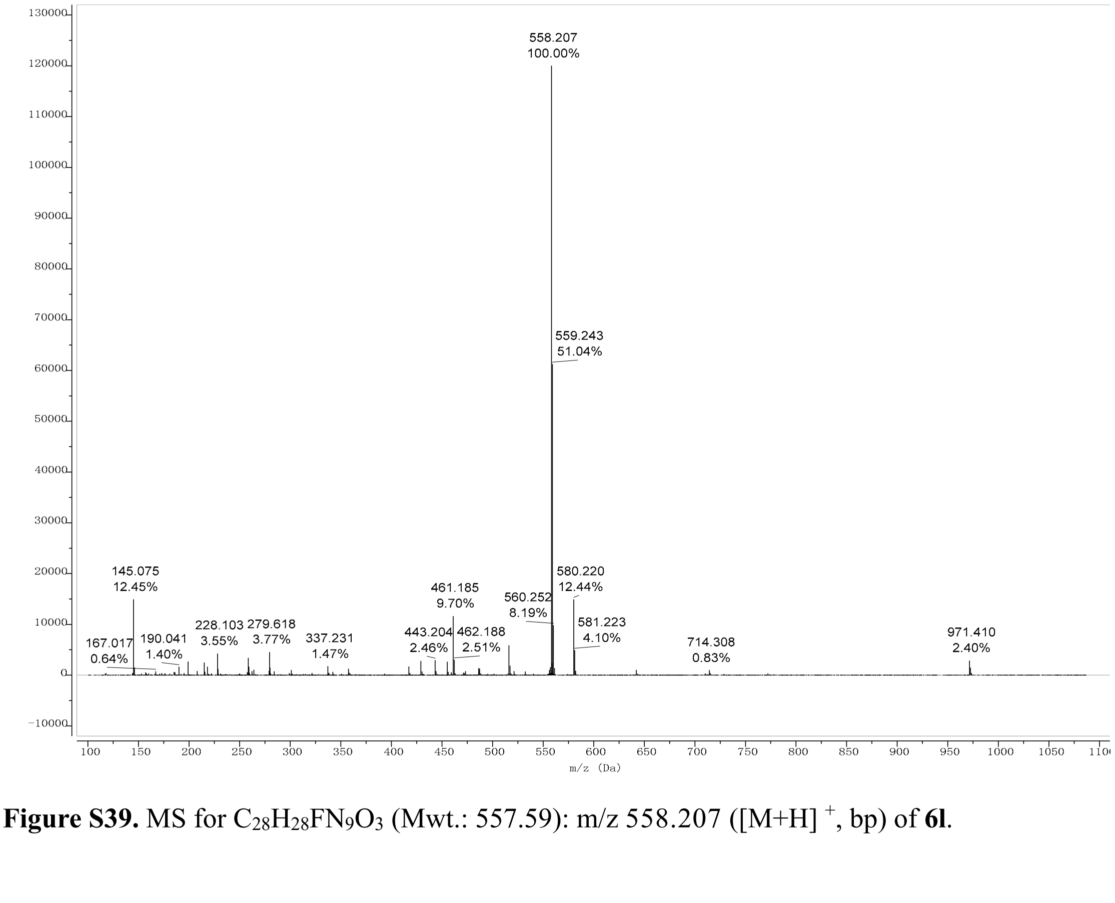

Figure S39. MS for $C_{28}H_{28}FN_9O_3$ (Mwt.: 557.59): m/z 558.207 ([M+H] $^+$, bp) of **6l**.

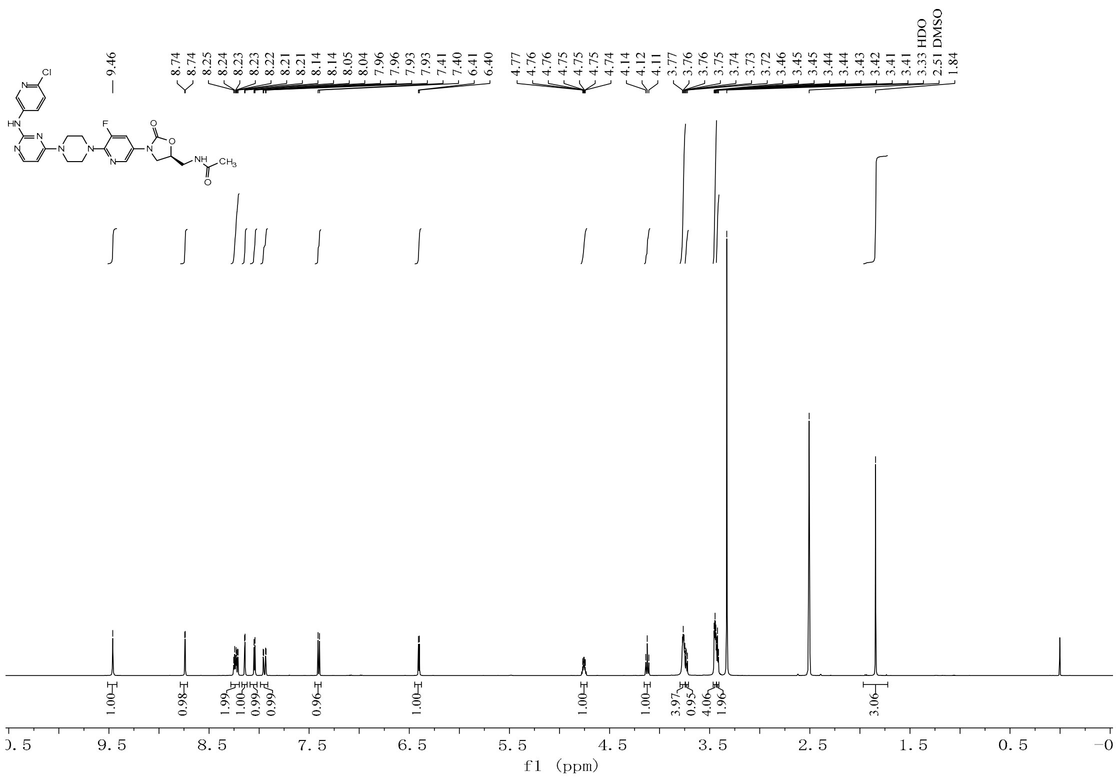

Figure S40. ^{1}H NMR Spectrum (DMSO- d_6, 600 MHz) of **6m**.

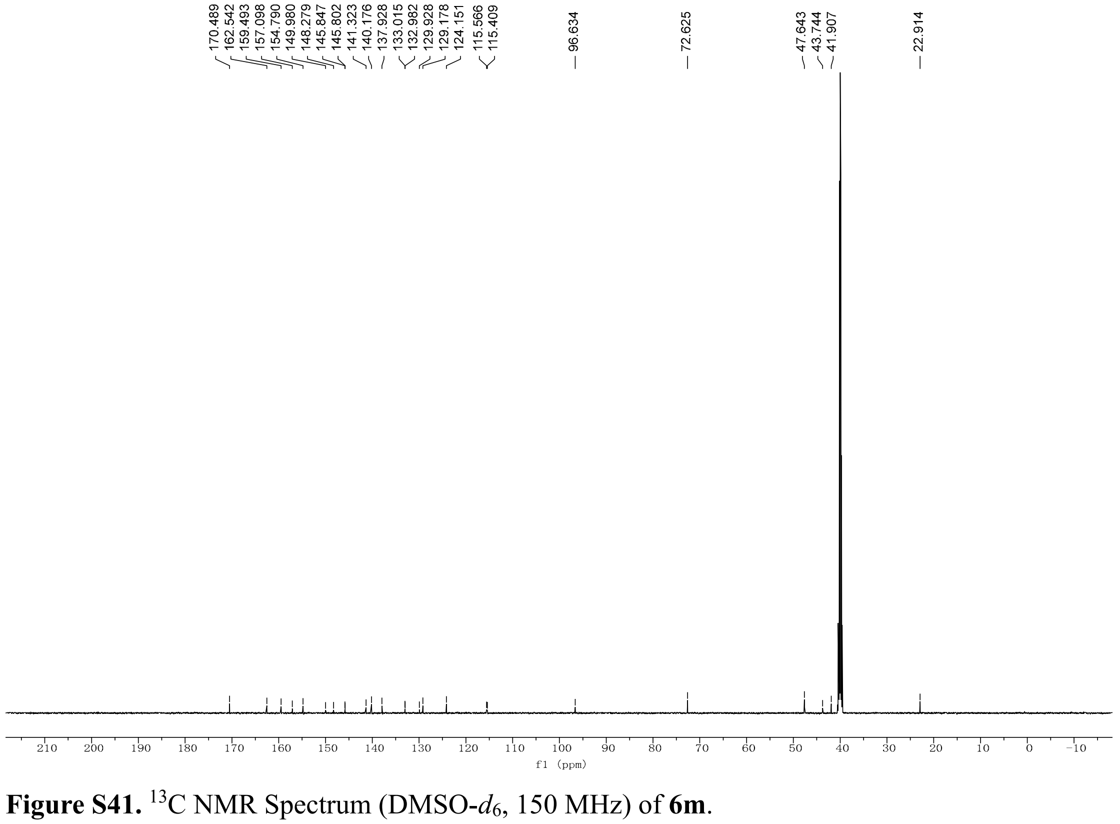

Figure S41. ^{13}C NMR Spectrum (DMSO-d_6, 150 MHz) of **6m**.

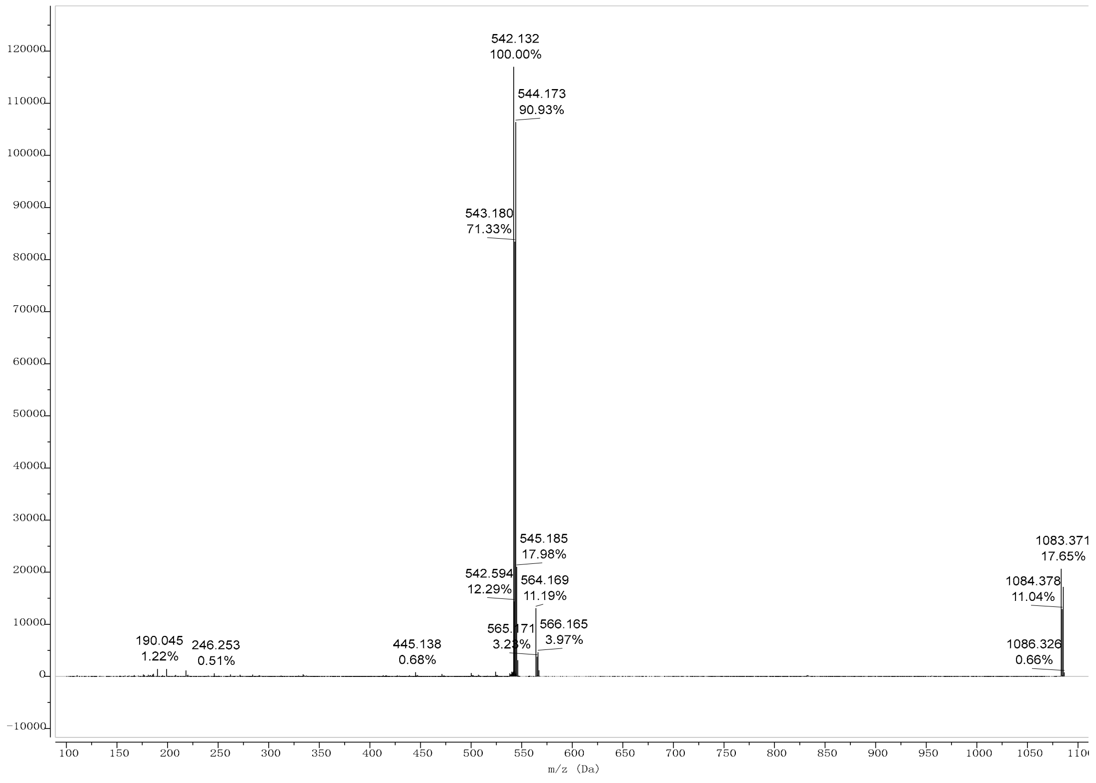

Figure S42. MS for $C_{24}H_{25}ClFN_9O_3$ (Mwt.: 541.97): m/z 542.132 ([M+H] $^+$, bp) of **6m**.

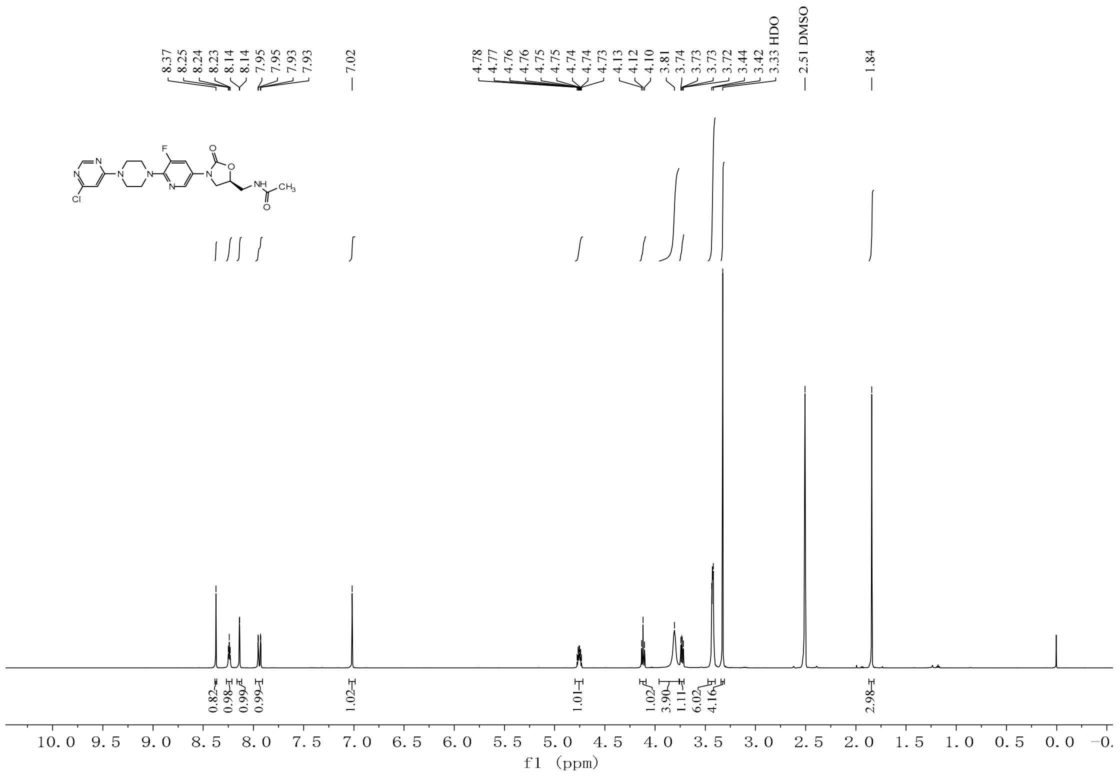

Figure S43. ^{1}H NMR Spectrum (DMSO-d_6, 600 MHz) of **7a**.

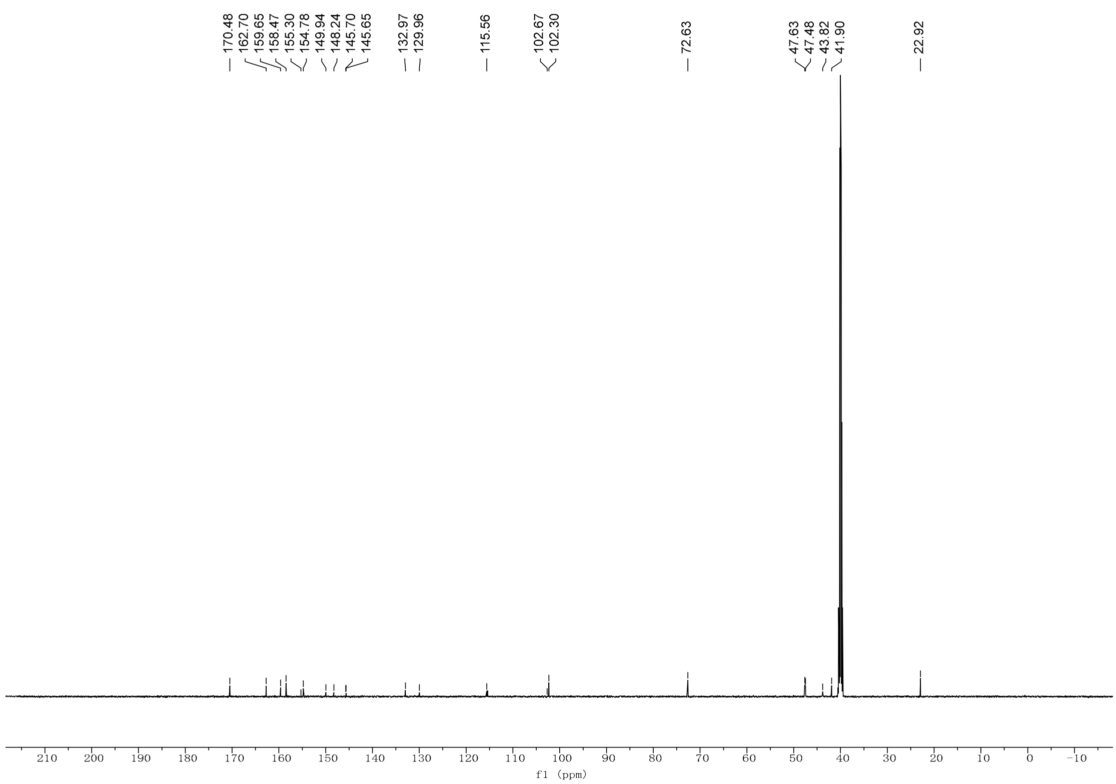

Figure S44. ^{13}C NMR Spectrum (DMSO-d_6, 150 MHz) of **7a**.

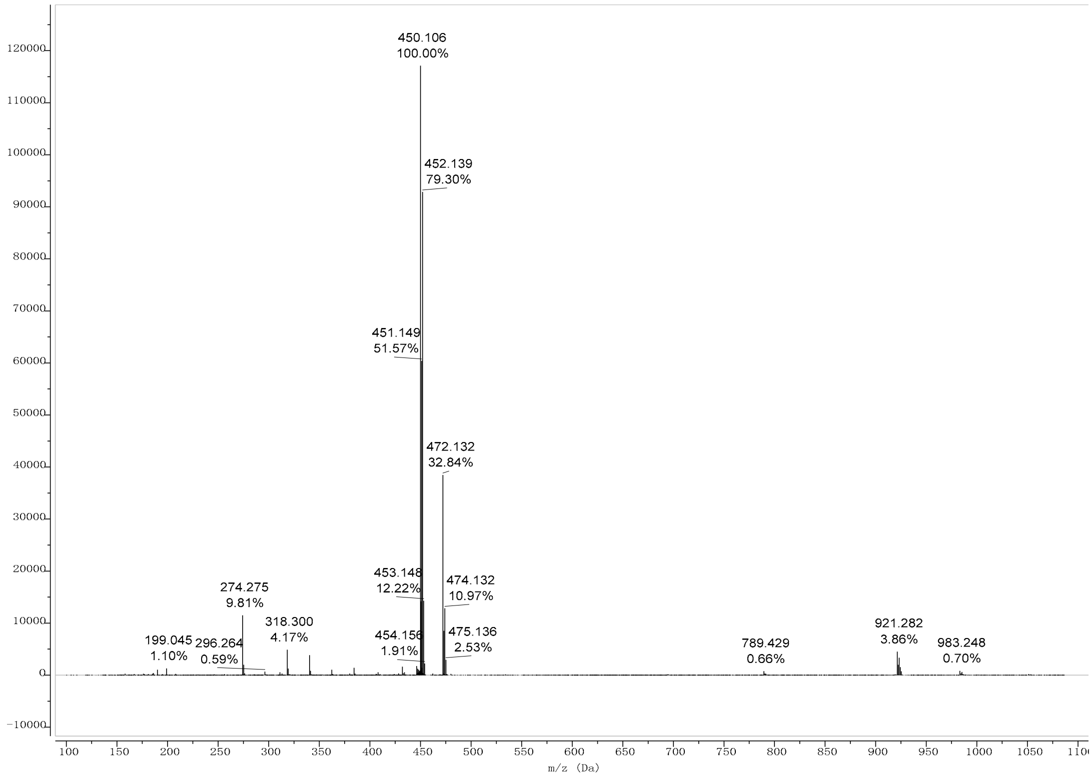

Figure S45. MS for $C_{19}H_{21}ClFN_7O_3$ (Mwt.: 449.87): m/z 450.106 ([M+H] $^+$, bp) of **7a**.

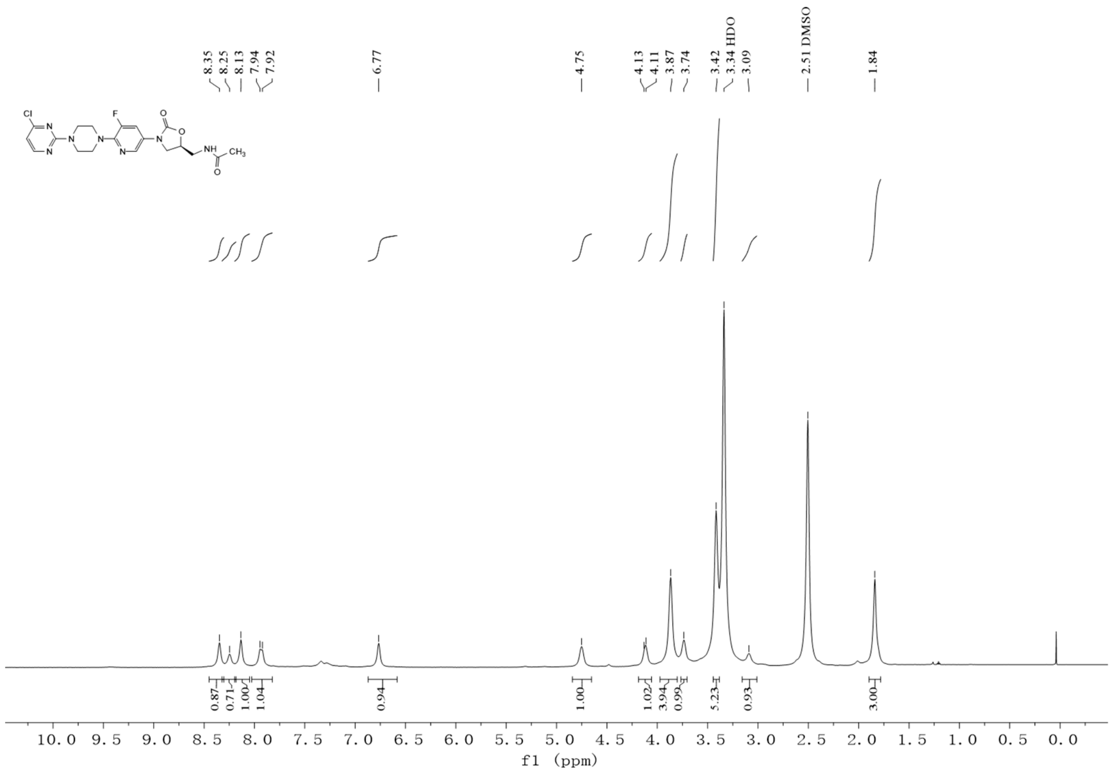

Figure S46. ^{1}H NMR Spectrum (DMSO-d_6, 600 MHz) of **7b**.

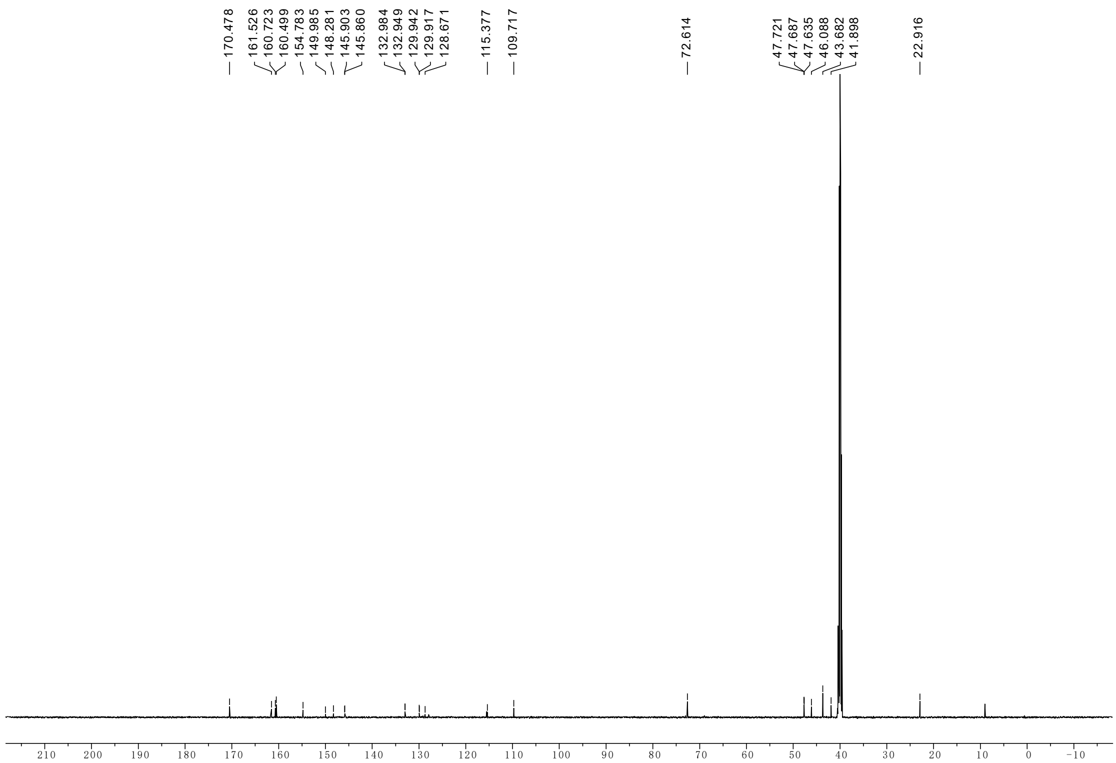

Figure S47. ^{13}C NMR Spectrum (DMSO-d_6, 150 MHz) of **7b**.

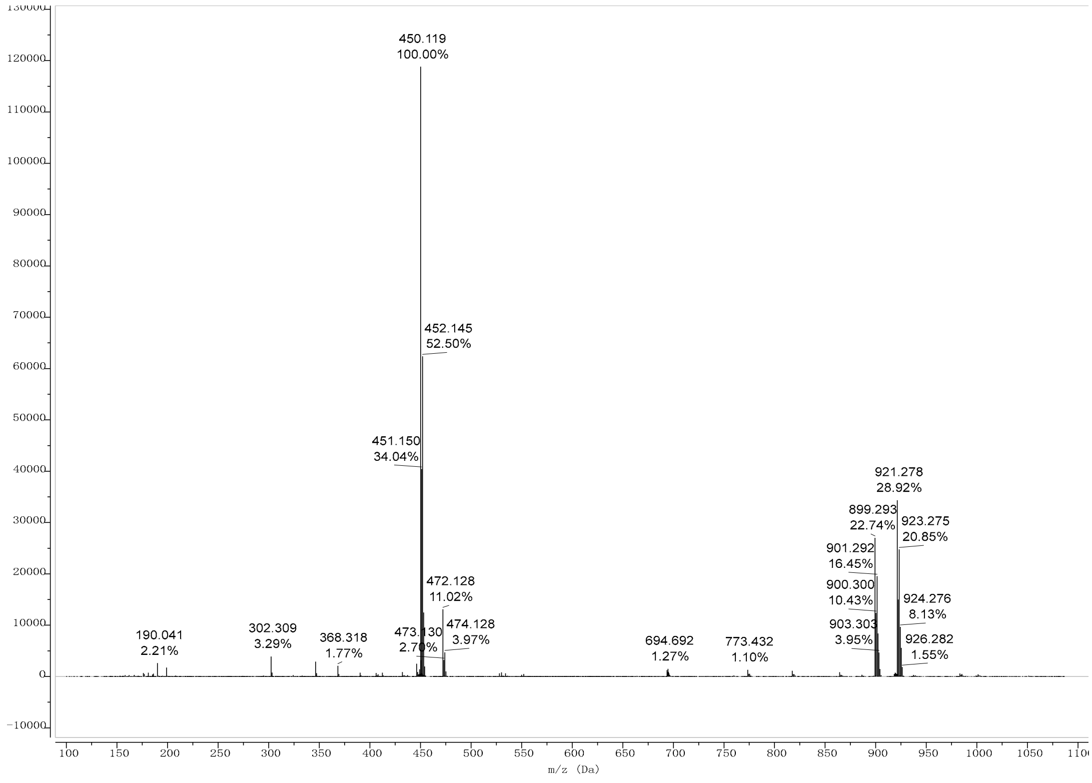

Figure S48. MS for $C_{19}H_{21}ClFN_7O_3$ (Mwt.: 449.87): m/z 450.119 ([M+H] $^{+}$, bp) of **7b**.

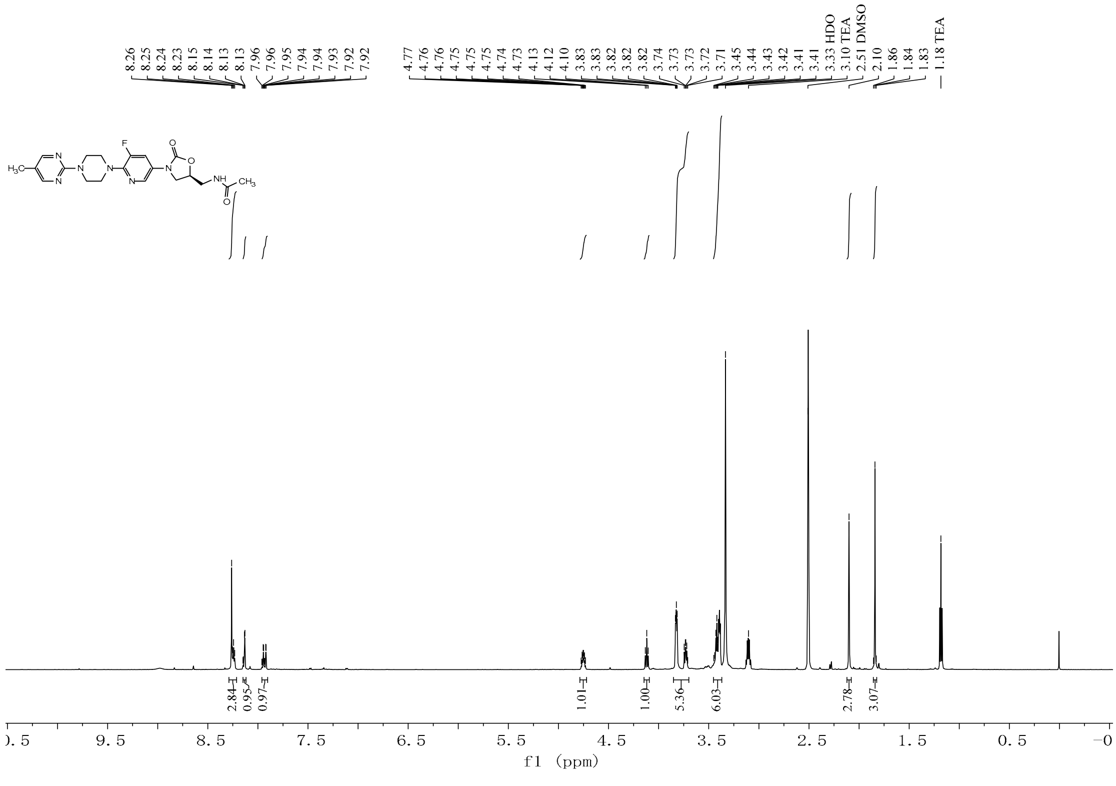

Figure S49. ^{1}H NMR Spectrum (DMSO-d_6, 600 MHz) of **7c**.

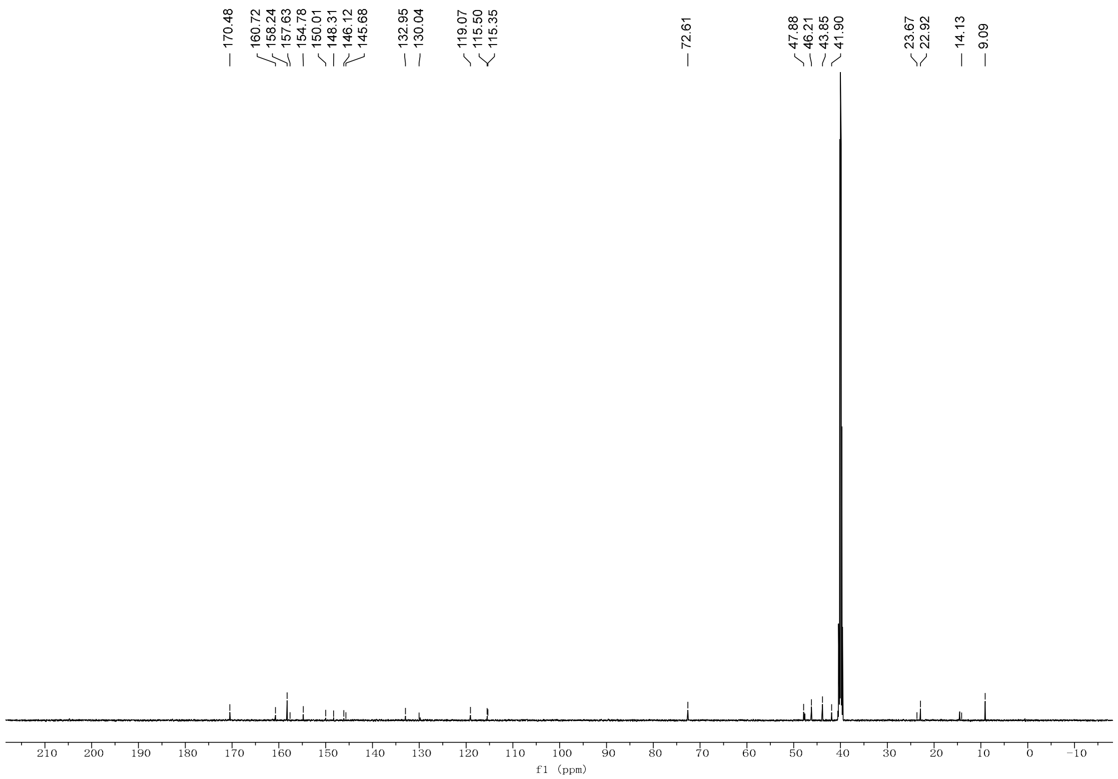

Figure S50. ^{13}C NMR Spectrum (DMSO-d_6, 150 MHz) of **7c**.

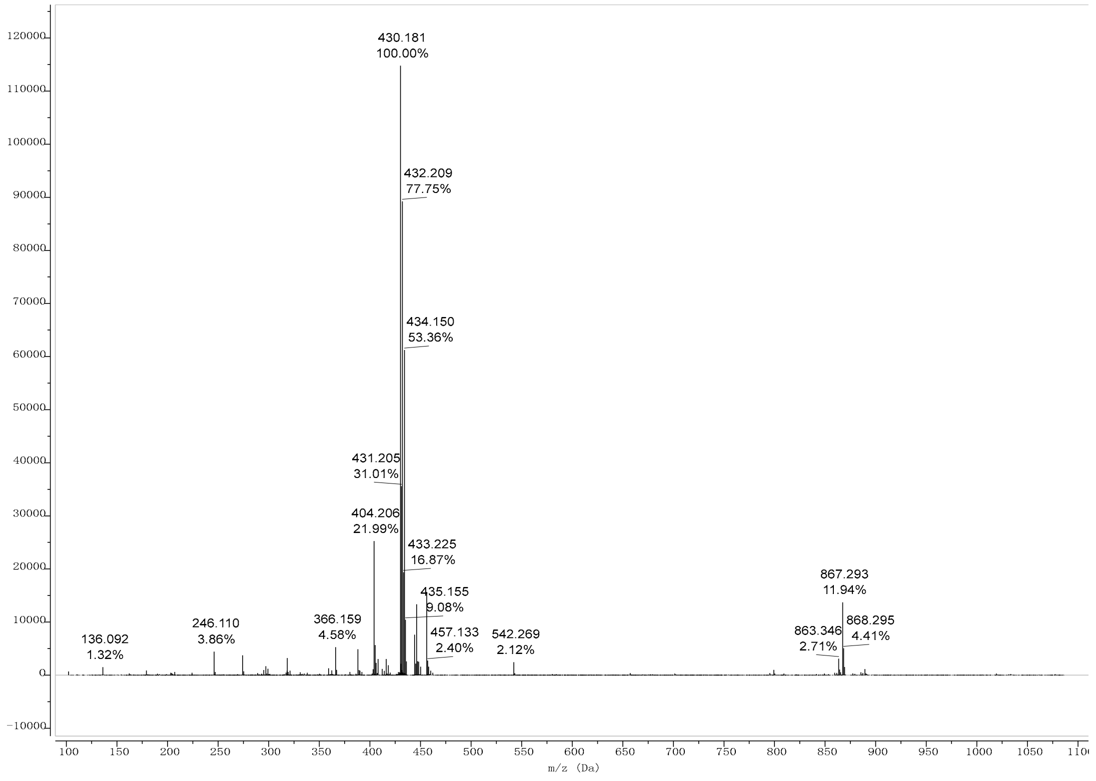

Figure S51. MS for $C_{20}H_{24}FN_7O_3$ (Mwt.: 429.46): m/z 430.181 ($[M+H]^+$, bp) of **7c**.

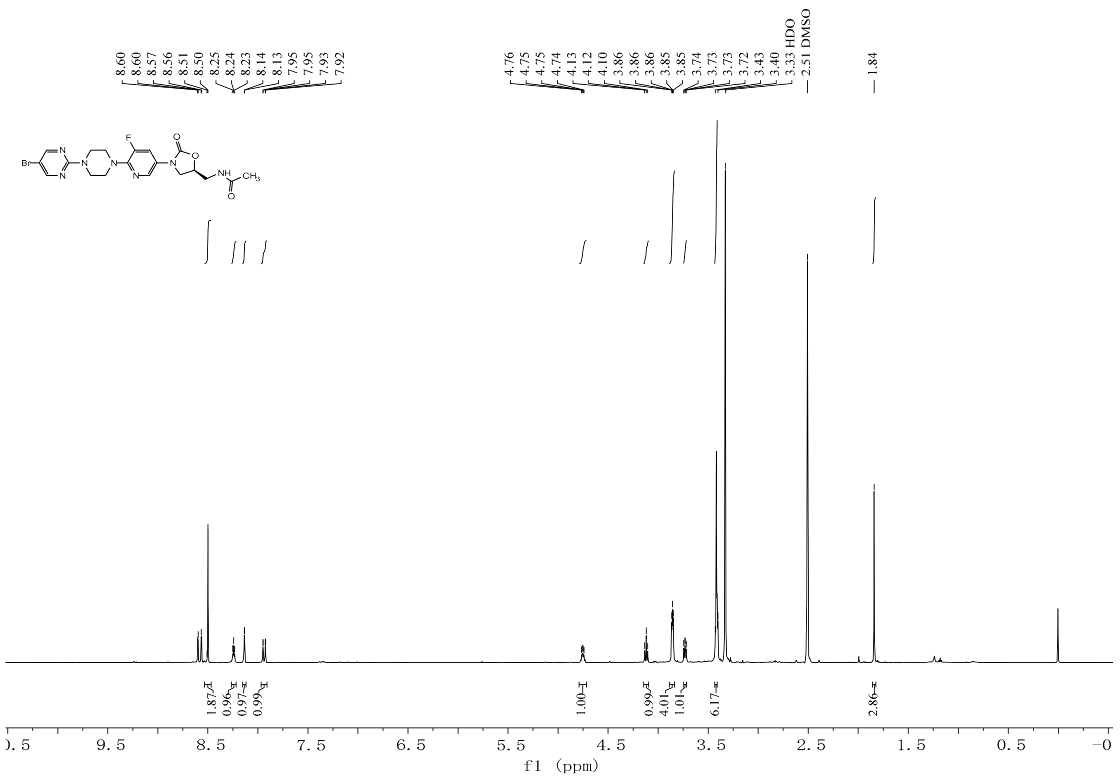

Figure S52. ^{1}H NMR Spectrum (DMSO-d_6, 600 MHz) of **7d**.

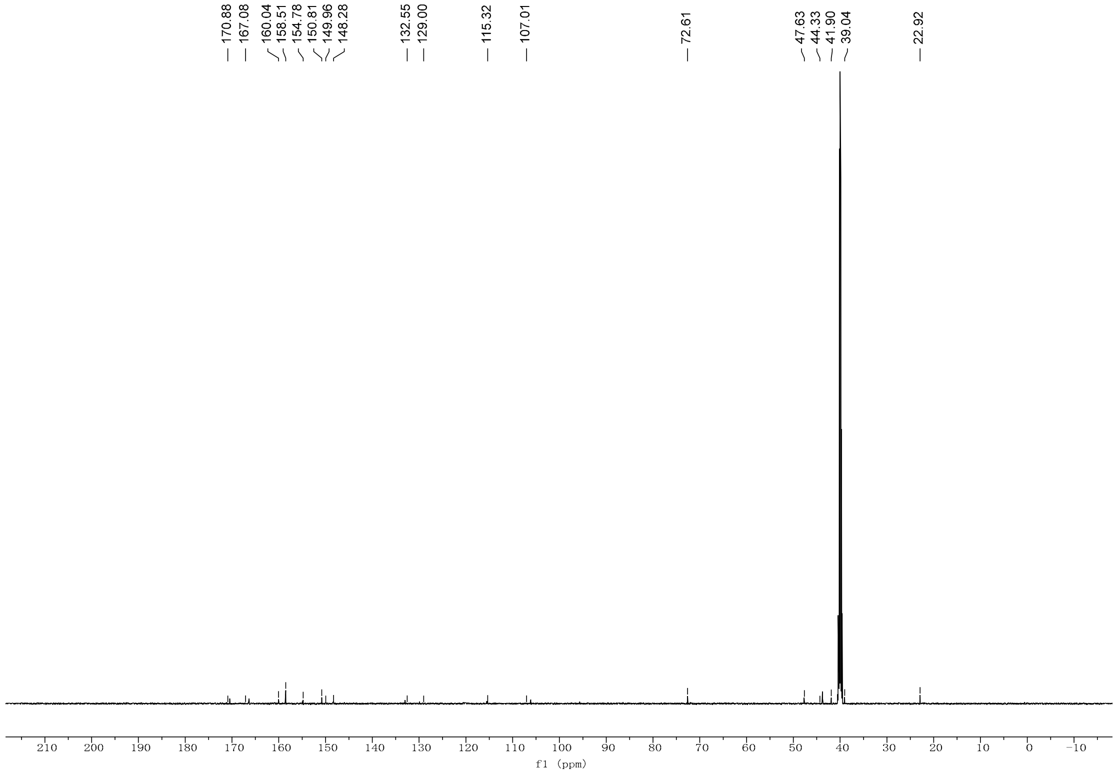

Figure S53. ^{13}C NMR Spectrum (DMSO-d_6, 150 MHz) of **7d**.

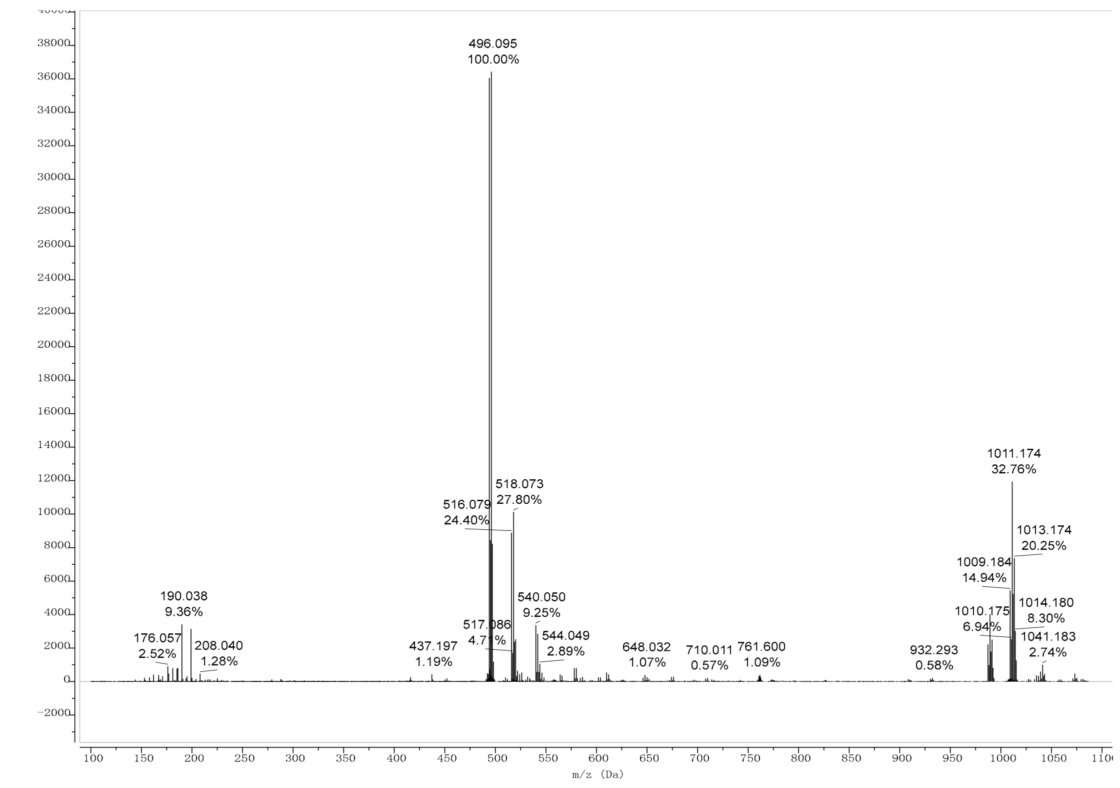

Figure S54. MS for $C_{19}H_{21}BrFN_7O_3$ (Mwt.: 494.33): m/z 496.095 ([M+H]$^+$, bp) of **7d**.

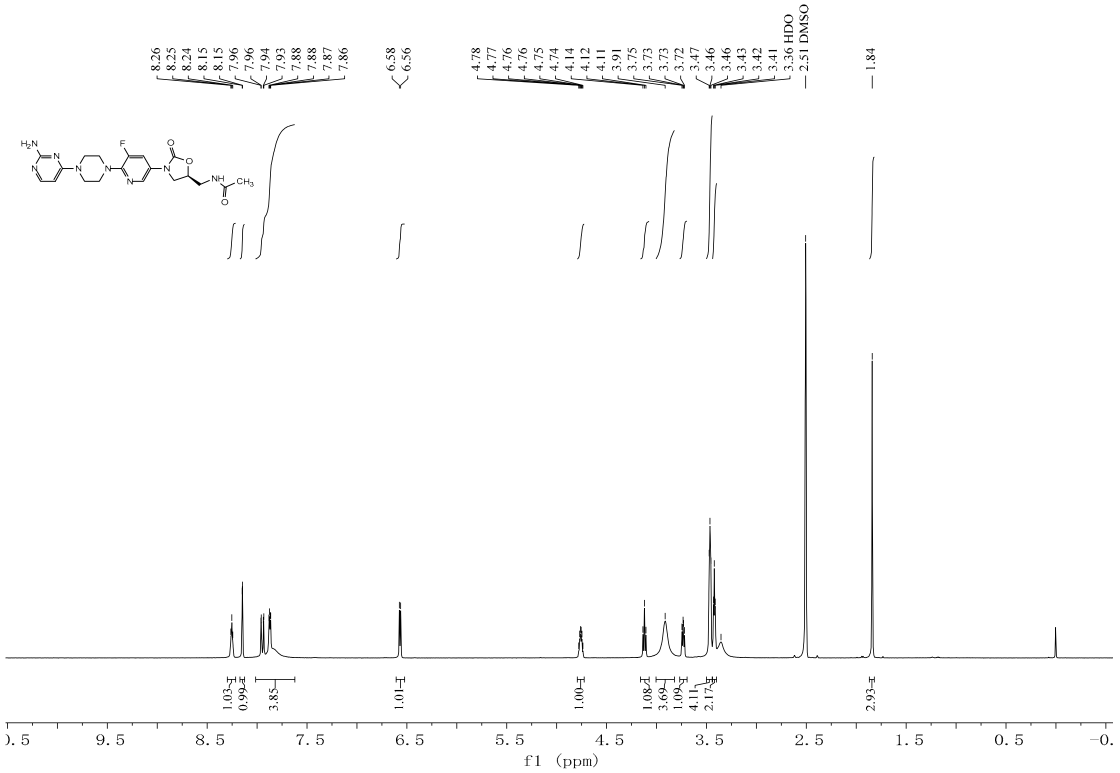

Figure S55. ^{1}H NMR Spectrum (DMSO-d_6, 600 MHz) of **7e**.

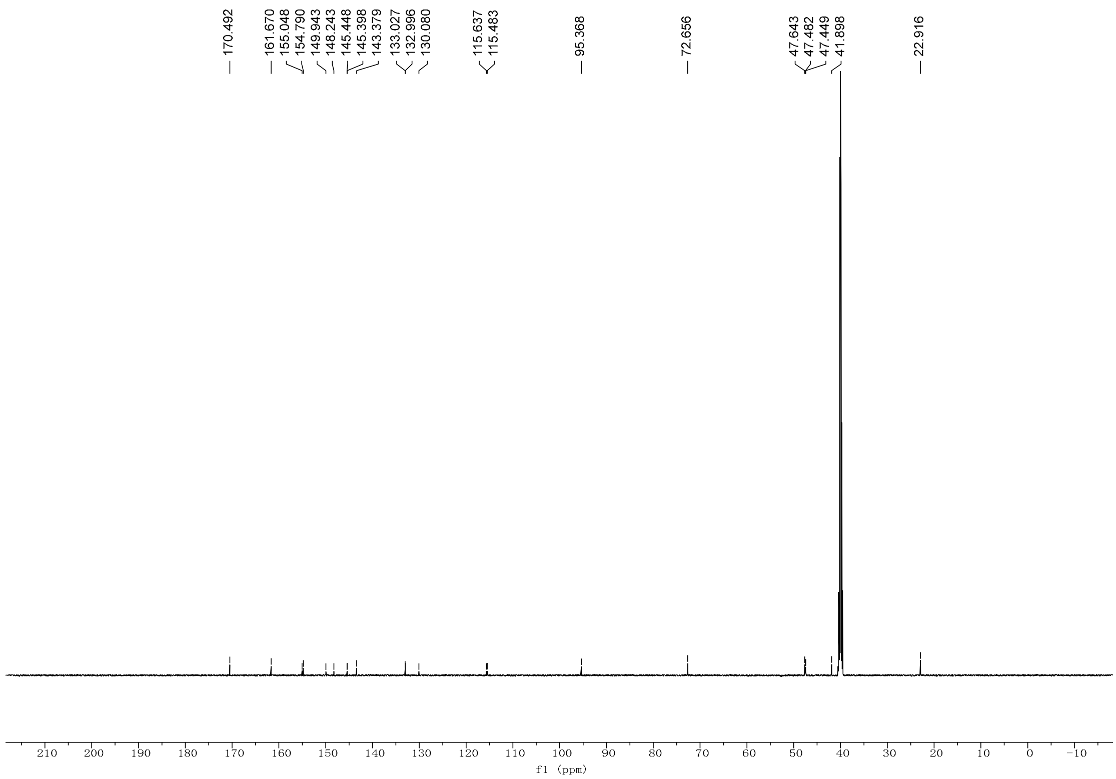

Figure S56. ^{13}C NMR Spectrum (DMSO-d_6, 150 MHz) of **7e**.

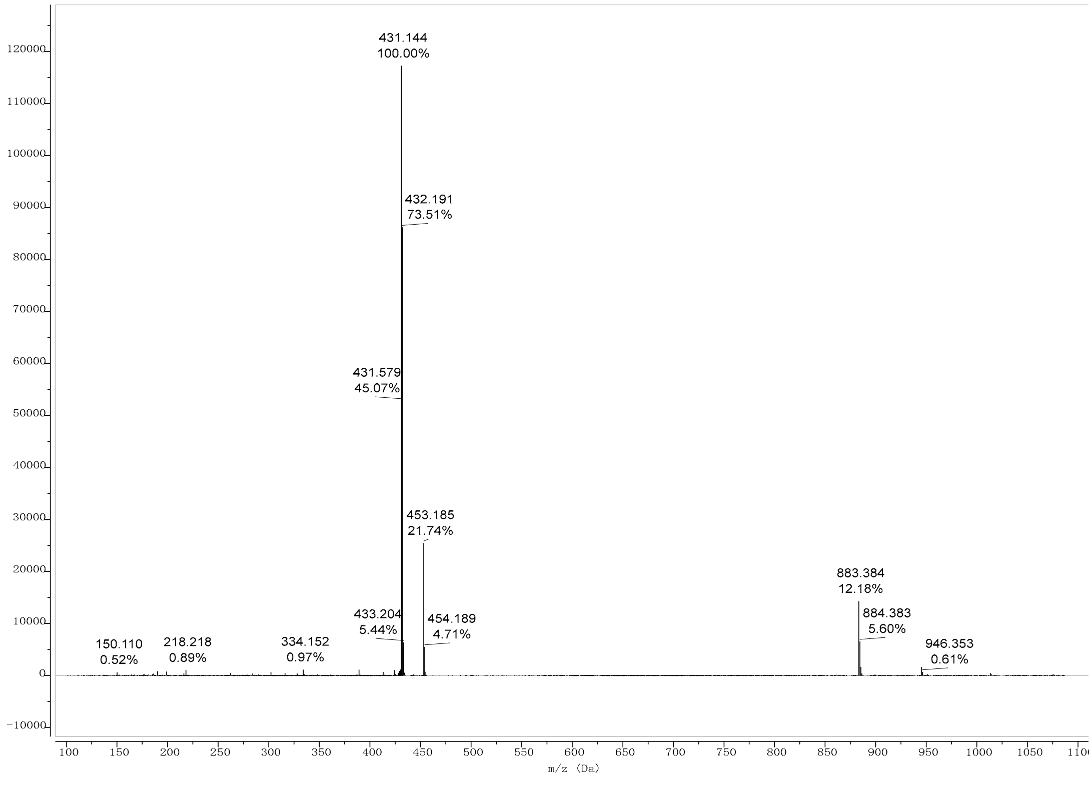

Figure S57. MS for $C_{19}H_{23}FN_8O_3$ (Mwt.: 430.44): m/z 431.144 ([M+H]$^+$, bp) of **7e**.

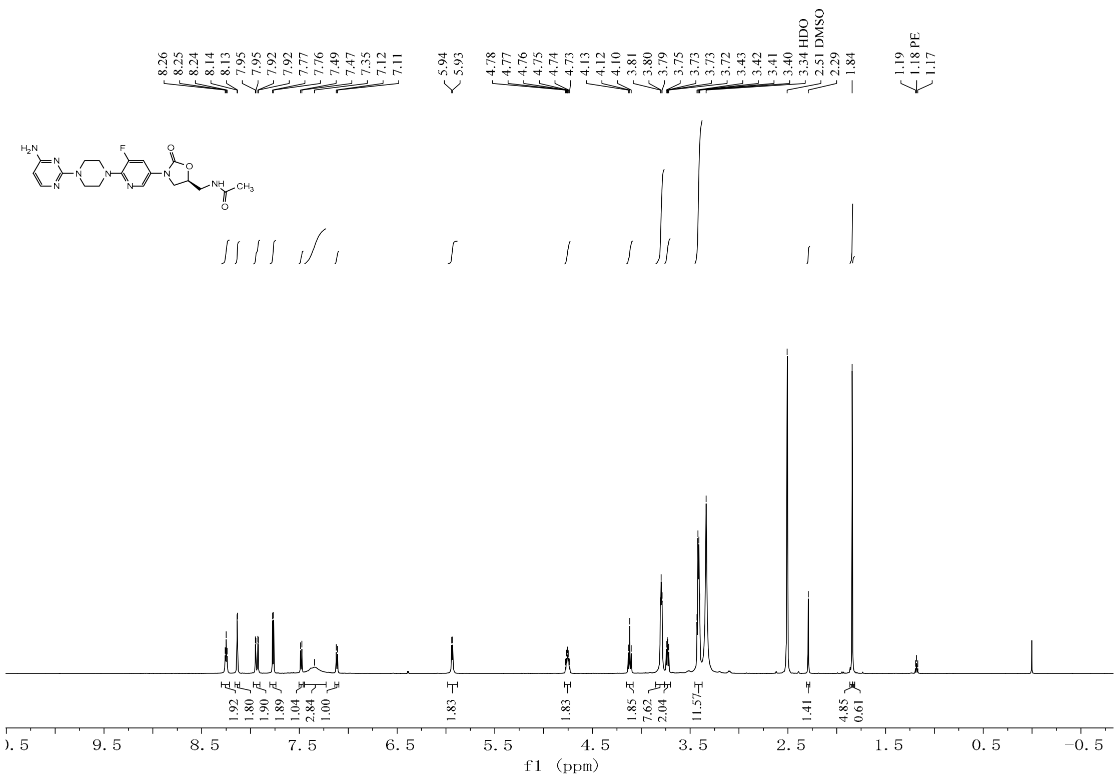

Figure S58. ^{1}H NMR Spectrum (DMSO-d_6, 600 MHz) of **7f**.

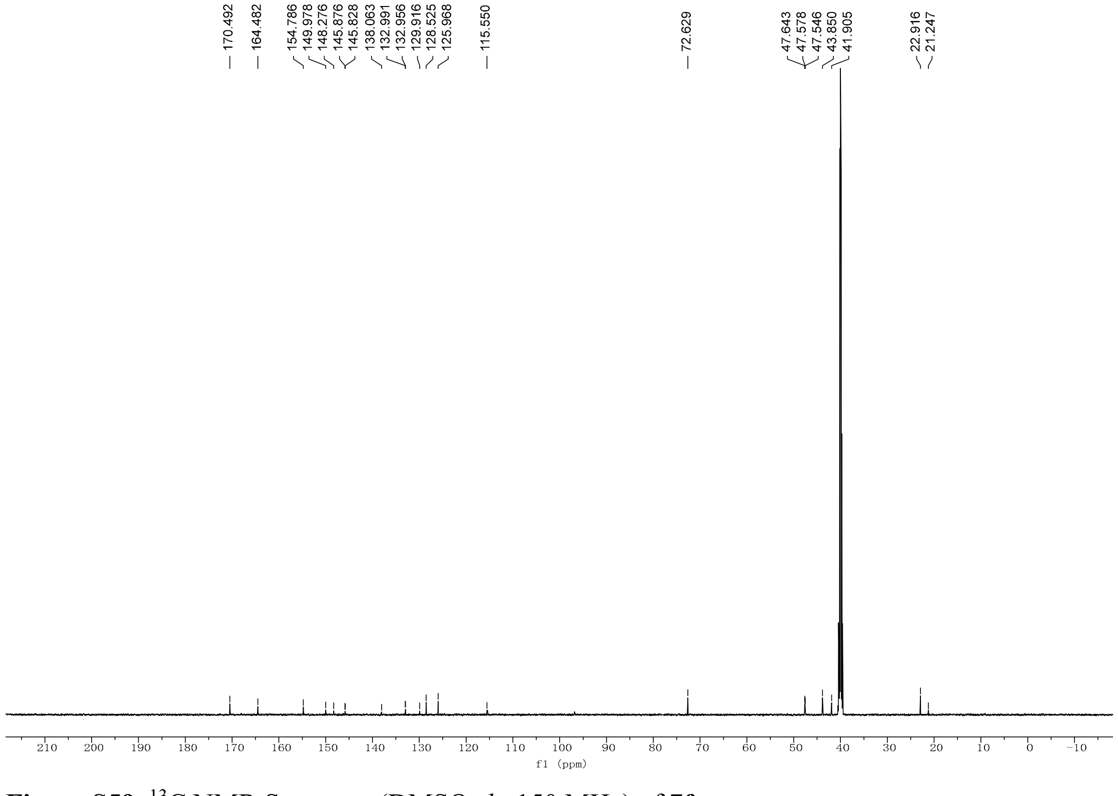

Figure S59. ^{13}C NMR Spectrum (DMSO-d_6, 150 MHz) of **7f**.

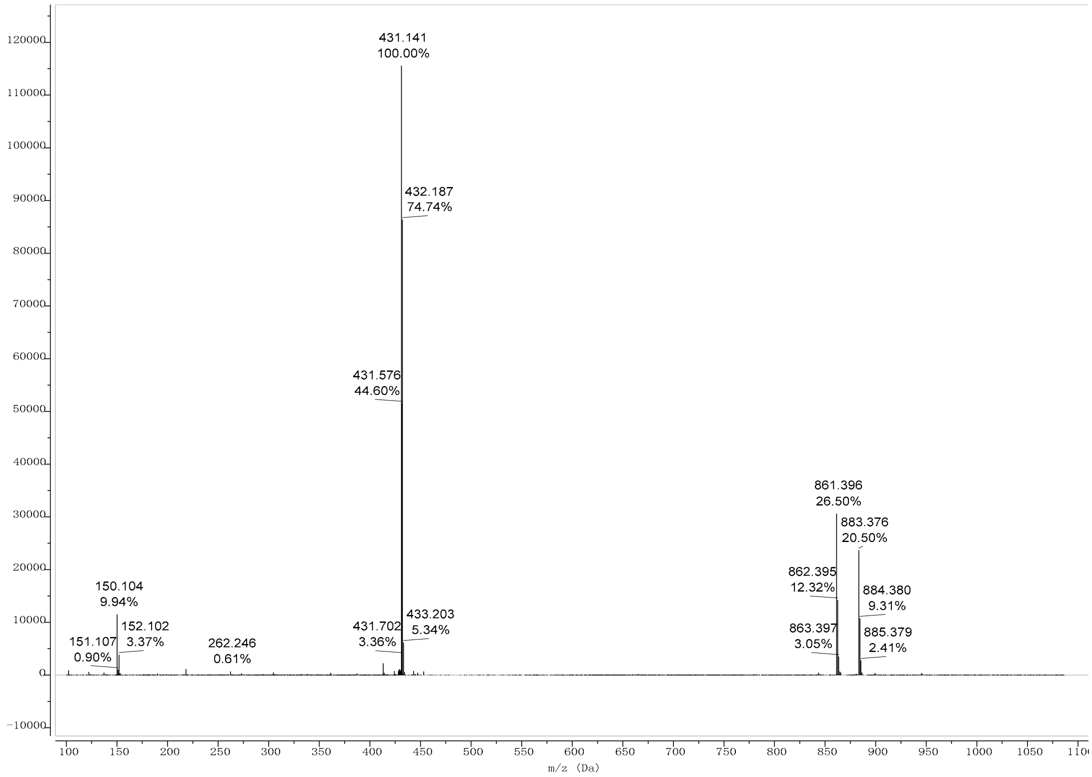

Figure S60. MS for $C_{19}H_{23}FN_8O_3$ (Mwt.: 430.44): m/z 431.141 ($[M+H]^+$, bp) of **7f**.

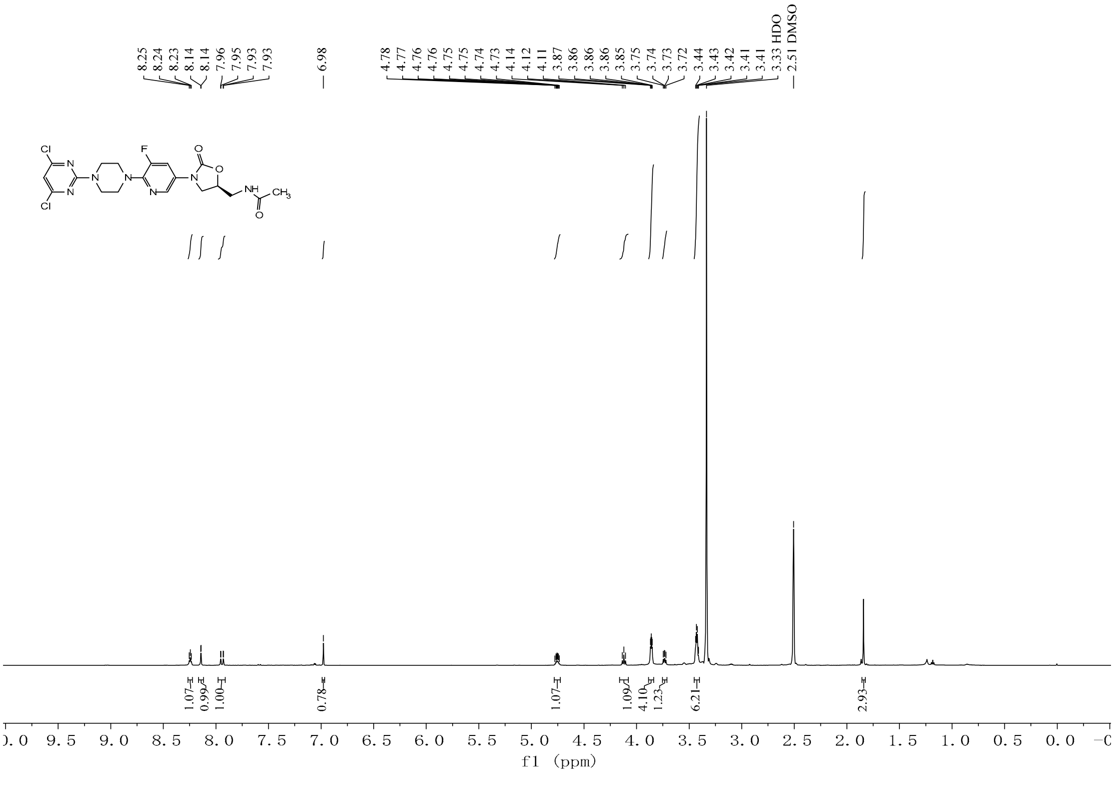

Figure S61. ^{1}H NMR Spectrum (DMSO-d_6, 600 MHz) of **7g**.

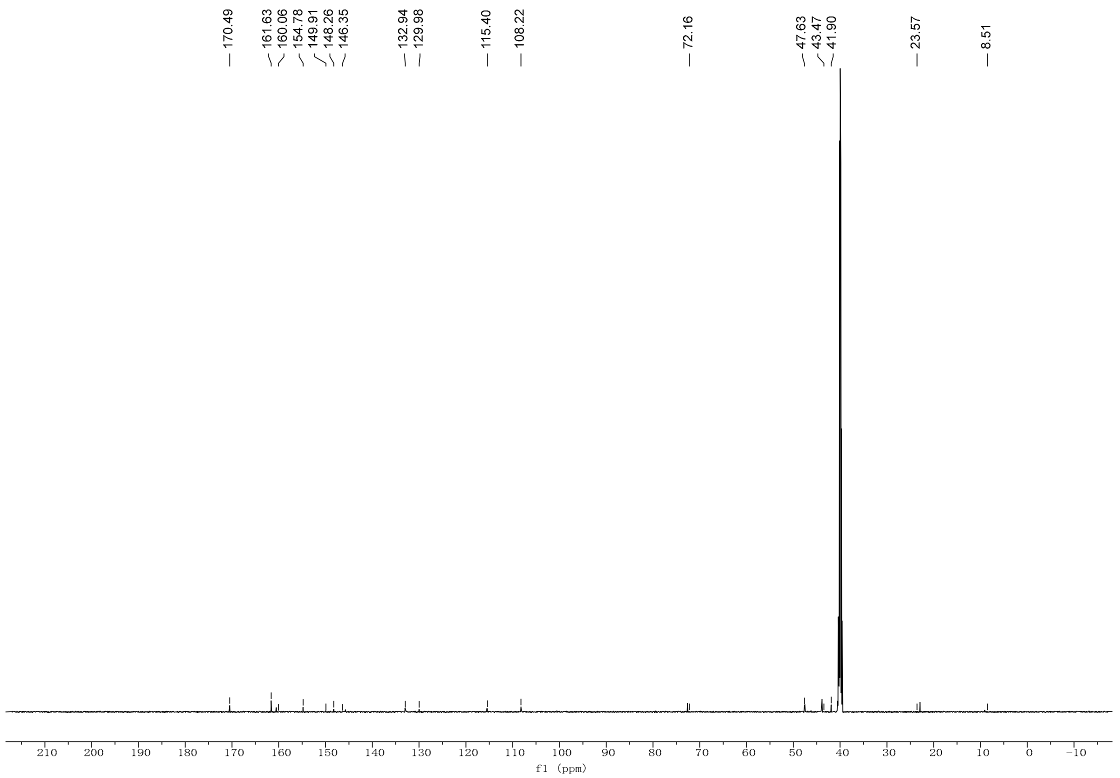

Figure S62. ^{13}C NMR Spectrum (DMSO-d_6, 150 MHz) of **7g**.

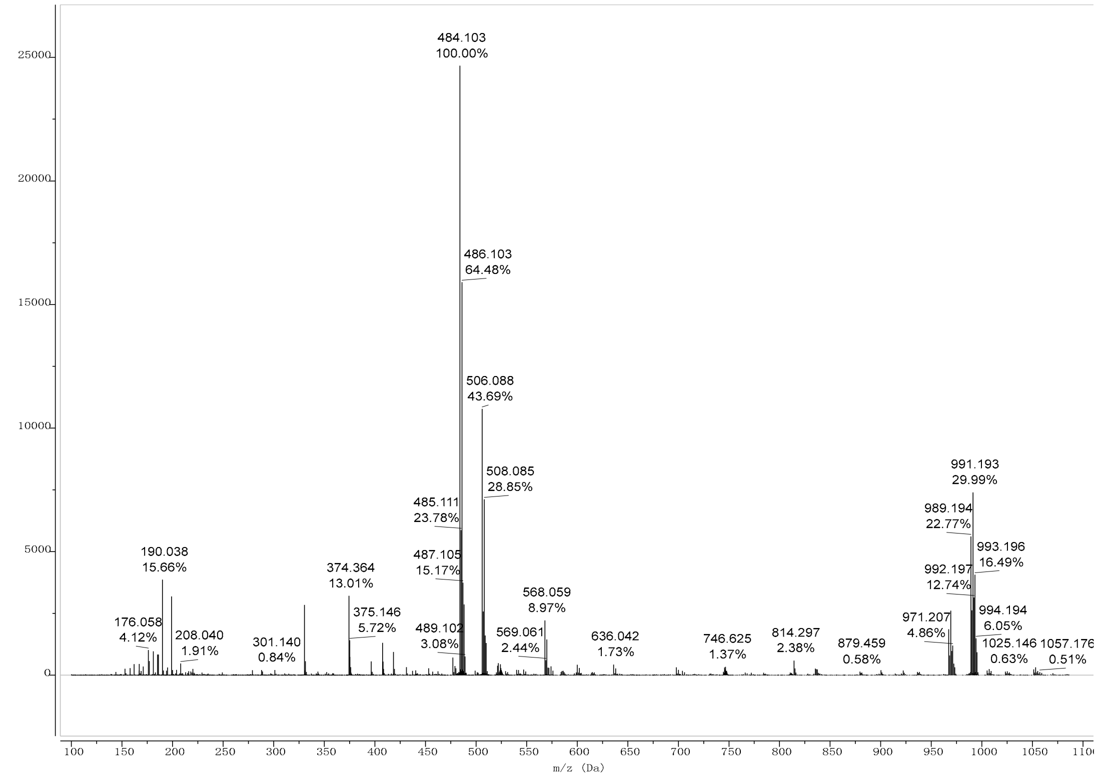

Figure S63. MS for $C_{19}H_{20}Cl_2FN_7O_3$ (Mwt.: 483.10): m/z 484.103 ([M+H] $^+$, bp) of **7g**.

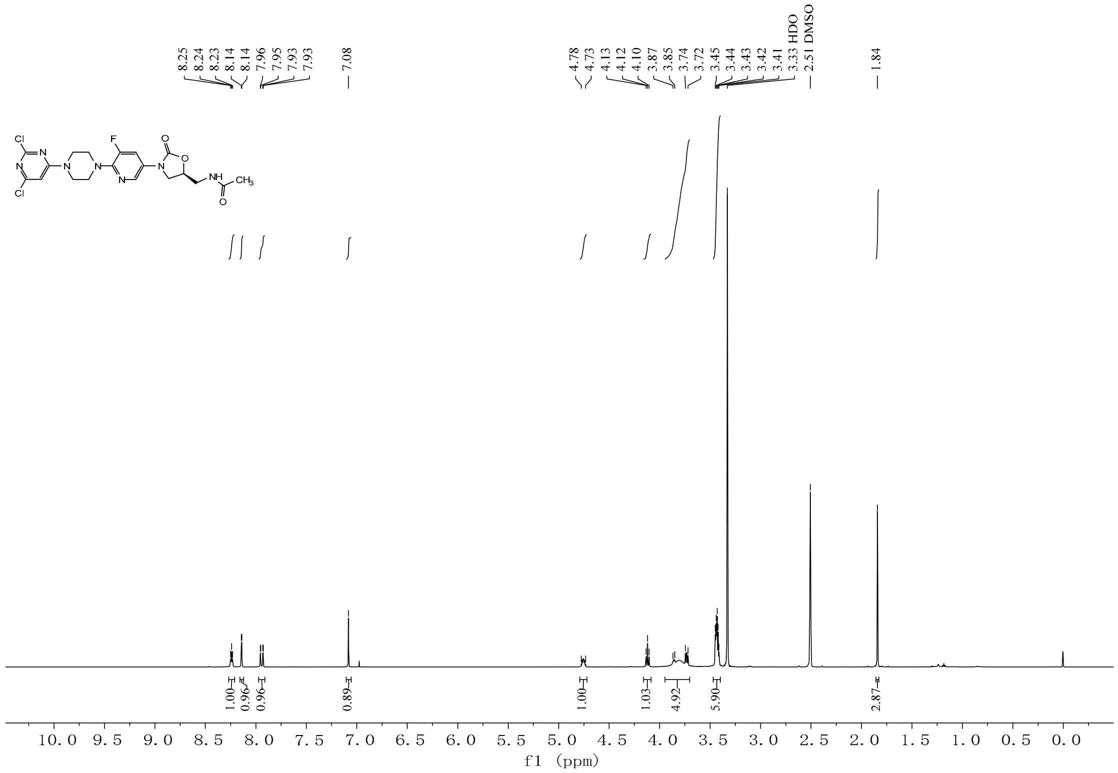

Figure S64. ^{1}H NMR Spectrum (DMSO-d_6, 600 MHz) of **7h**.

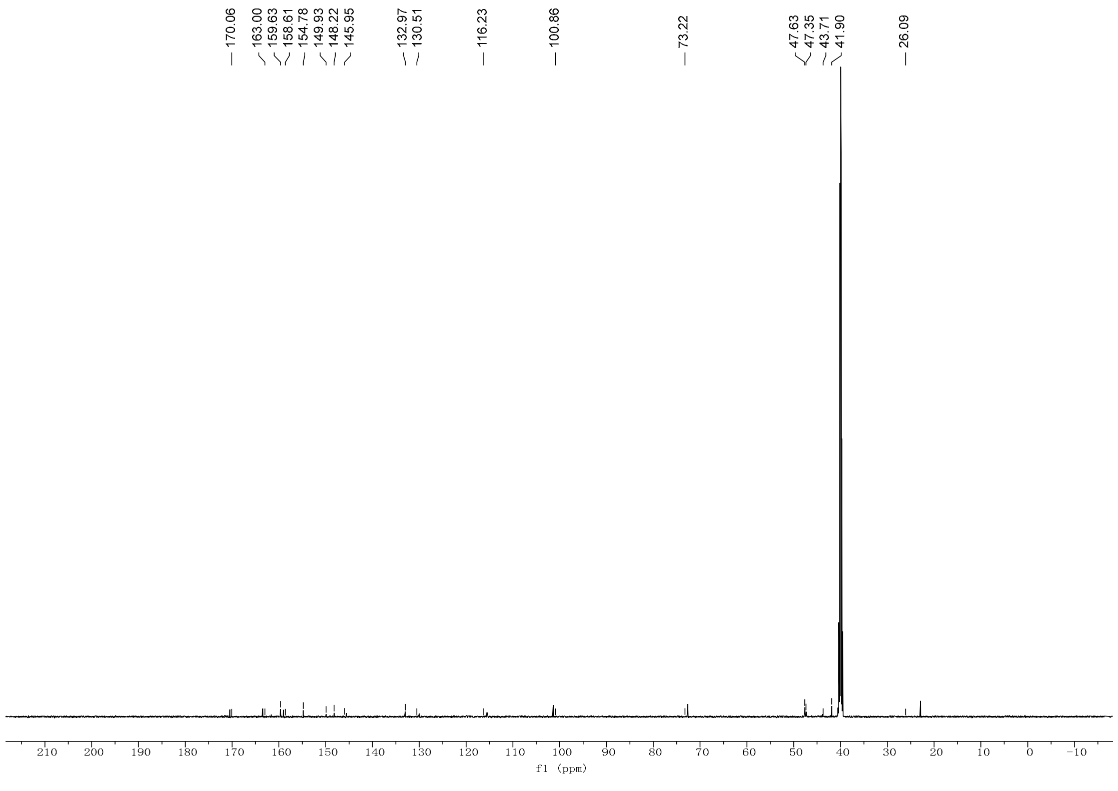

Figure S65. ^{13}C NMR Spectrum (DMSO-d_6, 150 MHz) of **7h**.

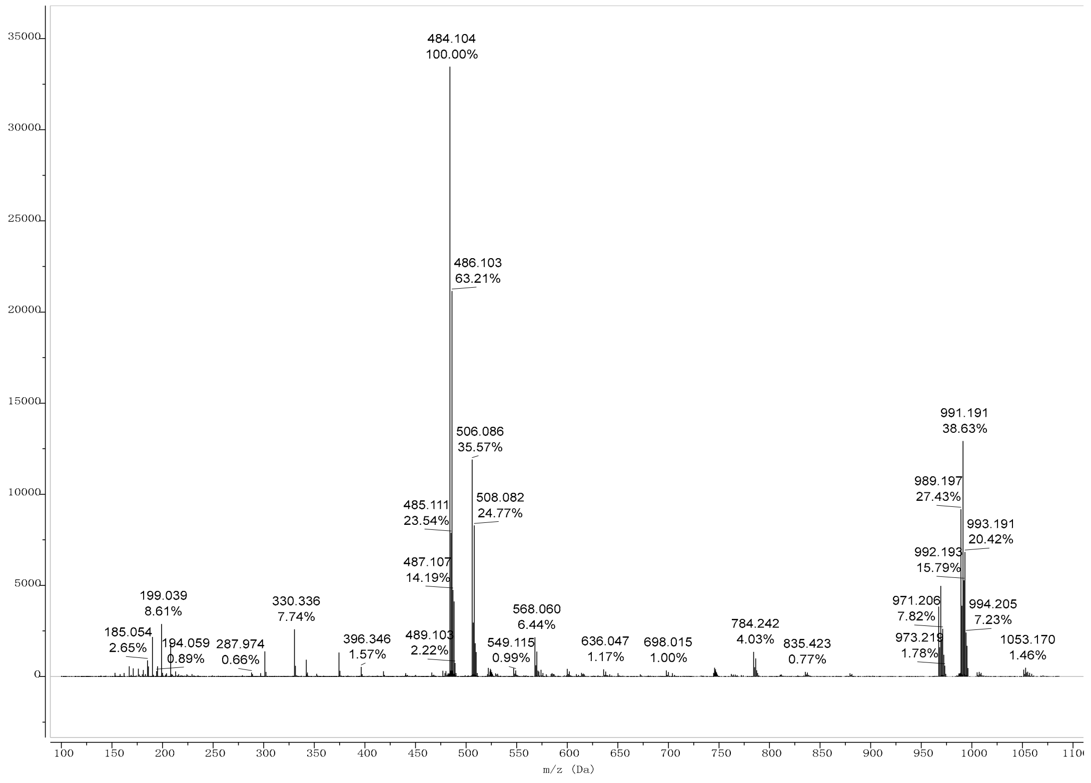

Figure S66. MS for $C_{19}H_{20}Cl_2FN_7O_3$ (Mwt.: 483.10): m/z 484.104 ($[M+H]^+$, bp) of **7h**.

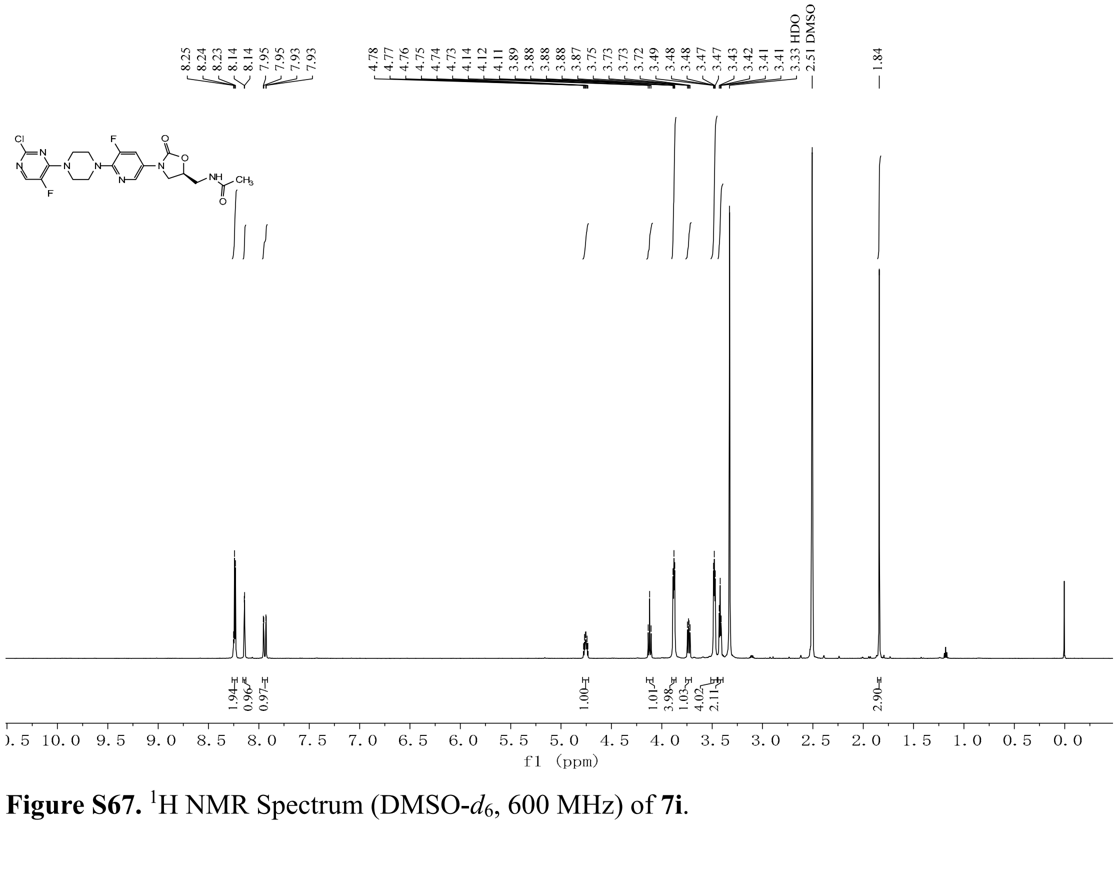

Figure S67. ^{1}H NMR Spectrum (DMSO-d_6, 600 MHz) of **7i**.

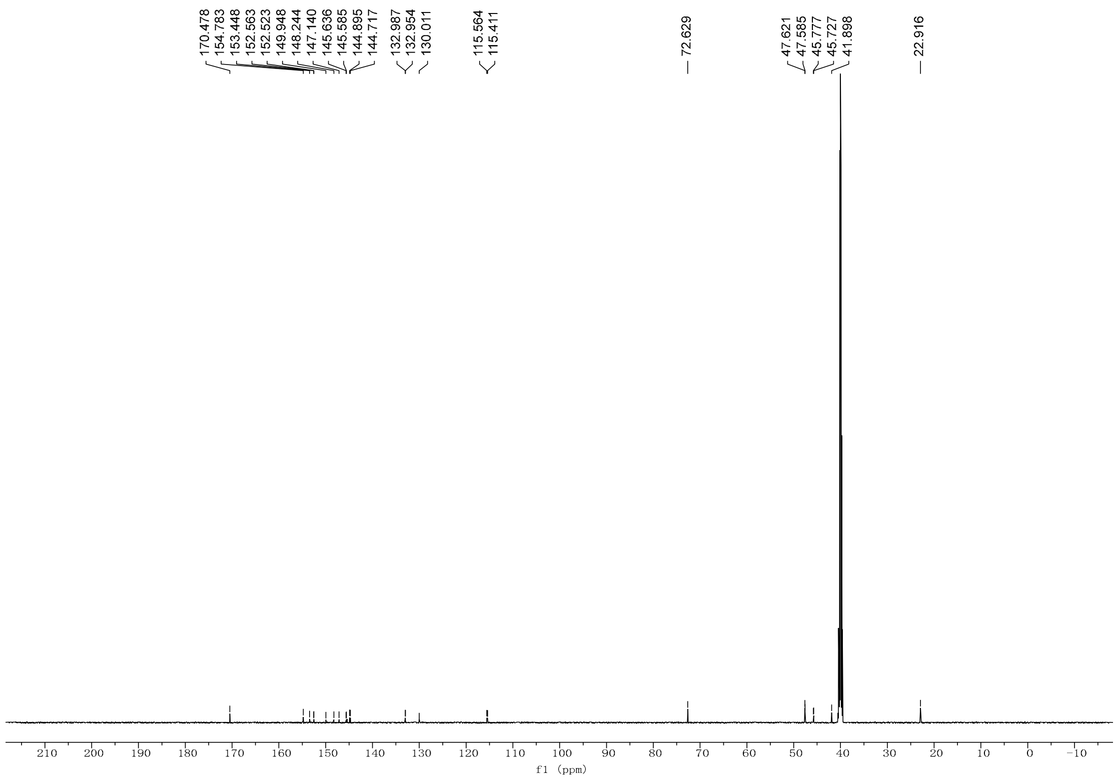

Figure S68. ^{13}C NMR Spectrum (DMSO-d_6, 150 MHz) of **7i**.

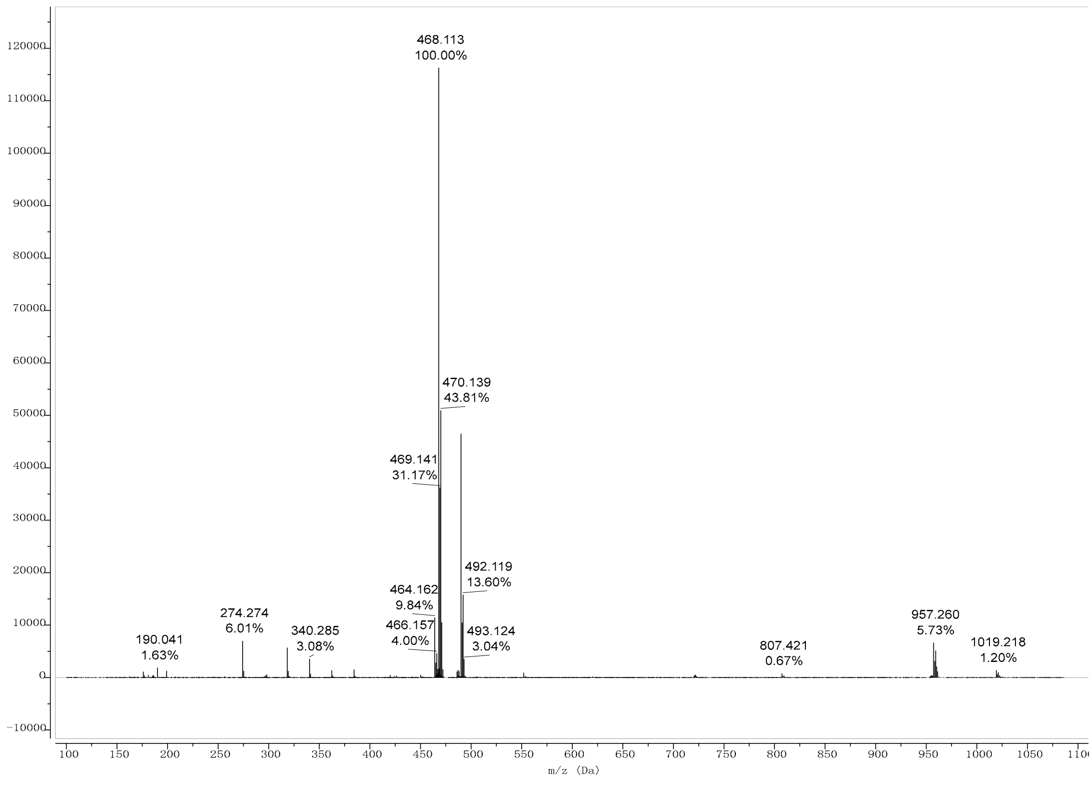

Figure S69. MS for $C_{19}H_{20}ClF_2N_7O_3$ (Mwt.: 467.86): m/z 468.113 ($[M+H]^+$, bp) of **7i**.

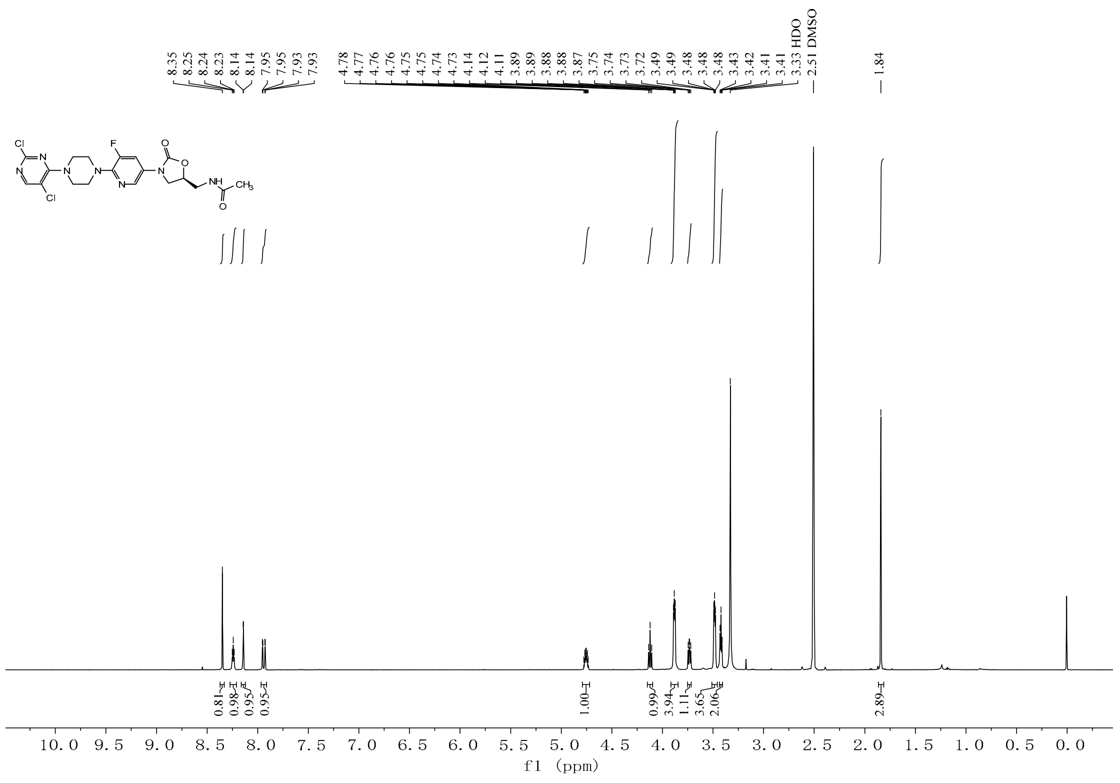

Figure S70. ^{1}H NMR Spectrum (DMSO-d_6, 600 MHz) of **7j**.

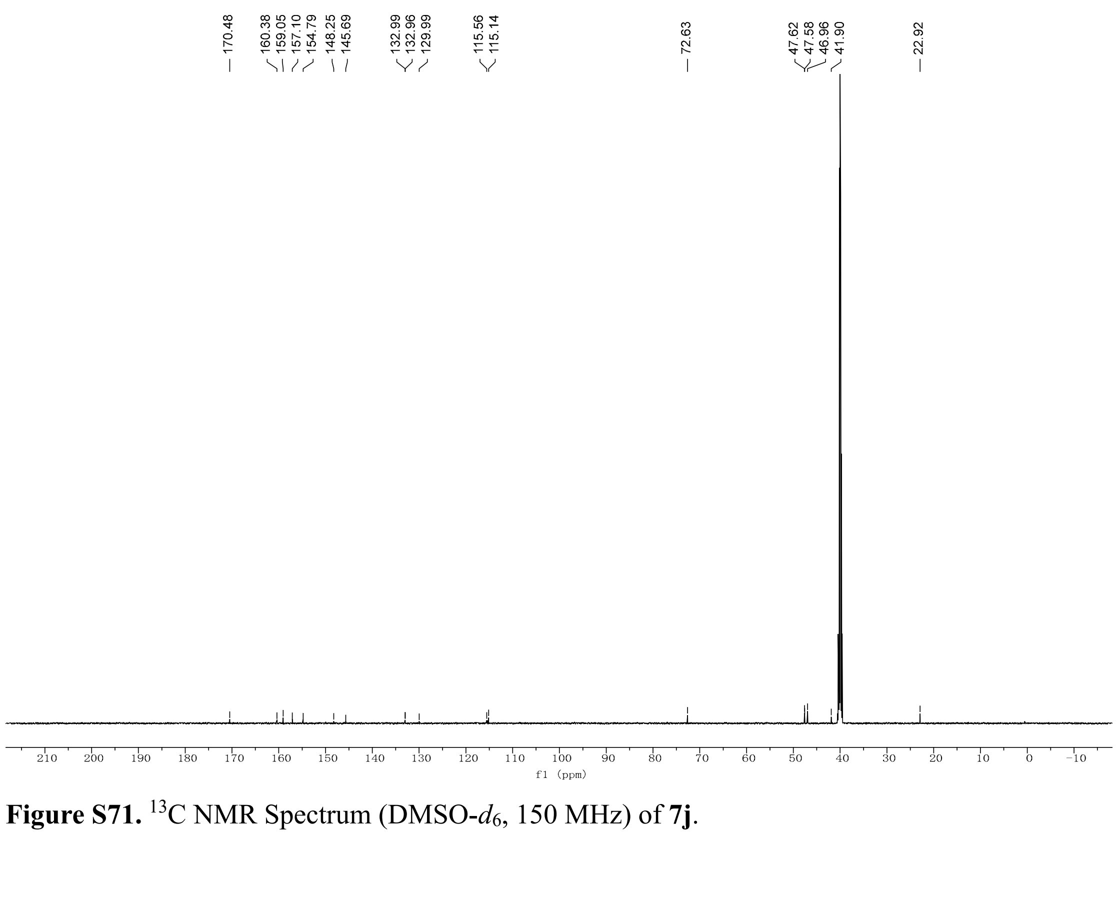

Figure S71. ^{13}C NMR Spectrum (DMSO-d_6, 150 MHz) of **7j**.

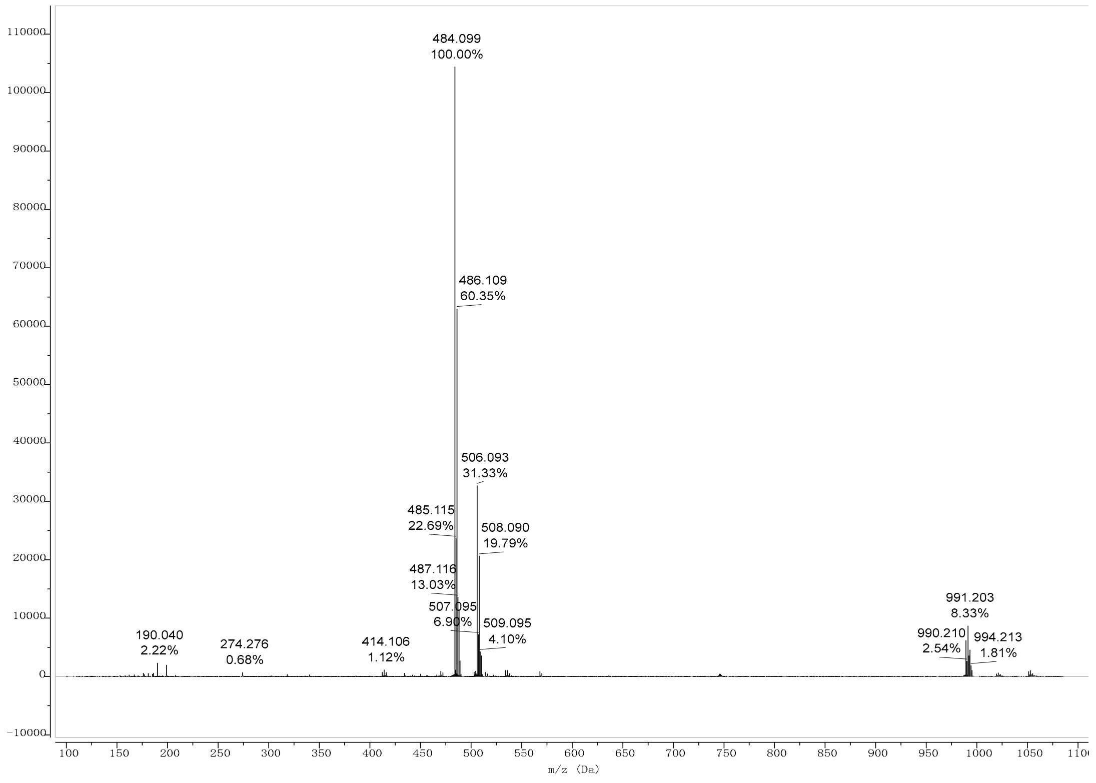

Figure S72. MS for $C_{19}H_{20}Cl_2FN_7O_3$ (Mwt.: 483.10): m/z 484.099 ([M+H] $^+$, bp) of **7j**.

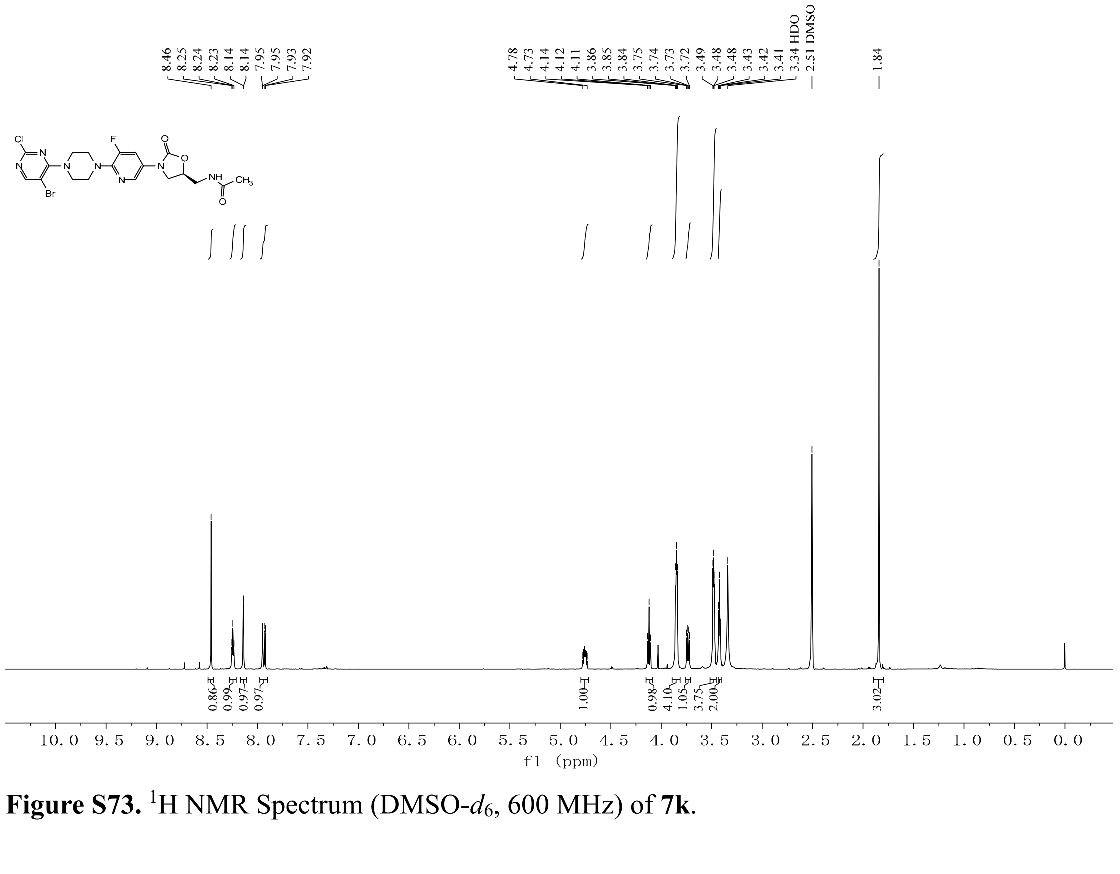

Figure S73. ^{1}H NMR Spectrum (DMSO-d_6, 600 MHz) of **7k**.

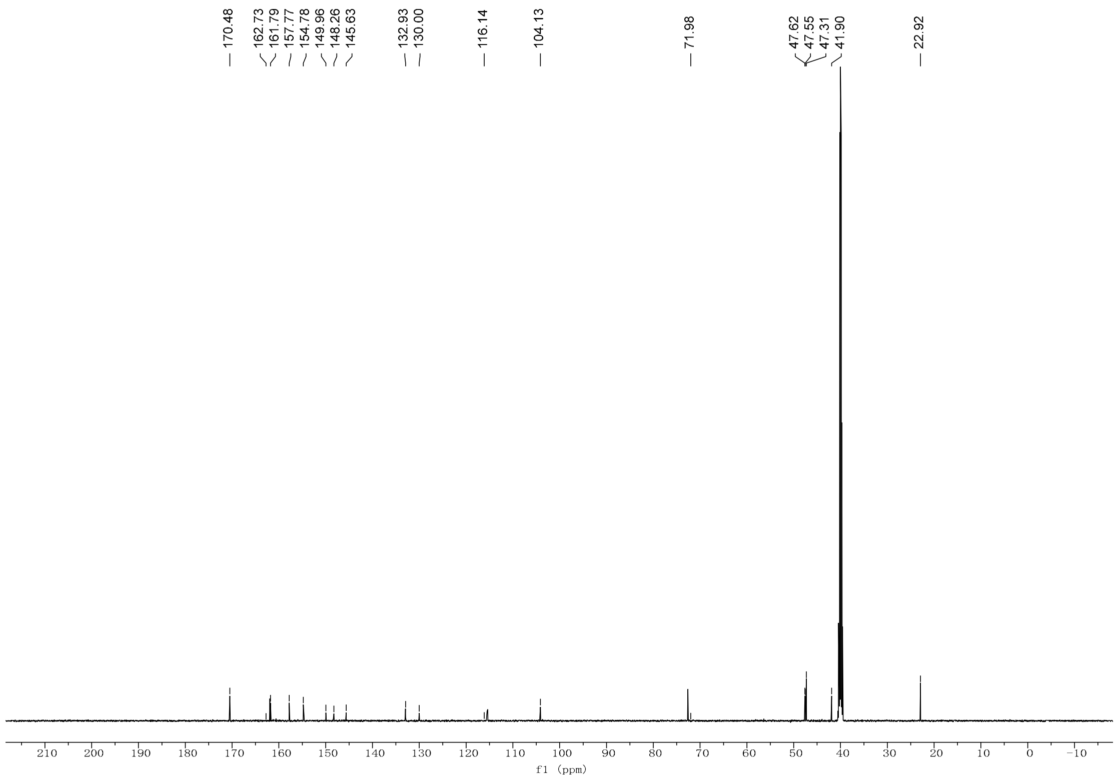

Figure S74. ^{13}C NMR Spectrum (DMSO-d_6, 150 MHz) of **7k**.

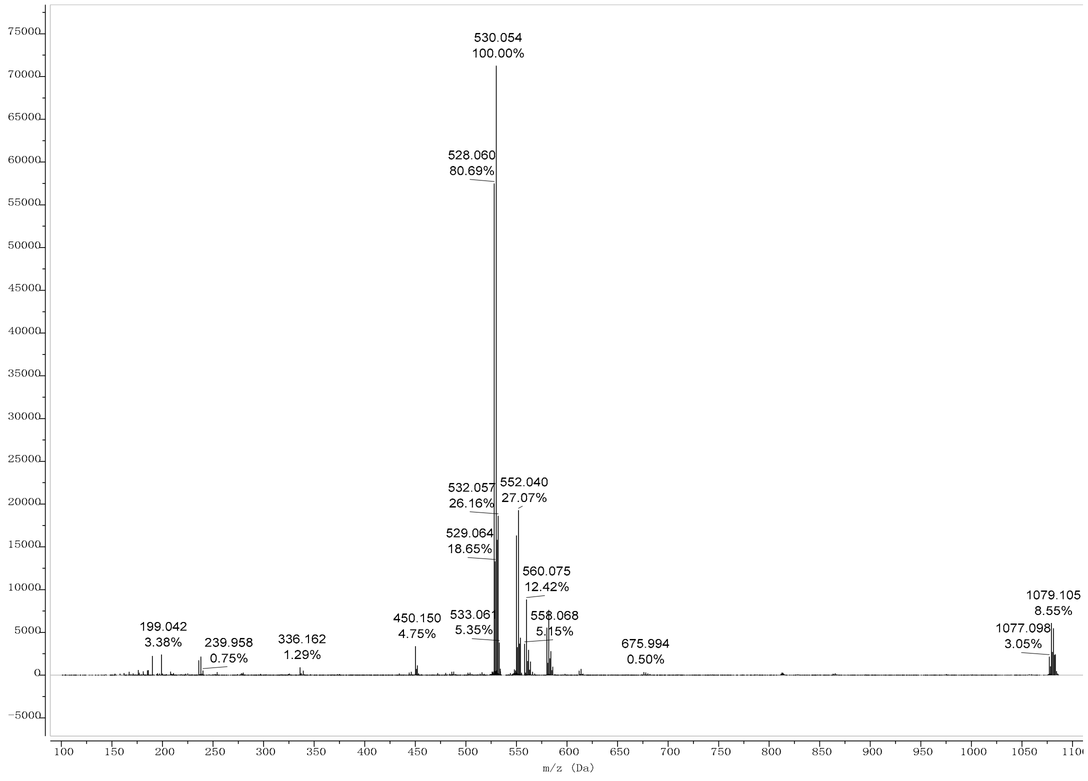

Figure S75. MS for $C_{19}H_{20}BrClFN_7O_3$ (Mwt.: 528.77): m/z 530.054 ([M+H] $^+$, bp) of **7k**.

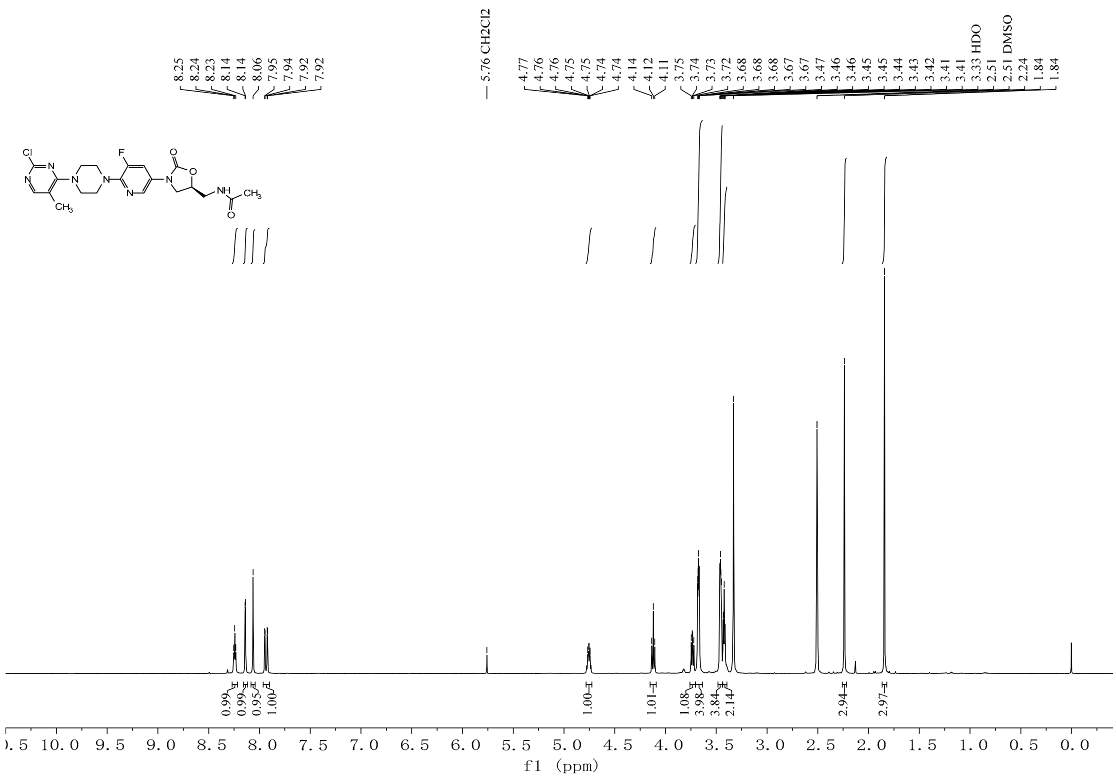

Figure S76. ^{1}H NMR Spectrum (DMSO-d_6, 600 MHz) of **7l**.

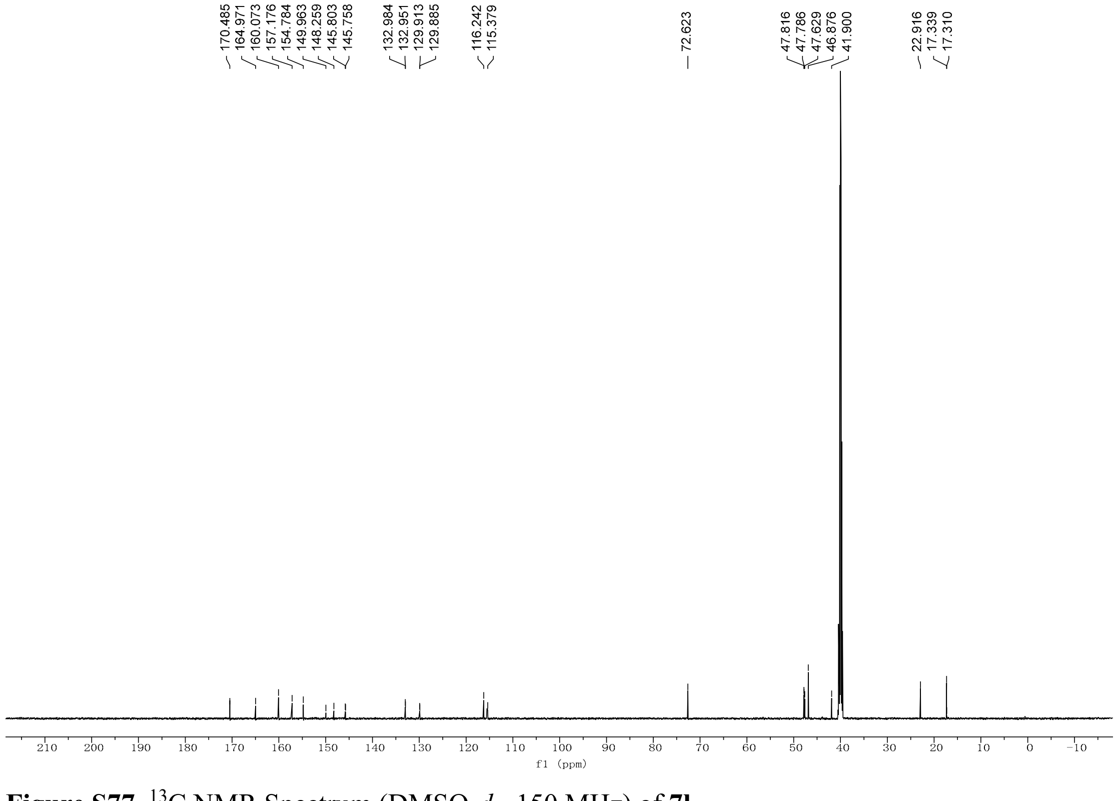

Figure S77. ^{13}C NMR Spectrum (DMSO-d_6, 150 MHz) of **7l**.

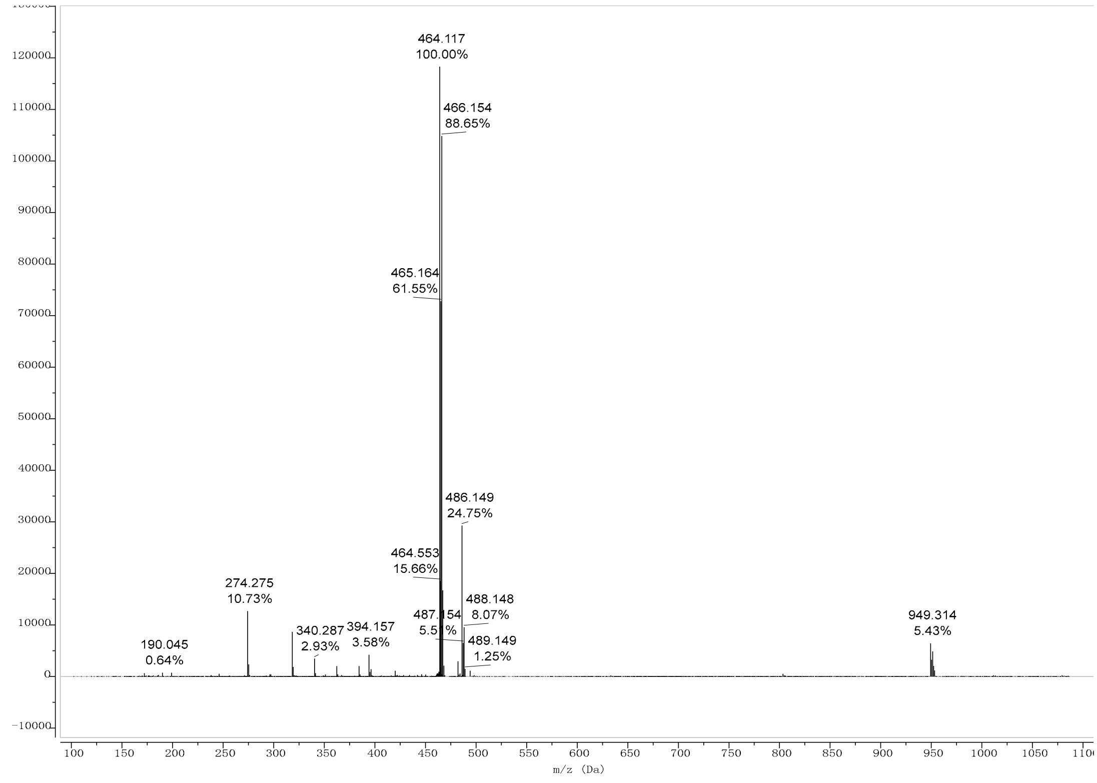

Figure S78. MS for $C_{20}H_{23}ClFN_7O_3$ (Mwt.: 463.90): m/z 464.117 ([M+H] $^+$, bp) of **7l**.

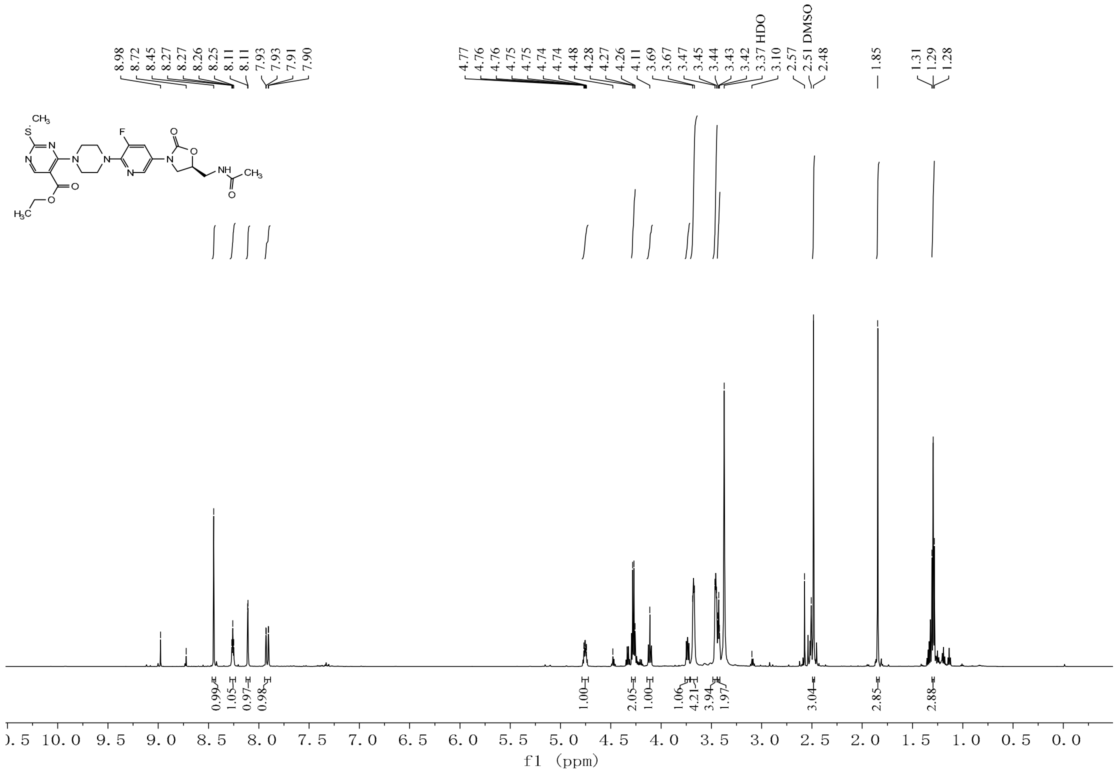

Figure S79. ^{1}H NMR Spectrum (DMSO-d_6, 600 MHz) of **7m.**

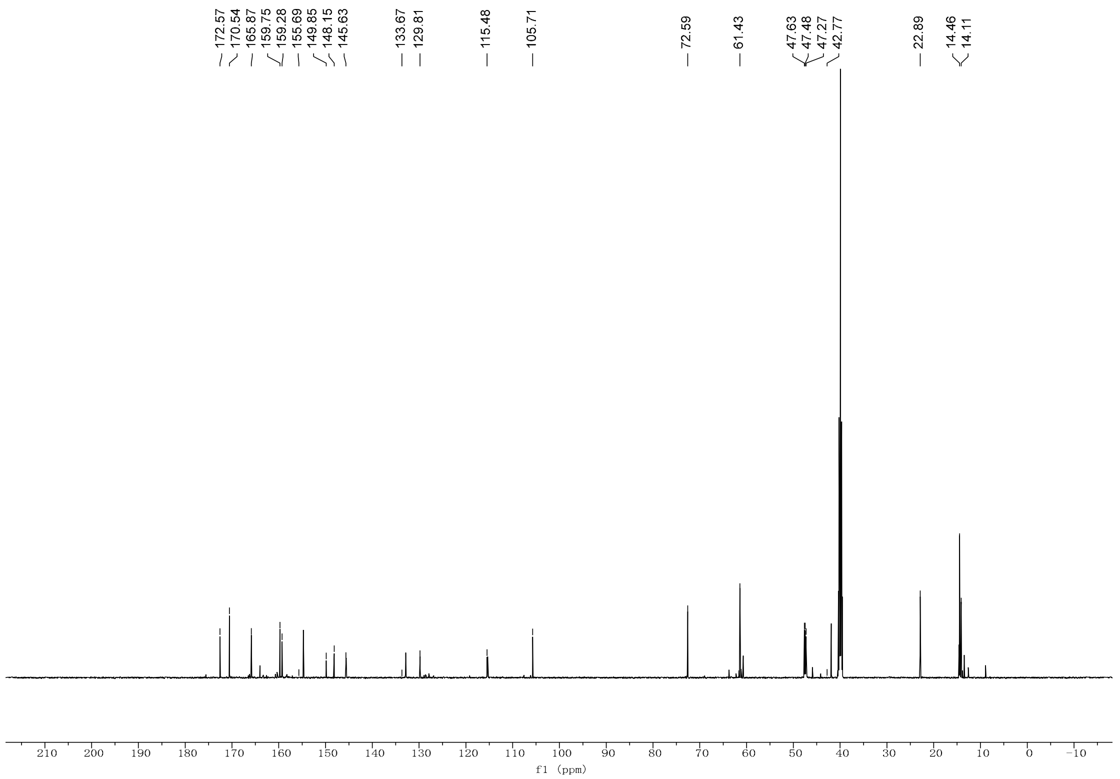

Figure S80. ^{13}C NMR Spectrum (DMSO-d_6, 150 MHz) of **7m**.

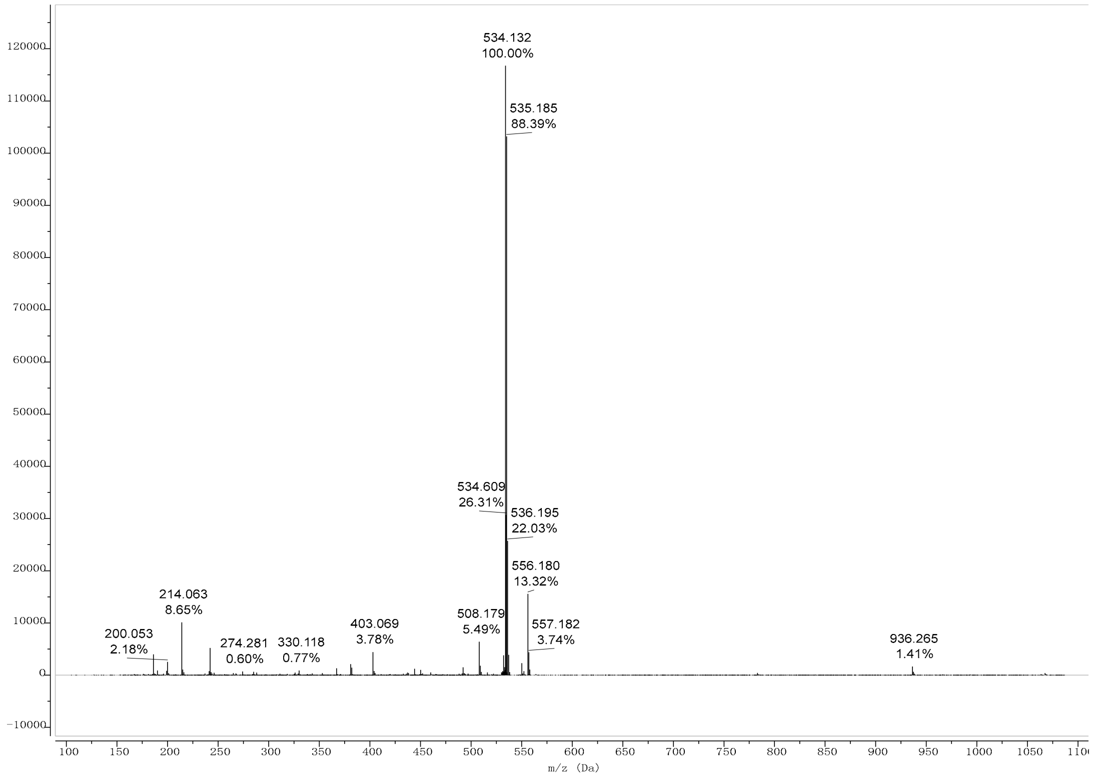

Figure S81. MS for $C_{23}H_{28}FN_7O_5S$ (Mwt.: 533.58): m/z 534.132 ([M+H] $^+$, bp) of **7m**.

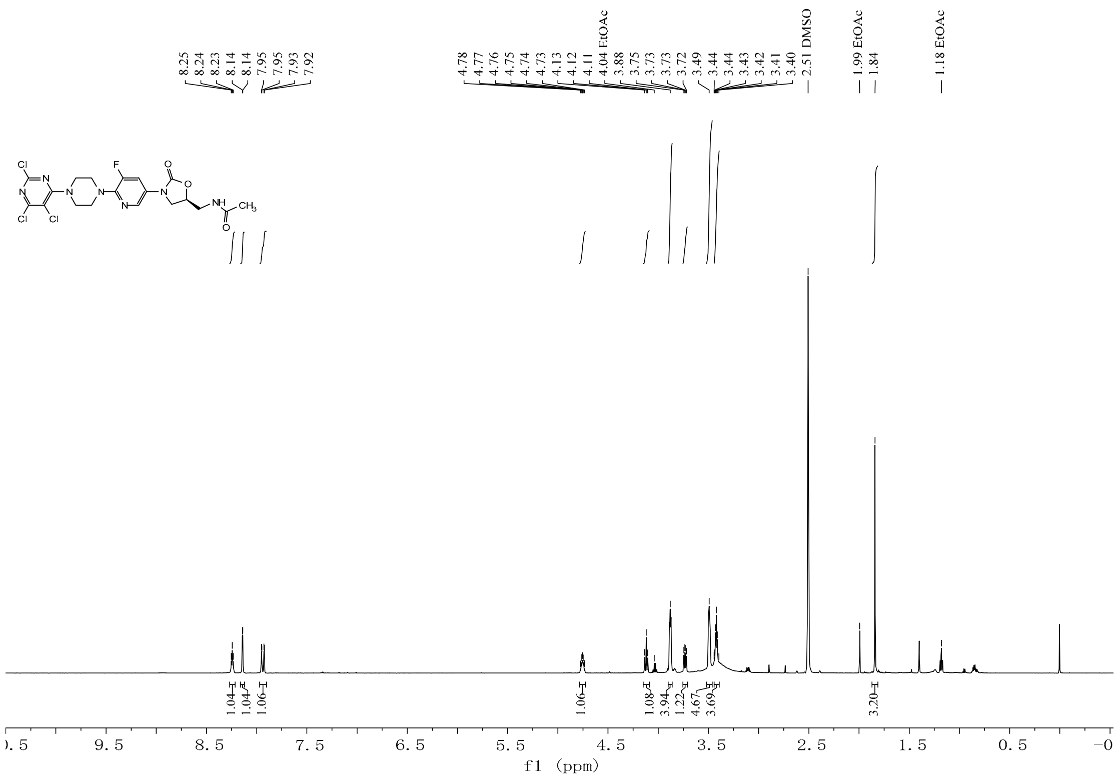

Figure S82. ^{1}H NMR Spectrum (DMSO-d_6, 600 MHz) of **7n**.

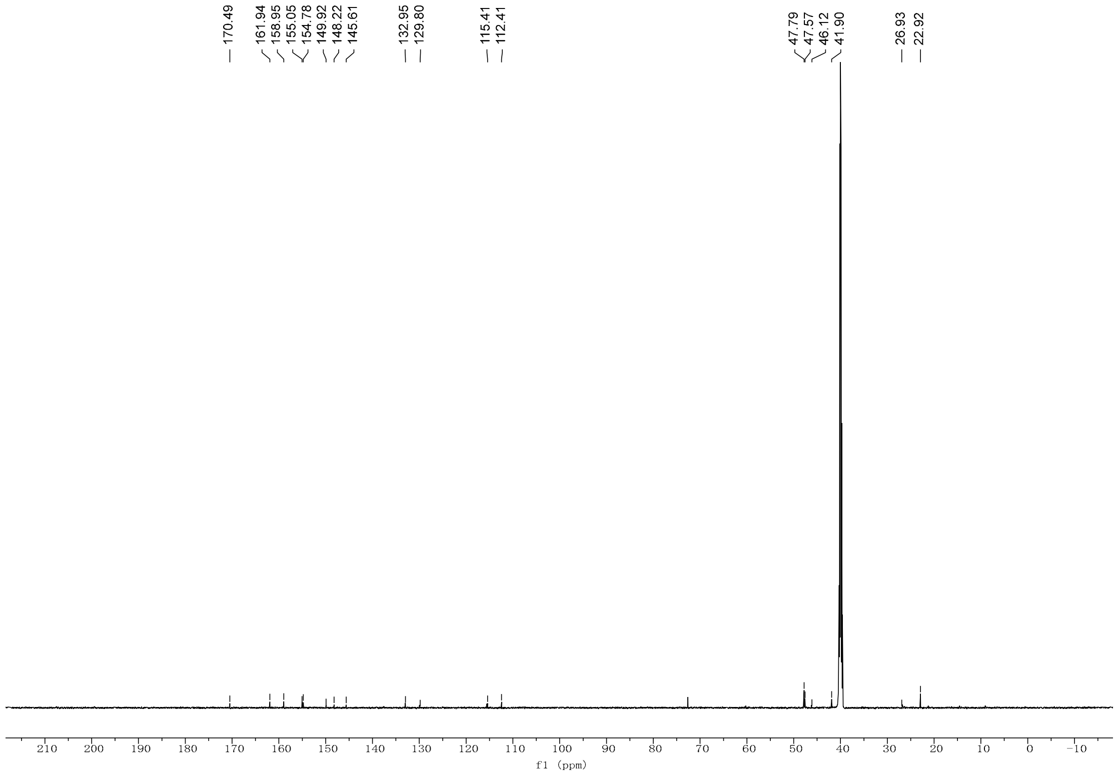

Figure S83. ^{13}C NMR Spectrum (DMSO-d_6, 150 MHz) of **7n**.

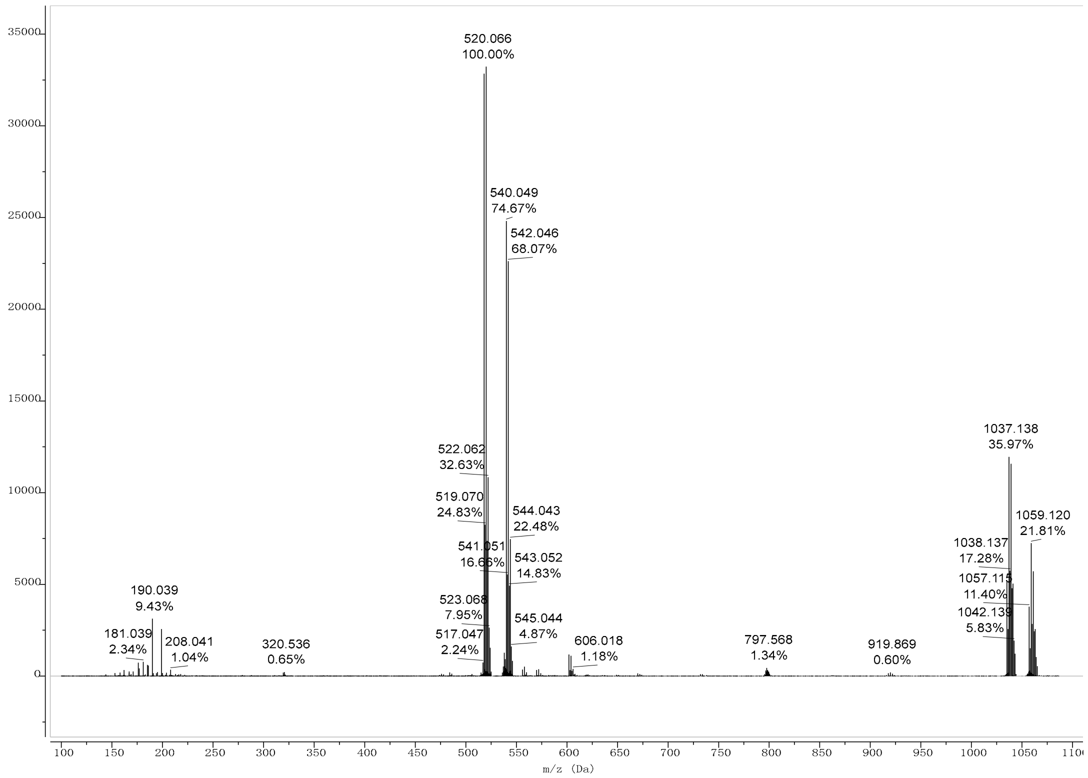

Figure S84. MS for $C_{19}H_{19}Cl_3FN_7O_3$ (Mwt.: 518.76): m/z 520.066 ([M+H] $^+$, bp) of **7n**.
